# Lifecycle DoE—The Companion for a Holistic Development Process

**DOI:** 10.3390/bioengineering11111089

**Published:** 2024-10-30

**Authors:** Marco Kunzelmann, Anja Wittmann, Beate Presser, Philipp Brosig, Pia Kristin Marhoffer, Marlene Antje Haider, Julia Martin, Martina Berger, Thomas Wucherpfennig

**Affiliations:** 1 Development Biologicals, Boehringer Ingelheim Pharma GmbH & Co. KG, Birkendorferstraße 65, 88397 Biberach an der Riß, Germanybeate.presser@boehringer-ingelheim.com (B.P.); thomas.wucherpfennig@boehringer-ingelheim.com (T.W.); 2 HP BioP Operations Network Mammalian, Boehringer Ingelheim Pharma GmbH & Co. KG, Birkendorferstraße 65, 88397 Biberach an der Riß, Germany

**Keywords:** design of experiments, holistic experimental design, design augmentation, quality by design, optimization and robustness analysis, process characterization study, bioprocess development, CMC development

## Abstract

Within process development, numerous experimental studies are undertaken to establish, optimize and characterize individual bioprocess unit operations. These studies pursue diverse objectives such as enhancing titer or minimizing impurities. Consequently, Design of Experiment (DoE) studies are planned and analyzed independently from each other, making it challenging to interlink individual data sets to form a comprehensive overview at the conclusion of the development process. This paper elucidates the methodology for constructing a Life-Cycle-DoE (LDoE), which integrates data-driven process knowledge through design augmentations. It delves into the strategy, highlights the challenges encountered and provides solutions for overcoming them. The LDoE approach facilitates the augmentation of an existing model with new experiments in a unified design. It allows for flexible design adaptations as per the requirements of subject matter experts (SME) during process development, concurrently enhancing model predictions by utilizing all available data. The LDoE boasts a broad application spectrum as it consolidates all data generated within bioprocess development into a single file and model. The study demonstrates that the LDoE approach enables a process characterization study (PCS) to be performed solely with development data. Furthermore, it identifies potentially critical process parameters (pCPPs) early, allowing for timely adaptations in process development to address these challenges.

## 1. Introduction

DoE approaches for process development are not new in the pharmaceutical industry [1,2,3,4] and are extensively utilized throughout the product process development lifecycle [5,6]. The advantages of DoEs over one factor at a time (OFAT) experiments, where only a single process parameter (PP) is examined while all others remain constant, have been demonstrated by Kasemiire et al. [7] and Beg et al. [8]. The Process Analytical Technology (PAT) guidelines introduced by the U.S. Food and Drug Administration (FDA) in 2004 [9] and the Quality by Design (QbD) initiative launched by the European Medicine Agency (EMA) with the ICH Q8 in 2009 [10,11], have further underscored the relevance and necessity of DoEs in the pharmaceutical industry [12]. Comprehensive summaries on this topic have been provided by Little [13] and Fukuda et al. [14].

A key component of the QbD process is a risk assessment to identify pCPPs, often conducted through a failure mode and effect analysis (FMEA) [15]. This step relies on knowledge derived from process development, which typically includes a blend of experimental data and subject matter expert (SME) knowledge. However, it was observed that experiments during process development are often not conducted in an aligned manner. Rather, each development work package (WP) has distinct objectives and investigates different PPs.

Generally, each unit operation during process development is divided into several development WPs, where DoEs are performed for various optimization and robustness objectives. Despite the widespread use of DoEs across these WPs for efficiency, they are designed independently from existing and analyzed DoEs. Consequently, each DoE begins from scratch and information is not interconnected between WPs. Furthermore, the investigated PP spaces per WP overlap only minimally or sometimes not at all. This lack of overlap prevents the integration of individual models per WP into one comprehensive model, resulting in fragmented prior knowledge. This fragmentation leads to an increased number of experiments and it becomes challenging to assess the criticality of a PP during the FMEA, as the parameter effects across the WPs are often not comparable.

Few studies have been published on the integration of DoEs using the entire development process for efficiency gain, rather than merely individual development WPs. Shekhawat et al. [16] proposed a split DoE method, where instead of performing one DoE containing all pCPPs, it is divided into two: one containing dominant PPs and the other, non-dominant PPs. The classification of the PP is based on process knowledge. While this approach indeed reduces the total number of required experiments, as all dominant PPs are kept on fixed optimized settings in the second DoE, it neglects all interaction effects between dominant and non-dominant PPs. This results in an incomplete design space and limits the intended usage of the QbD approach.

Zahel et al. developed a methodology for an Integrated Process Model (IPM) [17], where models from several unit operations are combined to assess the impact of PPs on the complete bioprocess, rather than just a single unit operation. Oberleitner et al. [18] made this approach more efficient by creating an algorithm that identifies the most important experiments for the IPM. This allows existing DoEs to be augmented with fewer experiments to achieve the main goals in process development. However, for the efficient usage of the IPM, models need to be available, at least for the most important unit operations in the biopharmaceutical process. Additionally, the final specification limits on the last unit operation need to be known to benchmark the results of the IPM in terms of out-of-specification limits. Both are usually not available within chemical manufacturing and control (CMC) development, making the applicability of an IPM, especially for an efficient design augmentation, difficult at the early stages of process development.

During the global SARS-CoV-2 pandemic, traditional development concepts were challenged and new, out-of-the-box concepts were required to enable warp speed development and industrialization of potentially lifesaving medicines [19,20,21,22]. Xu and colleagues describe different strategies to shorten the CMC development timeline for upstream process development of two antibodies, leading to a complete PCS in just 4 months [23]. With the approach introduced in this paper, project accelerations should be further supported. A workflow that allows augmenting and improving an existing initial model with further DoE experiments is introduced, enabling a model-based knowledge transfer within the product lifecycle. Unlike the IPM, the LDoE approach does not consider the complete biopharmaceutical process; instead, the approach is used per unit operation. This makes the approach independent from the development stages of previous and subsequent unit operations and provides the flexibility needed within the early stages of process development. It should be emphasized that the LDoE is not intended as a replacement for existing approaches such as the IPM. Instead, the LDoE is a complementary part, improving applicability at later-stage process development.

The focus of this paper is not on the development of new statistical methods. Instead, existing methods are used and combined in a smart way, always with the focus on establishing a holistic development model that incorporates the combined knowledge of all development data. During the LDoE process, a continuous refinement of the design space takes place, always with the objective to find PP settings leading to optimal critical quality attribute (CQA) outputs. Simultaneously, the requirements of the process characterization study (PCS), where proven acceptable ranges (PARs) need to be identified that are at least as wide as the normal operating ranges (NORs), need to be fulfilled. A profound strategy for a PCS analysis and how PARs can be derived from LDoE models is demonstrated. Moreover, a PP criticality assessment was developed to identify pCPPs early in process development where design space adjustments are still possible rather than at the end of the process cycle. Our approach is inspired by the impact ratio introduced by Hakemeyer et al. [24] but was adjusted to our PCS strategy, which considers model and PP uncertainties. Furthermore, it is demonstrated that our approach fulfills the requirements from FDA and EMA to establish a complete QbD for all investigated PPs and supports the calculation of appropriate limits for the critical quality attributes (CQAs).

Aligning many WPs over the extensive timeline of bioprocess development presents a significant challenge. The aim of this paper is to guide readers on the successful implementation of the LDoE approach into the routine workflow of biopharmaceutical process development. Potential challenges are highlighted, strategies to overcome them are suggested and a checklist of key considerations is provided.

## 2. Materials and Methods

### 2.1. Optimal Designs

Classical DoEs are an efficient method for creating experimental plans [4,25,26,27,28]. Fractional factorial designs are used to screen for the most important PPs. Response surface designs, including Box–Behnken Designs or Central Composite Designs (CCD) are utilized to generate a model for predicting combinations of PP settings within the minimum and maximum range of the respective PP, also known as the design space. A comprehensive discussion of these designs is available in [29].

However, in practice, these designs are not universally applicable. They necessitate a specific number of experiments based on the number of PPs being investigated and cannot incorporate existing data [30]. For instance, a CCD with four input parameters at three distinct settings (levels) requires 2p + 2p = 24 experiments, plus n experiments at the center of the design space (center point experiments).

In contrast, optimal designs offer more flexibility in determining the number of runs, as they do not require strict orthogonality [31,32,33,34]. This flexibility is particularly beneficial in process development. Moreover, optimal designs can augment an existing set of experiments. Both properties are crucial for their application in LDoE.

In this paper, the D-optimal criterion was used. D-optimal designs minimize the variance of input parameter estimates in a model [30] (pp. 33–40). The D-optimal criterion was predominantly used as it efficiently detects critical process parameters (CPP). However, it should be highlighted, that it also may be beneficial to augment using I-optimality to enhance model prediction within the investigated design space [30] (pp. 88–90) or other optimality criteria.

### 2.2. Lifecycle DoE

**Figure 1:**
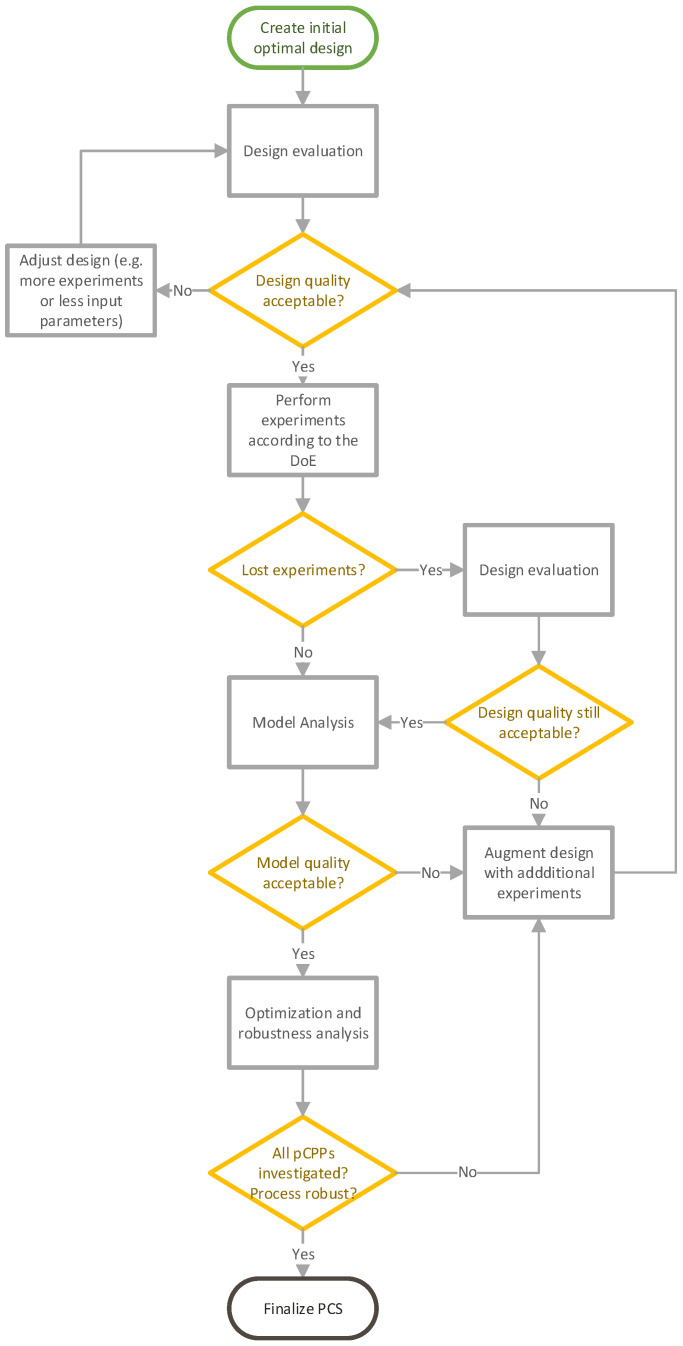
LDoE Workflow.

The LDoE is based on an initial optimal DoE and is augmented by additional optimal designs. This approach begins with a small set of experiments that is expanded in each development WP. Based on the results of previously conducted experiments, new input parameters are added, or the ranges of existing input parameters are adjusted. This approach can be understood as a frequentist way to incorporate prior knowledge comparable to Bayesian approaches. In each augmentation cycle, a design evaluation, model analysis, robustness and optimization analysis was performed. This iterative cycle was repeated until all pCPPs were investigated and optimal and robust process settings were found. Figure 1 gives a detailed overview of the LDoE workflow.

### 2.3. Design Analysis

The here described DoEs follow the good principles of experimental designs described in [35], e.g., experiments are randomized, different work packages (WP) within process development are considered as blocks and replicates are performed to consider process and analytical variability. Furthermore, the design quality was investigated in form of a statistical power analysis.

#### Power Analysis

The flexibility regarding the sample size in optimal designs comes with a cost, it is necessary to be evaluated if the design quality fulfill the criteria for the corresponding study. One of the most fundamental design characteristics is the statistical power [36,37] (pp. 836–839). The statistical power is the probability of detecting a critical effect size $\beta_{crit,i}$ of an input parameter given the error variance of the model $\sigma_{\varepsilon }^{2}$ and a false-positive rate α. Usually, the cutoff criterion for alpha is set to 5% and the critical effect size and error variance are unknown. It is easier to define a signal-to-noise ratio $\frac{\beta_{i}}{\sigma_{\varepsilon }}$ which needs to be detected [30] (pp. 21–29).

From a practical perspective, signal-to-noise ratios greater than three, i.e., effects greater than three times the standard deviation (SD) of the CQA are critical. This corresponds to a coefficient size of 1.5 for all main effects, two-factor interactions and cubic terms (i.e., coding from −1 to +1) and a coefficient size of 3.0 for all quadratic terms (i.e., coding from 0 to +1). A common consensus is that a power of around 80% should be achieved [35]. The statistical power can be increased with a higher sample size n and a low correlation structure between the investigated input parameters. In simpler terms, one needs to ascertain whether the selected sample size and the correlation structure between the PPs is sufficient to detect a critical effect on the CQA, if it is indeed present.

The power analyses were performed, always incorporating all main effects, two factor interactions and quadratic terms.

At later stages in process development some PPs were investigated on more than three levels and thus more complex effects could occur (e.g., cubic effects). Due to the ±1 scale coding of input parameters, a strong correlation exists between main effects and their corresponding cubic effects, independent from the number of available experiments. This results in a smaller power when both terms—main effect and the corresponding cubic effect—are included in the design analysis. However, this does not pose an issue as the model selection process does not adhere the heredity principle. The pruned forward algorithm in the model selection process can determine whether a main effect or a cubic effect for the same input parameter fits the data better (for further details see Model Analysis). Consequently, it is more rational to include only one of the two effects in the power analysis.

### 2.4. Model Analysis

This paper employs two distinct modeling techniques: Ordinary Least Square (OLS) and Linear Mixed Models (LMM). OLS models incorporate only fixed effects (i.e., PP effects) and are utilized for parameters that can be controlled, like temperature. On the other hand, LMMs are employed to differentiate fixed effects from random variations that are uncontrollable, using model terms derived from the chosen OLS models [38]. The block effect encapsulates the ‘random’ variability between WPs, caused by factors like different fermentation batches. By treating this block-to-block variability as a random effect, the model can estimate WP-to-WP variability and make predictions about future WPs. Reliable estimation of this variance component is crucial to assess the true process variability.

An in-depth model analysis was conducted to ensure the quality of the model, similar to the one proposed by the EMA Quality and Innovation Group in [39] for medium-risk models.

#### 2.4.1. Ordinary Least Square Models

The statistical models were developed using the Ordinary Least Squares method, taking into account all process parameter main effects, two factor interactions, quadratic effects and cubic effects from PPs with more than three levels. Data from multiple fermentation batches were used and potential differences were considered as fixed block effects. The statistical model for each CQA can be described as followed:

(1) $$Y=\beta_{0}+\sum_{i=1}^{p}\left(\beta_{i}x_{i}+\beta_{ii}x_{i}^{2}+\beta_{iii}x_{i}^{3}\right)+\sum_{i<j}^{p}\beta_{ij}x_{i}x_{j}+\sum_{i=1}^{b-1}\gamma_{i}s_{i}+\varepsilon ,$$

where:

- Y is the observed output parameter (e.g., critical quality attribute)
- $\beta_{0}$ is the model intercept
- $\beta_{i}$ is the ith main effect
- $\beta_{ij}$ is the ij^th^ two factor interaction effect
- $\beta_{ii}$ is the ii^th^ quadratic effect
- $\beta_{iii}$ is the iii^th^ cubic effect
- $\gamma_{i}$ is the ith fixed block effect
- x is the input parameter setting of the ith, jth or kth parameter
- $s_{i}$ is the ith block setting
- p is the number of investigated input parameters
- b is the number of fixed block effects
- ε is the residual error term, assuming $\varepsilon \;\sim \;N\left(0,\sigma_{within\;batch}^{2}\right)$

Input parameters were selected through a pruned forward selection process as implemented in JMP Pro^®^, with the Akaike Information Criterion corrected (AICc) for small sample size serving as the cut-off criterion [40] (pp. 624–625). Notably, the hierarchy principle was not utilized during testing. The pruned forward algorithm computes parameter estimates using a combination of forward and backward steps. The maximum number of steps in the algorithm is five times the number of input parameters. The chosen model is the one that provides the best solution according to the AICc [40] (pp. 318–319).

The quality of the OLS model was evaluated based on multiple criteria. For the RMSE, it was tested if the value falls within the known range of variability for the corresponding output parameter (CQA1: 0.20–0.30 units, CQA2: 0.15–0.25 units, CQA3: 0.40–0.60 units). The difference between the adjusted R^2^ and the predicted R^2^ (i.e., one-point cross validation) was calculated. A difference of less than 0.3 was deemed acceptable for this evaluation. It should be highlighted at this point, that the R^2^ alone is not an appropriate attribute to assess model quality. The R^2^ expresses only the proportion of variance the model explains from the total variance in the data. In fact, a model can have a good quality with a R^2^ equal to zero. This is the case when all investigated PPs have no impact on the output parameter.

Moreover, the residual distribution was investigated. Assumptions for OLS fitting are normally distributed residuals and homoscedasticity (i.e., constant variance). Heteroscedasticity can occur in the LDoE, especially across different blocks, for instance, when the analytical method is optimized and thus the method precision changes. This does not impact the estimated coefficients since mean and variance are independent from each other for normally distributed data [41]. However, the heteroscedasticity of the data can bias the estimated coefficient standard errors, the model uncertainty intervals and can bias the model selection process. Thus, diagnostic processes for the models involve a visual examination of residual normality (e.g., Residual Normal Quantile Plot) and the dispersion (e.g., Residual by Predicted Plot). For the detection of outliers, studentized residuals were considered.

In addition, the model’s predictive capacity was assessed using Prediction-versus-measured (PvM) plots. It was investigated whether at least 90% of all experiments fall within the 90% PI of the corresponding model.

#### 2.4.2. Linear Mixed Models

Initially, potential blocking effects were modeled as a fixed effect. If at least one block was significant and the total number of blocks was five or larger, all blocking effects were included in the model and transformed to random effects using a linear mixed model. Changing Equation (1) to a linear mixed model results in the following equation:

(2) $$Y=\beta_{0}+\sum_{i=1}^{p}\beta_{i}x_{i}+\sum_{i=1}^{p-1}\sum_{j=i+1}^{p}\beta_{ij}x_{i}x_{j}+\sum_{i=1}^{p}\beta_{ii}{x_{i}}^{2}+\sum_{i=1}^{p}\beta_{ii}x_{i}^{3}+u+\varepsilon$$

where:

- u was the random block term, assuming $u\sim N\left(0,\sigma_{betweenbatch}^{2}\right)$.

The equation shows that the fixed block effect term $\sum_{i=1}^{b-1}\gamma_{i}s_{i}$ was replaced by the random term u. This term represents the random batch-to-batch variability, assuming a normal distribution with mean zero and variance $\sigma_{u}^{2}$.

**Figure 2:**
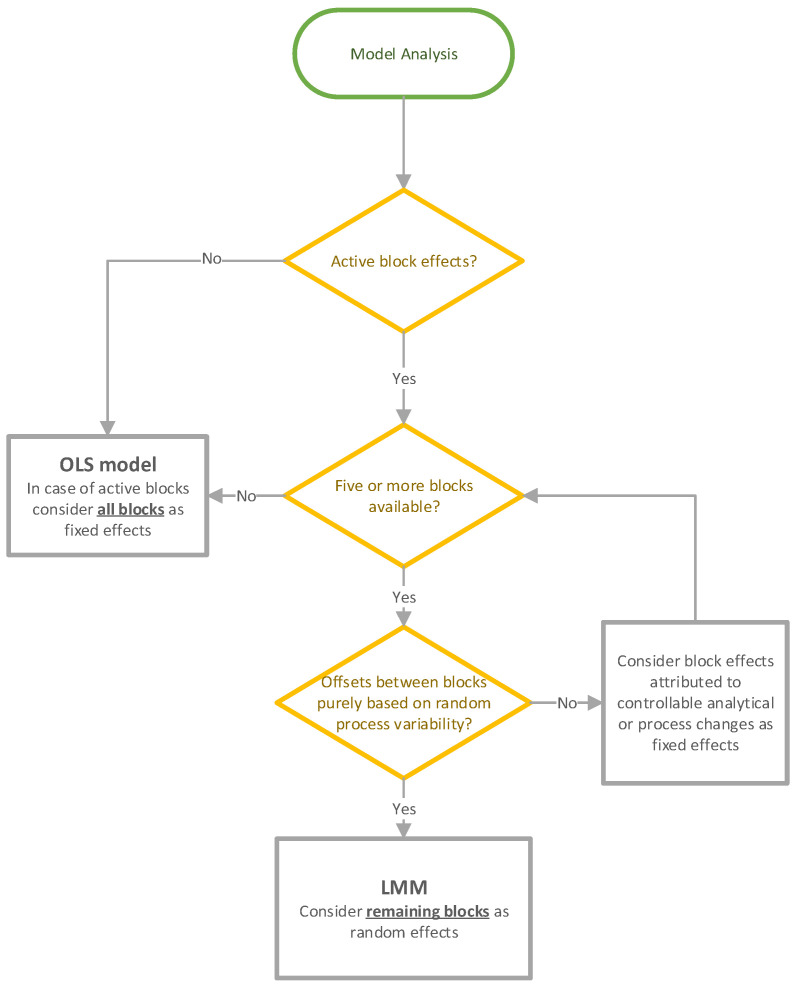
Workflow during the model analysis phase for OLS and LMM decision.

The threshold for the $\sigma_{between\;batch}^{2}$ estimation was set to five blocks to ensure a reliable estimation as pointed out by Piepho et al. [42]. In cases of active block effects but less than five blocks, blocks were treated as fixed effects. Figure 2 gives a detailed overview about the model analysis workflow.

The models were developed using restricted maximum likelihood (REML) and restricted variance component estimation towards positive values [40] (pp. 165–167). Only block effects that could be attributed to controllable analytical or process changes within development were still treated as fixed effects.

In addition to the previously performed OLS model evaluation, the assumption of normally distributed random blocks and residuals were analyzed via QQ-plots.

#### 2.4.3. Software

The design was evaluated and the statistical analysis was performed in JMP Pro version 18.0.1.

### 2.5. Process Optimization Analysis

The optimization of a bioprocess can be complex since multiple CQAs need to be fulfilled. For example, the titer needs to be maximized while the amount of aggregates needs be minimized. For the overall optimization, a so-called desirability function unifying the multiple goals was created. Its values range between 0 and 1. A desirability of 1 means that all optimization criteria were completely fulfilled, while a desirability of 0 indicates that the criteria were not at all met [43] (pp. 61–67).

### 2.6. Process Characterization Study

A process characterization study (PCS) is a comprehensive, systematic analysis conducted to gain a deep understanding of a specific process. It involves identifying and investigating the impact of pCPPs on CQAs. The goal is to establish a robust, reliable and efficient process that consistently produces the desired output under varying conditions. To achieve this, PARs are established. Furthermore, a criticality assessment is conducted to categorize PPs into highly potentially critical process parameters (hpCPPs), pCPPs and non-CPPs.

#### 2.6.1. Acceptance Limits

Acceptance limits (AL) for CQAs in a PCS are calculated based on process variability. Due to the higher number of experiments a DoE model provides a more accurate estimation of process variability than a limited number of replicates at process target conditions. Consequently, AL are calculated in this paper based on the model prediction at process target condition plus-minus three-times the model’s standard deviation, represented by the RMSE. However, due to the limited number of experiments in the initial phase of the LDoE, a four SD approach was adopted. This is because the model PI already has a three SD range and even minor effects would lead to an intersection between model PI and AL.

In the subsequent stages of the LDoE, data collected over a longer duration and from various batches become available. Typically, experiments from different WPs are grouped into blocks. There can be offsets between datasets from different blocks. These offsets can be accounted for as fixed effects or, in situations where the offsets represent process variability, they can be considered as random effects. LMMs are employed to account for variability within and between batches in the data. The sum of σ_within batch_ and σ_between batch_ was used for the target range calculation. To ensure a reliable estimation of the random effect, the threshold for the $\sigma_{between\;batch}^{2}$ estimation was set to five blocks as pointed out by Piepho et al. [42]. In cases where data from less than five blocks where available, blocks were considered as fixed effects and thus AL were calculated purely on RMSE which equals σ_within batch._

The calculation for sigma total ($\sigma_{T}$) is as follows:

(3) $$\sigma_{T}=\sqrt[{2}]{\sigma_{within\;batch}^{2}+\sigma_{between\;batch}^{2}}.$$

If $\bar{y}$ represents the model prediction at process target condition, the acceptance criteria are calculated as:

(4) $$\bar{y}\pm 3*\sigma_{T}.$$

#### 2.6.2. Definition Normal Operation Ranges

Typically, PPs investigated in a PCS can only be controlled in manufacturing within a certain range. This range is called the normal operating range (NOR) and describes a region around the process target operating conditions that contain common operational variability (variability that cannot always be controlled) [44].

#### 2.6.3. Definition Proven Acceptable Ranges

The PAR evaluation is conducted based on regulatory guidelines and recommendations [44]. The definition of the PAR in ICH Q8 R2 is followed [10]:

“A characterized range of a process parameter for which operation within this range, while keeping other parameters constant, will result in producing a material meeting relevant quality criteria.”

**Figure 3:**
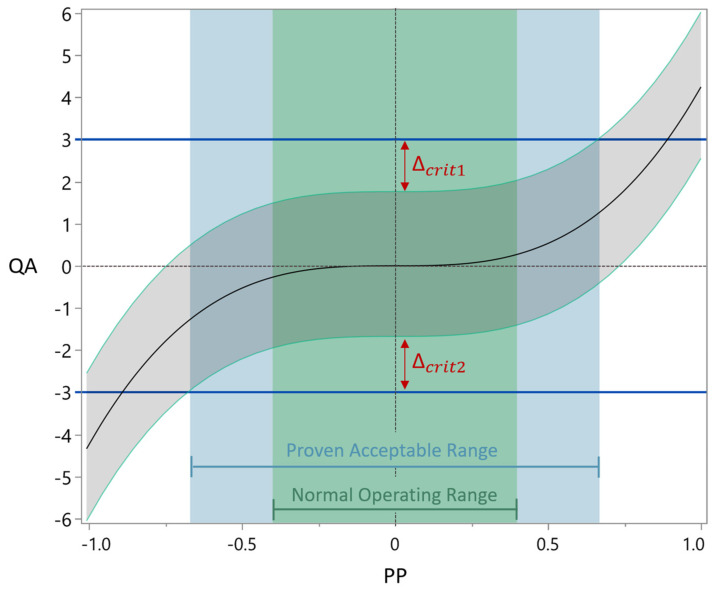
General illustration of a cubic QA model against a PP. The model mean prediction as black solid line, the 90% PI as grey shaded area, upper and lower AC as blue solid lines, the critical effects sizes as red arrows, as back dashed line the model prediction at process target conditions, the blue shaded area as PAR and the green shaded area as NOR.

The evaluation of PARs was performed model based. The range for the PPs is evaluated while keeping the other PPs at target operating condition. In cases where the 90% prediction interval (PI) of the model does not exceed the target range, the entire investigated parameter range can be designated as PAR. However, if the interval intersects with a target range, the PAR needs to be reduced to meet the target range. The calculated PAR for one output parameter (i.e., CQA) is then equivalent to the intersection of the 90% PI with the target range (Figure 3).

The lower limit of the overall PAR was calculated as the maximum value across each CQA-specific lower limit PAR, while the upper limit of the overall PAR was calculated as the minimum value across each CQA-specific upper limit PAR. This process was repeated for each input parameter. The rounding of the PARs was performed conservatively, the upper PAR was rounded down and the lower PAR was rounded up.

Within the PCS, it is essential to demonstrate that, at least within the NOR, any variation in the process at target operating conditions will result in PI and QA outcomes falling within the TRs. This implies that the PARs must be at least as broad as the NOR to demonstrate a robust process.

#### 2.6.4. Process Parameter Criticality Assessment

In order to identify pCPPs which can lead to PARs smaller than NORs in the PCS a preliminary PCS was conducted after each WP. A preliminary proven acceptable range (pPAR) was calculated and compared to the NOR of each PP. The upper respectively lower PAR–NOR ratio can be calculated by using the equation:

(5) $${ratio}_{PAR/NOR,p}=\frac{\left|x_{PAR,p}-x_{Target,p}\right|}{\left|x_{NOR,p}-x_{Target,p}\right|},$$

where:

${ratio}_{PAR/NOR}$ is the upper respectively lower PAR–NOR ratio for PP p

$x_{PAR,p}$ is the upper respectively lower PAR for PP p

$x_{NOR,p}$ is the upper respectively lower NOR for PP p

$x_{Target,p}$ is the target setting for PP p.

A PAR–NOR ratio where the upper and the lower ratio are greater than or equal to 1 suggests that for a given PP, the calculated PAR is at least as broad as the NOR. Conversely, a PAR–NOR ratiowhere either the upper or the lower ratio is less than 1 identifies pCPPs. For these parameters, it cannot be assured that within the NOR, all CQAs will fall within their AL. Figure 3 is an example for a PAR–NOR ratio where both sides are greater than 1. If the PAR overlaps on one or both sides with the NOR, that ratio is equal to zero and in cases where the NOR is on one or both sides wider than the PAR, that ratio becomes smaller than 1. PPs where both sides, the upper and the lower PAR–NOR ratios, are smaller than 1 are classified as hpCPPs.

During the initial stages of the LDoE, PPs frequently have settings at their extreme values (i.e., their lowest or highest possible setting in the design space) after optimization. Since regression models should not be used for extrapolation beyond the investigated design space [29] (p. 479), one limit of the PAR would be equal to the PP target setting, making the difference in the numerator of the PAR–NOR ratio equation equal to zero. While it is true that a process at the edge of the design space may not lead to a successful PCS and thus such a PP can be potentially critical, the concept of the LDoE is a continues refinement of the design space. In cases where better process conditions can be found at the edge of a design space, augmentations towards these ‘directions’ are essential.

#### 2.6.5. Multivariate Acceptable Ranges

In contrast to the PAR, for the multivariate acceptable ranges (MAR) calculation involves simultaneous variation of all PPs with a NOR. This method allows to investigate if specific PP combination leads to intersections between the models PI and the AL. Unlike PARs, multiple MARs can potentially exist for the same PPs. For example, broader ranges for one PP might be feasible if the ranges for a second PP are kept minimal and vice versa. As the number of PPs increases, numerous interaction effects must be considered concurrently. These effects can accumulate, resulting in a reduced MAR volume and occasionally leading to impractically narrow PP ranges. The approach presented in [12,45], which only reports MARs that do not intersect between the model’s PI and the AL was adapted. In our modified version, initially it was uniformly sampled from all PPs within the investigated design space, repeating this process one million times. Subsequently, it was evaluated whether each sample’s PI falls within the AL. The outcomes can be visualized in a scatterplot matrix, where simulated points within the AL are marked in green and those outside the AL are marked in red. This visualization highlights critical interactions among multiple PPs and facilitates an interactive search for potential MARs. By iteratively reducing the PP ranges, the proportion of simulated data points falling within the AL was calculated. It was determined that this proportion must be at least 99%.

#### 2.6.6. Retrospective Power Analysis

Detecting a critical effect size (${∆}_{crit}$), which can lead to an intersection between AL and model PIs within the corresponding OR is crucial. The relative critical effect size can be inferred by calculating the absolute difference between the 90% PI of the model at process target conditions and the corresponding target ranges (refer to Figure 3), divided by the RMSE of the model.

The investigated design space, spanned by the PP ranges, is usually broader than the PP NOR. Since the critical coefficient size needs to be detected within the NOR and not within the complete investigated PP range, the assumed relative critical coefficient sizes need to be individually scaled for each model term using the equation:

(6) $$\beta_{crit\;scaled,i}=\frac{min\left\{\left|{∆}_{crit1,p}\right|,\left|{∆}_{crit2,p}\right|\right\}}{\max\left\{\left|x_{NOR_{upr},i}-x_{Target,i}\right|,\left|x_{NOR_{lwr},i}-x_{Target,i}\right|\right\}*\sigma_{within\;batch}},$$

where:

$\beta_{crit\;scaled,i}$ is the critical NOR-scaled relative coefficient size for model term i,

${∆}_{crit,p}$ is the absolute critical effect size for PP p,

$x_{NOR,i}$ is the upper respectively lower coded OR for model term i,

$x_{Target,i}$ is the coded process target value for model term i, and

$\sigma_{within}$ is the model RMSE.

At the design creation stage, the model, including the model PIs and RMSE, is typically unknown. Therefore, the critical effect size must be assumed. For this, effects within the OR equal to or greater than 1.5 times the model RMSE are deemed critical. This is equivalent to a relative coefficient size of 1.5 for main effects and two-factor interactions and 3 for quadratic effects. The coefficient size for quadratic terms is twice as high since the coded input parameter ranges are scaled between 0 and +1, instead of −1 and +1. In cases where the NOR is not known, the complete PP range was considered as NOR, which is the most conservative assumption. Instead, perfectly controlled PPs have no OR and were excluded from the retrospective power analysis.

It is crucial to validate the assumed relative critical effects size after model analysis. If the assumed relative critical effect size (e.g., 1.5) was larger than the real relative critical effect size (calculated with the model), the power analysis needs to be revised with a posterior power analysis to assess if the design was capable of detecting all critical effects. This last step was only applied for the last WP in this paper. However, it should be highlighted that this calculation can be applied after each LDoE analysis step.

### 2.7. Data

**Figure 4:**
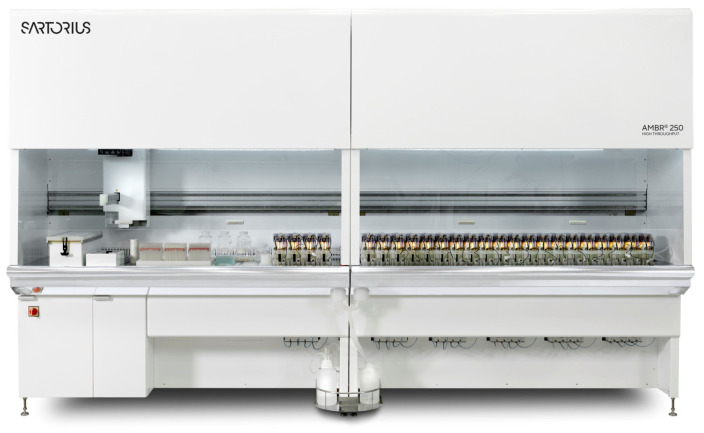
The Ambr^®^ 250 system is a high throughput, automated bioreactor system with 24 single-use 250 mL bioreactors. ©Sartorius AG, Image provided by courtesy of Sartorius AG.

Data from 97 experiments was generated in seven WPs during CMC development. For WP 1–4 DoE-based data and for WP 5–7 data from other experimental studies such as scale-down model studies were used. All experiments were performed in an Ambr^®^ 250 mL automated bioreactor system (Figure 4). The Sartorius Ambr^®^ 250 system is a state-of-the-art, high-throughput bioreactor platform designed for efficient process development. It features automated control of critical parameters such as pH and dissolved oxygen, enabling precise and reproducible conditions across multiple single-use bioreactors. Each WP can consist out of maximal 24 individual experiments (sometimes less than 24 experiments were available) and was considered as a separate block. The impact of nine different PPs on 11 CQAs was investigated. In this paper the focus is on three CQAs (CQA 1–3) to show the principles of the LDoE methodology.

All CQAs were mean-centered and PPs were coded between −1 and +1 previous to data analysis for data censoring reasons. The PP coding −1 represent the lowest and +1 represents the highest PP setting across all 97 experiments. The applied PP coding does not affect the underlying DoE scaling on the interval [−1, +1] usually applied on the input parameters to reduce correlations between the PPs and thus improve model selection [30] (p. 24). The scaling is applied individually for each LDoE analysis cycle. In other words, in the first three WPs PP3 was investigated in a coded range from −0.33 to +1, this range was scaled on the interval [−1, +1]. The same was done, after the range of PP3 was extended towards lower settings in WP4. However, here the coded range equals the scaled interval [−1, +1]. The difference between coded PPs and scaled PPs was applied to retain the information of the investigated PP ranges across different WPs despite data censoring. Table 1 gives a detailed overview of the investigated PP ranges across the different WPs.

## 3. Results

The results in this paper are organized in the subsequent sections WP specific. Each WP group contains detailed information about the used experimental design characteristics, process optimization and PP criticality assessments. The results of the previous WP group lead to decisions for the setup of the subsequent WP group. A more detailed explanation regarding the decisions can be found in the Section 4.

### 3.1. Work Package 1

#### 3.1.1. Design Analysis

An initial D-optimal design with 21 runs was created for PP 1–5, considering all main effects and two-factor interaction effects. Table 2 shows all PP ranges. Due to the limited number of experiments, quadratic effects were considered as secondary importance for three out of the five PPs via the ‘if-possible’ option in JMP^®^. This allows to identify at least strong quadratic effects for PP 1, 2 and 5 while keeping the importance on the main and two-factor interaction effects. Thus, this design can be understood as an initial screening design to identify the most important PPs. The design quality regarding the quadratic effects is improved in the first augmentation. Consequently, these three quadratic effects were not part of the design analysis in the first WP.

All model terms have a power 80% for an anticipated coefficient size of 1.5 or below and the Pearson correlation matrix shows only small correlations between the PPs. All design diagnostics including the design table can be found in Supplementary Materials (Supplementary SA).

#### 3.1.2. Model Analysis

**Figure 5:**
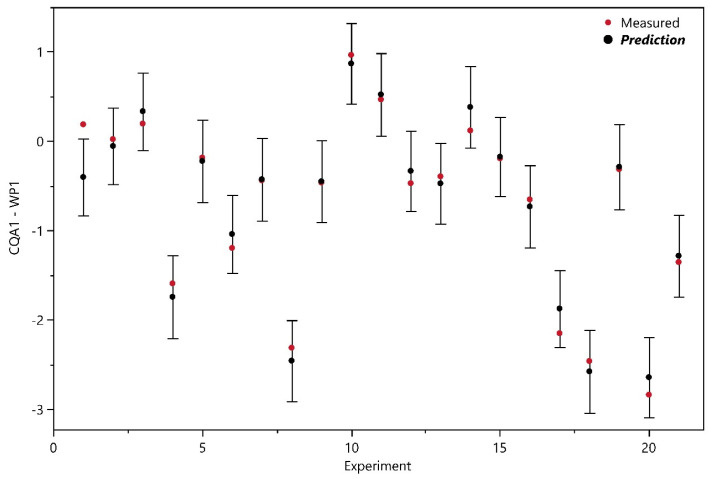
Prediction-versus-Measured Plot for CQA1. In black, model prediction with the 90% PIs and in red, the measured original data.

The models fulfill all quality criteria. Model quality parameters are summarized in Table 3 and a detailed analysis can be found in Supplementary Materials (Supplementary SA). The prediction-versus-measured plot for CQA1 in Figure 5 shows that except of experiment 1, all experiments fall within the 90% model PI and thus, the model can predict the data in more than 95% of the cases correct. Supplementary Materials (Supplementary SA) shows the prediction-versus-measured plots for all CQAs.

#### 3.1.3. Process Optimization Analysis

**Figure 6:**
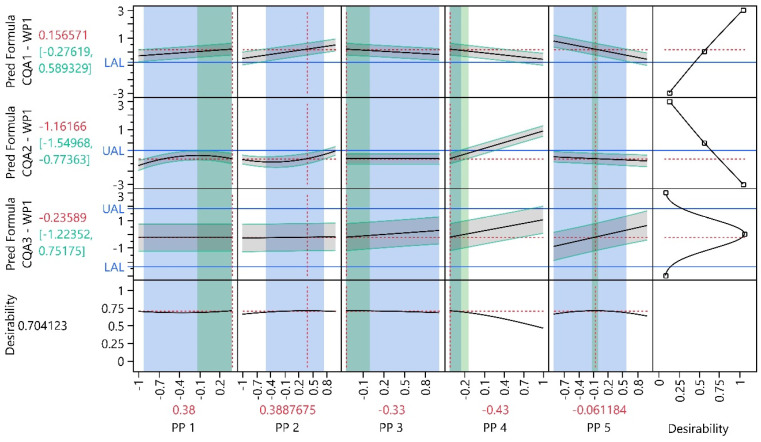
Prediction profiler of all CQA models at preliminary optimal process conditions. Each column represents a distinct input parameter, while each row corresponds to a different output parameter model. Black solid lines indicate the model mean prediction, green intervals and grey shaded areas indicate 90% PIs, horizontal blue solid lines indicate the UAL/LAL and vertical red dotted lines indicate PP settings. The green shaded background shows the NOR and the blue shaded background, the overall pPAR. The last column shows the desirable CQA outcomes (e.g., CQA1 should be maximized, CQA 2, minimized and CQA 3 should be kept as close as possible to the initial value of zero). The last row, called ‘Desirability,’ represents an individual optimization function for each CQA. The individual optimization functions for each CQA are summarized and visualized as a function of the PPs.

In the initial WP, an optimization was conducted to identify PP settings that yield to desirable CQA values. The objective was to maximize CQA1, minimize CQA2 and keep CQA3 as close to zero as possible. Figure 6 illustrates that improved PP settings were identified compared to the initial center point settings: CQA1 increased by approximately 0.9 units, CQA2 decreased by approximately 1.0 units and CQA3 is now 0.5 units closer to zero. The optimal settings for PP1, PP3 and PP4 are at their extreme setting (i.e., their lowest respectively highest possible setting). For example, higher settings for PP1 resulted in increased CQA1 values while simultaneously reducing CQA2 values (for PP1 settings greater than -approximately −0.1), with no observed impact on CQA3.

A detailed overview of the PP settings can be found in Supplementary Materials (Supplementary SB).

#### 3.1.4. Process Parameter Criticality Assessment

Figure 6 shows that PP4 has the highest impact on the CQAs, followed by PP5. The impact of PP1-3 within their investigated PP range is relatively minor. pPARs were calculated for all PPs and PAR–NOR ratios were calculated for all PP were NORs were available to assess PP criticality (Table 4). For PP5 a two-sided pPAR could be found which is wider than the NOR. For PP1 and PP3 at least a one-sided pPARs greater than the respective NOR could be found. Consequently, their NOR-PAR ratios are greater than 1. Figure 6 shows this as the blue shaded areas exceed the green shaded areas. PP2 has no NOR, since this PP can be perfectly controlled later in manufacturing. Only PP4 show a one-sided PAR–NOR ratioclearly smaller than 1. The reason for this is its high impact on CQA2. Thus, PP1, PP3 is classified as pCPP and PP4 as hpCPP.

### 3.2. Work Package 1–3

The evaluation of WP 1 pinpointed optimal PP settings for PP1, PP3 and PP4, located at the edge of the design space. This suggests that better CQA values could be obtained with increased PP1 settings and reduced PP4 settings, indicating a potential benefit in expanding the design space towards these areas. PP3’s overall influence was minor, leading to the decision to maintain its optimal settings within WP3, thereby conserving experiments. Furthermore, three additional PPs (PP5 to PP8) were incorporated into the LDoE. The best settings for PP6 are found in the design space, whereas PP7 and PP8 remains at their target settings of zero. Their criticality is assessed at the optimal settings of the other PPs.

#### 3.2.1. Design Analysis

Two D-optimal design augmentations with 38 experiments were performed on the initial LDoE from WP1 with 21 experiments, culminating in a total of 59 experiments. Table 5 highlights all PP modifications to the initial WP1 design.

The design analysis accounted for all main effects, two-factor interaction terms (excluding interactions between PP3 and PP2 with PP7–8) and quadratic terms for PPs with more than 2 levels. The PP2 and PP3 setting remained constant at the 0 and −0.33 level, respectively, with varying settings for PP7–8. In the optimization PP7 and PP8 were kept fixed on their target setting of zero. Therefore, no interaction effects between these PPs could occur and bias the analysis. Potential offsets between the three WPs were considered using the blocking factor ‘Work package’.

All model terms have a power 80% for an anticipated coefficient size of ≤1.5 respectively ≤3.0 (for quadratic terms) or below for a power of 80%. Except for the interaction term PP2*PP6 with 2.0 and the quadratic terms of PP4, PP7 and PP8 with slightly higher coefficients of approximately 3.13–3.25. These exceptions are due to the correlation structure between these PPs, and the design quality concerning these terms is enhanced in the subsequent augmentation. For process optimization purposes, the quadratic effects are borderline but considered acceptable. Like for PP7 and PP8, PP2 was kept fixed on the target setting of zero during the optimization.

Thus, only PP-terms which fulfill the design quality attributes are used for the process optimization analysis. All design diagnostics, including the design table, can be found in Supplementary Materials (Supplementary SC).

#### 3.2.2. Model Analysis

In the model analysis, the cubic effect from PP1 and the block effects from the WPs were considered as possible model terms. Following the model selection, no block effect was active, but a small cubic effect could be identified for CQA1. The models meet all quality criteria. Model quality parameters are summarized in Table 3 and a detailed analysis can be found in Supplementary Materials (Supplementary SC), where the data points are color-coded according to their WPs (WP1 in red, WP2 in green and WP3 in blue). Supplementary Materials (Supplementary SC) presents the prediction-versus-measured plots for all CQAs. More than 90% of all experiments fall within the 90% model PI, indicating the model’s predictive accuracy.

#### 3.2.3. Process Optimization Analysis

**Figure 7:**
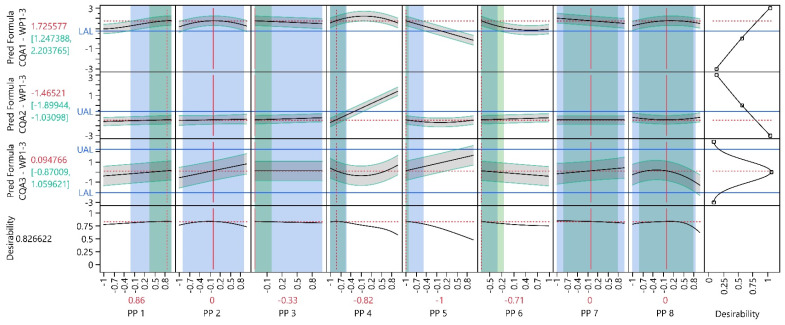
Prediction profiler of all CQA models at preliminary optimal process conditions. Each column represents a distinct input parameter, while each row corresponds to a different output parameter model. Black solid lines indicate the model mean prediction, green intervals and grey shaded areas indicate 90% PIs, horizontal blue solid lines indicate the UAL/LAL, vertical red dotted lines indicate PP settings and vertical red solid lines indicate fixed PP settings. The green shaded background shows the NOR and the blue shaded background, the overall pPAR. The last column shows the desirable CQA outcomes (e.g., CQA1 should be maximized, CQA 2, minimized and CQA 3 should be kept as close as possible to the initial value of zero). The last row, called ‘Desirability,’ represents an individual optimization function for each CQA. The individual optimization functions for each CQA are summarized and visualized as a function of the PPs.

In WP1–3, the optimization from WP1 was replicated with the same objectives. Figure 7 demonstrates that improved PP settings were identified compared to the settings in WP1. When comparing the CQA values from the initial optimal settings in WP1 with the new optimal settings in WP1–3, CQA1 increased by approximately 1.2 units, CQA2 decreased by approximately 0.5 units and CQA3 is now 0.1 units closer to zero. For a more accurate comparison, only the models obtained in WP1-3 were used. The optimal settings for PP3, PP5 and PP6 are at their extreme setting. A detailed overview is provided in Supplementary Materials (Supplementary SB).

#### 3.2.4. Process Parameter Criticality Assessment

Figure 7 indicates that PP4 continues to have the highest impact on the CQAs, followed by PP5 and PP6. The influence of the other PPs is relatively minor. pPARs were calculated for all PPs and PAR–NOR ratios were calculated for all PP were NORs were available to evaluate PP criticality (Table 6). For PP5, PP7 and PP8, a two-sided pPAR exceeding the NOR was identified. For PP1 and PP3, one-sided pPARs more than twice the size of the respective NOR were found. Shifting their target settings towards the midpoint of their pPAR would likely result in a pPAR encompassing their NOR. For PP4, almost both ratios and PP6 clearly both ratios are less than one. Therefore, PP1, PP3 and PP4 are classified as pCPPs, and PP6 as hpCPPs.

### 3.3. Work Package 1–4

WP4 was the final stage before the PPs needed to be fixed, preventing further changes. Subsequent WPs could only support the existing design space, for instance, by enhancing the power of PP effects through additional experiments.

Another PP (PP9) was identified by subject matter experts as a relevant parameter and was incorporated into the LDoE. Similar to PP7 and PP8 in WP1–3, PP9 remains at the target setting of zero during the optimization analysis. Its criticality is assessed at the optimal settings of the other PPs. Unlike WP1–3, all PPs, including PP2, PP3 and PP7-9, varied independently of each other. This allows for the identification of potential interaction effects of these PPs and the assessment of their criticality.

In WP1–3, preliminary optimal PP settings for PP1, PP3, PP4, PP5 and PP6 were located either at the edge or close to the edge of the design space. However, from a manufacturing process perspective, expanding the parameter range of PP1, PP4 and PP5 would lead to infeasible parameter settings. Therefore, only the parameter range for PP3 and PP6 was expanded.

The evaluation of WP1-3 repeatedly identified PP4 and PP6 as pCPP and hpCPP, respectively. While less critical settings for PP6 could be identified due to the design augmentation, this option was not applicable for PP4. Instead of further expanding the parameter range for PP4, the resolution was increased. Two additional levels (−0.71 and −0.14) were added to reveal potentially stable areas within the already observed parameter range.

#### 3.3.1. Design Analysis

A D-optimal design augmentation with 32 experiments was performed on the LDoE from WP1–3 with 59 experiments, resulting in a total of 81 experiments available for the evaluation of WP1–4. Table 7 highlights all PP modifications to the initial WP1–3 design.

The design analysis accounted for all main effects, two-factor interaction terms and quadratic terms. Potential offsets between the four WPs were considered using the blocking factor ‘Work package’ (Supplementary Materials (Supplementary SD)).

All model terms involving the new PP9 does not fulfill the requirement of an anticipated coefficient size of ≤1.5 respectively ≤3.0 (for quadratic terms) for a power of 80%. Similar observations could be made for terms including PP2 and PP3, which were kept constant within WP3 (PP 2*PP 8, PP 2*PP 6, PP 3, PP 2*PP 7, PP 3*PP 8, PP 3*PP 7 and PP 3*PP 6). To ensure that potentially overlooked effects (due to the lower power) cannot bias the optimization analysis, PP2 and PP3 were fixed on 0 and −0.33 respectively as in WP1-3.

#### 3.3.2. Model Analysis

**Figure 8:**
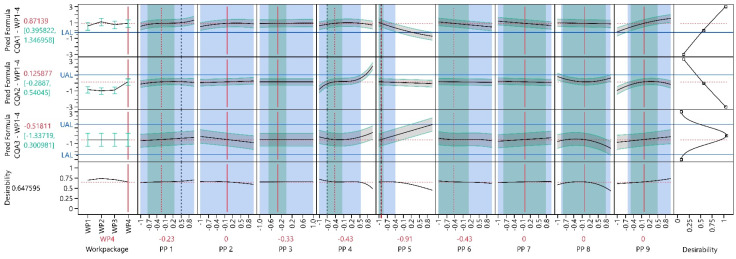
Prediction profiler of all CQA models at final optimal process conditions. Each column represents a distinct input parameter, while each row corresponds to a different output parameter model. Black solid lines as the model mean prediction, green intervals and grey shaded areas as 90% PIs, horizontal blue solid lines as the UAL respectively LAL, vertical grey dashed lines as preliminary optimal PP settings, vertical red dotted lines as final process target settings and vertical red solid lines as fixed PP settings. The green shaded background shows the NOR and the blue shaded background the overall pPAR. The last column shows the desirable CQA outcomes (e.g., CQA1 shall be maximized, CQA 2 minimized and CQA 3 shall be kept as close as possible to the initial value of zero). The last row, called ‘Desirability’ represents an individual optimization function for each CQA. The individual optimization functions for each CQA are summarized and visualized as a function of the PPs.

**Figure 9:**
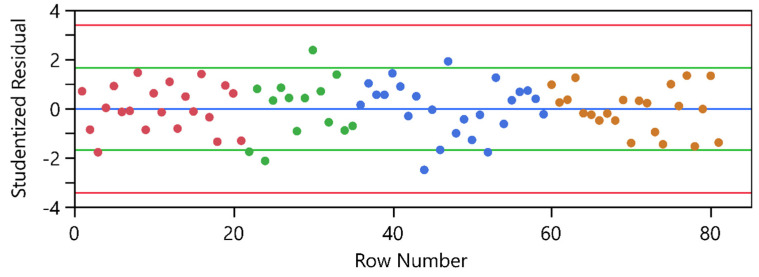
Studentized Residual plot for CQA2. Inner limits that appear in green on the plot are 95% individual t-distribution limits and outer limits that appear in red on the plot are 95% Bonferroni limits. Experiments from WP1 in red, WP2 in green, WP3 in blue and WP4 in orange.

While the observed block effects for CQA1 could be attributed to ‘normal’ batch-to-batch variability, this was not the case for CQA2, where the first 3 WPs clearly differentiate from WP4 (Figure 8 first column). This observation could be attributed to a change in the analytical method for CQA2. This constant offset between the two analytical methods was considered by the block effect. Moreover, the method change did not lead to a change in precision. This is supported by the studentized residual plot in Figure 9, where the dispersion of the orange WP4 group is comparable to the dispersion of the other three WPs.

As in WP1–3, a small cubic effect could be identified for PP1 on CQA1 and new also a small cubic effect for PP6 on CQA3. PP4 shows a strong cubic effect on CQA2.

The models meet all quality criteria. Model quality parameters are summarized in Table 3 and a detailed analysis can be found in Supplementary Materials (Supplementary SD) presents the prediction-versus-measured plots for all CQAs. More than 90% of all experiments fall within the 90% model PI, indicating the model’s predictive accuracy.

#### 3.3.3. Process Optimization Analysis

In WP1–4, the optimization objectives from WP1–3 were used. However, given that this was the final WP before PPs were fixed, an additional boundary condition was added. This condition necessitated that the final optimal PP settings maintain a minimum distance from the edge of the design space, equivalent to half the NOR. This ensures that a PAR that is at least as broad as the NOR can be found. This lead for instance to an increase of PP5 from −1.00 to −0.91. For the optimization the blocking parameter was set fixed on WP4.

The introduction of the new boundary condition, constrained the optimization algorithm’s flexibility in identifying the optimal PP settings, compared to WP1–3. As illustrated in Figure 8 and Supplementary Materials (Supplementary SB), the identified PP settings are slightly worse compared to the settings in WP1–3. When comparing the CQA values from the initial optimal settings in WP1–3 with the new optimal settings in WP1-4, CQA1 decreased by approximately 0.30 units, CQA2 increased by approximately 0.31 units and CQA3 is now 0.14 units further from zero. For a more precise comparison, only the models obtained in WP1–4 were used.

Following the algorithmic optimization, manual adjustments were made for PP1 and PP4. For PP1, only a minor impact on CQA1–3 was observed, leading to a decision to alter the settings from 0.50 (indicated by the vertical grey dashed line in Figure 9) to −0.23 (indicated by the vertical red dashed line in Figure 9). Slightly inferior absolute values for CQA2 were accepted in exchange for a broader PAR. Consequently, the settings for PP4 were adjusted from −0.71 to −0.43, falling within the stable region. These manual adjustments resulted in an increase in CQA1 by approximately 0.02 units, an increase in CQA2 by approximately 0.22 units and CQA3 is now 0.27 units further from zero. Overall, the desirability was reduced by the manual changes by approximately 0.03.

#### 3.3.4. Process Parameter Criticality Assessment

pPARs were calculated where NORs were available to evaluate PP criticality (Table 8). For all PPs, PAR–NOR ratios of one or greater were identified. By accepting slightly suboptimal PP settings in the optimization analysis, the criticality for PP1, PP3 and PP6 was significantly reduced. For PP4, a stable region was identified, leading to a substantial increase in the PAR–NOR ratio. While in WP1–4, PP-settings could be found that classified all investigated PP as non-pCPPs, the lower PAR–NOR ratio for PP5, PP6 and PP8 are borderline.

### 3.4. Work Package 1–7

WPs 5, 6 and 7 were not conducted based on DoEs. Instead, data from other experimental studies such as scale-down model studies, were incorporated into the LDoE to enhance model predictivity further. These additional experiments were performed using solely the final optimal target settings from WP1–4. A total of 15 experiments from three WPs were added to the existing LDoE, resulting in a comprehensive set of 97 experiments. At this stage, no design analysis was performed of the LDoE. Instead, a retrospective power analysis was conducted after the model analysis to ensure that the final design was capable to identify all critical effects.

#### 3.4.1. Model Analysis

Active block effects were identified for all three CQAs. The block effects for CQA1 and CQA3 were attributed to standard batch-to-batch variability. However, this was not the case for CQA2. A change in the analytical method between the first three WPs and the last four resulted in a constant offset, which was treated as a fixed effect called ‘Analytical Method’. The dispersion in the studentized residual plot was consistent between the two analytical methods, indicating no change in analytical precision. All models incorporated more than five blocks, accounting for random batch-to-batch variability. Consequently, the OLS models were transformed into LMMs.

The models meet all quality criteria before and after the transformation into LMMs. The models met all quality criteria, both before and after the transformation into LMMs. Model quality parameters are summarized in Table 3 and a detailed analysis is available in Supplementary Materials (Supplementary SE). Supplementary Materials (Supplementary SE) also present the prediction-versus-measured plots for the OLS and LMMs, respectively. Over 90% of all experiments fell within the 90% model PI, demonstrating the model’s predictivity.

#### 3.4.2. Retrospective Power Analysis

A retrospective power analysis was performed, considering the critical effect size. Table 9 shows that only one critical effect size per CQA was calculated because CQA1 and CQA2 have one-sided AL and the AL of CQA3 are symmetric around the target conditions. CQA2, with a signal-to-noise ratio of 2.03, represents the most conservative approach for calculating the relative critical effect sizes. A detailed overview is provided in Supplementary Materials (Supplementary SE), where the lowest power was observed for the quadratic term of PP6 at 88.2%. Therefore, the final LDoE design was capable of identifying all critical effects with a sufficiently high probability.

#### 3.4.3. Process Characterization Study

**Figure 10:**
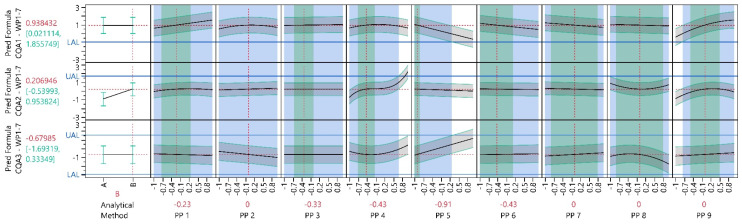
Prediction profiler of all CQA models at final optimal process conditions. Each column represents a distinct input parameter, while each row corresponds to a different output parameter model. Black solid lines as the model mean prediction, green intervals and grey shaded areas as 90% PIs, horizontal blue solid lines as the UAL respectively LAL, vertical grey dashed lines as preliminary optimal PP settings, vertical red dotted lines as final process target settings. The green shaded background shows the NOR and the blue shaded background the overall PAR.

For the PCS, the LDoE Models were used to calculate the final PARs. The models were set on the same PP settings as in WP1–4. The newly added block parameter was set to the value ‘B’, representing the new analytical method for CQA2. Figure 10 provides a visual representation of the final models and Table 10 provides a summary of the PARs for all investigated PPs. For all PPs PARs were found to be at least as wide as their NORs.

#### 3.4.4. Multivariate Acceptable Ranges

**Figure 11:**
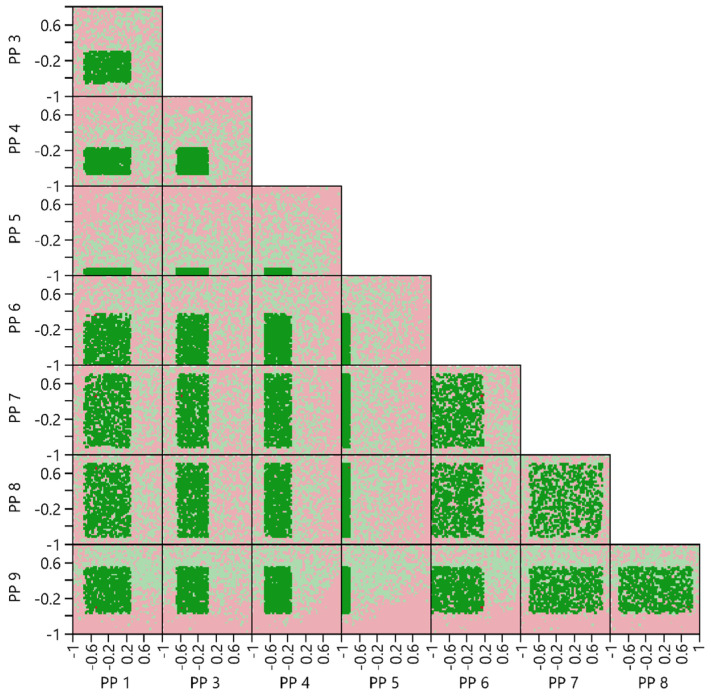
Design Space Simulation Results as a Scatterplot Matrix for MAR Calculation. This scatterplot matrix provides a two-dimensional projection of a nine-dimensional hypercube. Each quadrant displays the same simulated data points from different perspectives of the hypercube. The green points represent samples falling with their PIs in the AL of all CQAs, while red points represent samples where at least for one CQA an intersection between PI and AL occurred. Dark green and dark red points highlight the chosen MAR.

A MAR calculation was performed for PP1 and PP3–9. This involved conducting one million simulations across the entire design space, while maintaining PP2 at its target value of zero and setting the block parameter ‘Analytical Method’ to ‘B’. The outcome of this simulation is presented in a scatterplot matrix in Figure 11. This plot provides a two-dimensional representation of the eight-dimensional design space. Each quadrant displays all one million simulated data points, plotted against two different PPs.

For instance, the quadrant illustrating PP5 versus PP9 reveals that a high setting for PP5 combined with a low setting for PP9 results in an increased number of events outside the AL. Conversely, low settings for PP5 in combination with high settings for PP9 enhance the number of data points falling within the AL.

By choosing the NOR for each PP as the MAR, approximately 99.6% of all simulated data points fall within the AL.

## 4. Discussion

In this paper, the advantages of conducting and coordinating experiments systematically throughout the development process of a biopharmaceutical product is illustrated. The LDoE method was successful in pinpointing optimal PP settings within the design space for a range of optimization objectives. Furthermore, the methodology proved its adaptability in situations where optimal settings were found at the periphery of the design space. PP ranges were progressively expanded, enabling the identification of superior process settings. Additional PPs, which became relevant for the SMEs during process development, were seamlessly integrated into the LDoE. Moreover, the approach can be combined with existing approaches like the IPM [17,18] to integrate several unit operations of the bioprocess together, allowing the creation of a holistic process model.

Beginning with preliminary screening experiments in WP1, it was possible to identify hpCPP and pCPPs early in the process development using the PAR-to-NOR ratio. Unlike the proposed approach from Hakemeyer et al. [24], where an arbitrary threshold was introduced to classify the criticality of PP effects, which introduced an arbitrary threshold to classify the criticality of PP effects, the pPAR was compared with the NOR to contextualize the criticality assessment within a PCS-relevant framework. Two strategies to manage CPPs were proposed. One approach is to broaden the design space to locate PP combination settings where the impact of these PPs is less critical. For some PPs, like PP4, the entire possible range was already examined and the criticality remained high. In such instances, increasing the resolution respectively the number of levels for this PP is recommended. Typically, DoEs are conducted with parameters on two or three levels, sufficient for investigating linear or quadratic effects in most cases. However, the LDoE explores a broader parameter range compared to DoEs without design augmentations. Therefore, within the larger design space, more complex effects may arise and their consideration leads to a better process understanding. This approach was demonstrated with 4 out of the 9 PPs, with the most significant impact and benefit observed for PP4. The introduction of additional PP levels and the consideration of the cubic effect identified a robust region within the design space. This discovery led to wider PARs and ultimately reduced the criticality for this parameter.

The importance of robust PP settings over optimal PPs in WP1-4 was also emphasized. While maximizing process performance and minimizing impurities is crucial, it is equally important to find process settings where these outcomes are reproducible, at least within the NOR of the PPs. This was achieved manually after the initial optimization process by the algorithm. However, this process could be automated by developing an algorithm that can incorporate both optimal and robust PP settings.

In addition, a retrospective validation of the quality of the LDoE was conducted, assessing its ability to identify all critical effects within the observed design space. As Zhang et al. [46] have noted, a retrospective power analysis is typically considered “conceptually flawed and analytically misleading” due to the potential discrepancies between sample standard deviation and effect sizes and their population counterparts, which can subsequently affect the calculated power. However, in the approach, the critical effect size is directly linked to the AL derived from the model’s RMSE and the model’s PI. A high sample standard deviation would result in a broad AL and correspondingly wide PIs, while a low sample standard deviation would lead to a narrow AL and correspondingly smaller model PIs. Consequently, the critical effect size would remain approximately constant, making a retrospective power analysis a sensible approach in this particular scenario.

Additionally, a more flexible approach for the MAR calculation in comparison to [12,45] was introduced. A design space covering the PP NORs was created, demonstrating that operating within this MAR leads to CQA results falling in more than 99% of all cases within the predefined AL. As stated in the ICH Q8 (R2), where the authors explicitly mentioned “Analysis of historical data can contribute to the establishment of a design space,” [10] (p. 26) all available process development data were utilized to achieve this.

The LDoE encapsulates the complete process knowledge, stored in a single file. The models were converted into HTML files, making the comprehensive process development model easily accessible for the SMEs. This proved extremely beneficial in the FMEA, where the models, along with the PAR-to-NOR ratio, could support the PP ranking in a data-driven manner or in instances where questions regarding the process arose. The LDoE can be viewed as an interactive library, storing all process development information about a project.

During the LDoE implementation, valuable insights were gained that could benefit the readers. The following points should be considered when planning an LDoE:

- It is recommended to compile a list of all PPs to be optimized within development and investigated in the PCS. It is more efficient to consider all PPs in the initial phase of the LDoE rather than adding them sequentially over the WPs. This was evident in the design analysis of WP1–4, where the power of the newly added PP9 and all combinations with this parameter were below 80%. In our case, a definitive screening design [47,48] would have been appropriate to investigate the impact of all nine PPs, including three center point experiments with only 21 experiments. From there, additional design augmentation can be performed to extend the design space.
- The LDoE approach results in broader PP ranges compared to standard DoEs, increasing the risk of an edge-of-failure. An edge of failure is a PP setting combination that reproducible leads to a loss of the experiment. It is recommended to carefully inspect combinations, especially when multiple PPs are at their highest or lowest setting. Alternatively, performing test experiments upfront with the most suspicious settings is recommended.
- It is also recommended to compile a list considering all CQAs. It is crucial that data from all relevant analytical output parameters are available across the complete LDoE.
- Plan for enough retains from the experiments. In cases where analytical experiments need to be repeated or a new CQA appears within development, experiments from previous WPs would otherwise need to be repeated, dramatically increasing the resources.
- Advising against keeping PP settings fixed within one WP to save experiments. The design analysis in WP1–4 shows that many terms, including PP2 or PP3, which were kept constant in WP3, do not meet the design quality criteria regarding statistical power in the following WPs.
- It is recommended to initiate the LDoE at a stage of process development where no fundamental parameters will be changed. This is typically not the case in the very early phase of process development. Fundamental parameters are, for instance, cell lines or the medium. These parameters lead to many possible interaction effects with other PPs. While these higher-order interaction effects can theoretically be considered in the LDoE, they lead to a dramatic increase in required experiments.
- It is not recommended to include OFAT experiments into the LDoE with only one replicate. While OFAT experiments can theoretically be modeled in the form of linear or quadratic effects, without any replicate, they have a very high leverage. This leads to worse model diagnostics, for instance, the one-point cross-validation (predicted R^2^) loses its informative value and potential underestimation of the variance can appear.

## 5. Conclusions

The LDoE approach demonstrates substantial advantages in aligning individual WPs within the process development of biopharmaceuticals. By consolidating process knowledge into a single, accessible model, the LDoE method significantly accelerates bioprocess development. This holistic approach allows for DoEs that consider the entire development process, thereby reducing the number of experiments required, particularly in the PCS phase.

The implementation of the LDoE approach not only enhances the efficiency of process development but also contributes positively to eco-design criteria. This is achieved through the reduction of consumables, water and raw materials, aligning with sustainable development goals. The iterative nature of the LDoE allows for continuous refinement and optimization, ensuring robust and reliable process settings that meet the CQA ALs.

Future applications of the LDoE approach will explore the integration of initial definitive screening designs with augmented optimal designs. This combination aims to further improve the quality and efficiency of the LDoE methodology, providing a more comprehensive and adaptable framework for bioprocess development. By leveraging these advanced design strategies, the LDoE approach can continue to support the rapid and sustainable development of biopharmaceutical products, ultimately enhancing the overall productivity and environmental footprint of the industry.

## Figures and Tables

**Table 1:** Overview of the investigated coded process parameter ranges across different working packages. Cells containing only one value belong to process parameters kept at a constant value within the corresponding work packages.

Process Parameter	WP1 21 Experiments	WP1-3 59 Experiments	WP1-5 91 Experiments	WP1-7 97 Experiments
PP1	[−1.00, +0.38]	[1.00, +1.00]	[1.00, +1.00]	[1.00, +1.00]
PP2	[−1.00, +1.00]	[−1.00, +1.00]	[1.00, +1.00]	[1.00, +1.00]
PP3	[−0.33, +1.00]	[−0.33, +1.00]	[1.00, +1.00]	[1.00, +1.00]
PP4	[−0.43, +1.00]	[−1.00, +1.00]	[1.00, +1.00]	[1.00, +1.00]
PP5	[−0.91, +1.00]	[−1.00, +1.00]	[1.00, +1.00]	[1.00, +1.00]
PP6	+0.14	[−0.71, +1.00]	[1.00, +1.00]	[1.00, +1.00]
PP7	0	[−1.00, +1.00]	[1.00, +1.00]	[1.00, +1.00]
PP8	0	[−1.00, +1.00]	[1.00, +1.00]	[1.00, +1.00]
PP9	0	0	[1.00, +1.00]	[1.00, +1.00]

**Table 2:** Overview of investigated process parameters and their investigated levels in WP 1.

Process Parameter	Lower Limit	Upper Limit	Number of Levels
PP1	−1.00	+0.38	4
PP2	−1.00	+1.00	3
PP3	−0.33	+1.00	2
PP4	−0.43	+1.00	2
PP5	−0.91	+1.00	2

**Table 3:** Summary Table—Model quality parameters.

Model—WP	Model Type	n	RMSE	R^2^	R^2^ Adjusted	R^2^ Predicted	Heterosk.	Normally Distributed Residuals	Number of Experiments outside the 90% PI
CQA1—WP1	OLS	21	0.222	0.968	0.955	0.941	no	yes	1
CQA2—WP1	OLS	21	0.161	0.985	0.977	0.959	no	yes	0
CQA3—WP1	OLS	21	0.525	0.820	0.760	0.622	no	yes	0
CQA1—WP1–3	OLS	59	0.245	0.954	0.936	0.913	no	yes	2
CQA2—WP1–3	OLS	59	0.225	0.972	0.964	0.954	no	yes	1
CQA3—WP1–3	OLS	59	0.539	0.801	0.731	0.577	no	yes	2
CQA1—WP1–4	OLS	81	0.267	0.945	0.925	0.897	no	yes	2
CQA2—WP1–4	OLS	81	0.231	0.967	0.954	0.918	no	yes	3
CQA3—WP1–4	OLS	81	0.478	0.815	0.735	0.579	no	yes	2
CQA1—WP1–7	OLS	97	0.262	0.946	0.931	0.910	no	yes	2
CQA2—WP1–7	OLS	97	0.229	0.962	0.947	0.912	no	yes	5
CQA3—WP1–7	OLS	97	0.445	0.866	0.802	0.662	no	yes	1
CQA1—WP1–7	LMM	97	0.262	0.946	0.936	NA	no	yes	1
CQA2—WP1–7	LMM	97	0.229	0.962	0.951	NA	no	yes	2
CQA3—WP1–7	LMM	97	0.444	0.866	0.816	NA	no	yes	4

**Table 4:** Summary Table for PAR–NOR Ratios. pCPPs in light grey and hpCPPs with both PAR–NOR ratios smaller than those in dark grey.

Process Parameter	Target	NOR Lower	NOR Upper	pPAR Lower	pPAR Upper	Lower PAR–NOR Ratio	Upper PAR–NOR Ratio	CQA Limiting Lower pPAR	CQA Limiting Upper pPAR
PP1	0.38	−0.12	0.88	−0.92	0.38	2.60	0.00	CQA1	NA
PP2	0.39	NA	NA	−0.5	0.76	NA	NA	CQA1	CQA2
PP3	−0.33	−0.67	0.01	−0.33	1	0.00	3.91	NA	NA
PP4	−0.43	−0.72	−0.14	−0.43	-0.25	0.00	0.62	NA	CQA2
PP5	−0.06	−0.15	0.03	−0.91	0.58	9.44	7.11	NA	CQA1

**Table 5:** Overview of investigated process parameters and their investigated levels in WP 1–3. Grey cells highlight changes to the previous design space.

Process Parameter	Lower Limit	Upper Limit	Number of Levels
PP1	−1.00	+1.00	5
PP2	−1.00	+1.00	3
PP3	−0.33	+1.00	2
PP4	−1.00	+1.00	3
PP5	−1.00	+1.00	3
PP6	−0.71	+1.00	3
PP7	−1.00	+1.00	3
PP8	−1.00	+1.00	3

**Table 6:** Summary Table for PAR–NOR Ratios. pCPPs in light grey and hpCPPs with both PAR–NOR ratios smaller than those in dark grey.

Process Parameter	Target	NOR Lower	NOR Upper	pPAR Lower	pPAR Upper	Lower PAR–NOR Ratio	Upper PAR–NOR Ratio	CQA Limiting Lower pPAR	CQA Limiting Upper pPAR
PP1	0.86	0.36	1.36	−0.21	1.00	2.14	0.28	CQA1	NA
PP2	0.00	NA	NA	−0.82	0.90	NA	NA	CQA1	CQA1
PP3	−0.33	−0.67	0.01	−0.33	1.00	0.00	3.91	NA	NA
PP4	−0.82	−1.11	−0.53	−1.00	−0.52	0.62	1.03	NA	CQA2
PP5	−1.00	−1.09	−0.91	−1.00	−0.47	0.00	5.89	NA	CQA1
PP6	−0.71	−1.28	−0.14	−0.71	−0.30	0.00	0.72	NA	CQA1
PP7	0.00	−0.80	0.80	−1.00	1.00	1.25	1.25	NA	NA
PP8	0.00	−0.80	0.80	−1.00	0.86	1.25	1.08	CQA3	NA

**Table 7:** Overview of investigated process parameters and their investigated levels in WP 1–4. Grey cells highlight changes to the previous design space.

Process Parameter	Lower Limit	Upper Limit	Number of Levels
PP1	−1.00	+1.00	5
PP2	−1.00	+1.00	3
PP3	−1.00	+1.00	4
PP4	−1.00	+1.00	5
PP5	−1.00	+1.00	3
PP6	−1.00	+1.00	4
PP7	−1.00	+1.00	3
PP8	−1.00	+1.00	3
PP9	−1.00	+1.00	3

**Table 8:** Summary Table for PAR–NOR ratios.

Process Parameter	Target	NOR Lower	NOR Upper	pPAR Lower	pPAR Upper	Lower PAR–NOR Ratio	Upper PAR–NOR Ratio	CQA Limiting Lower pPAR	CQA Limiting Upper pPAR
PP1	−0.23	−0.73	0.27	−1.00	1.00	1.54	2.46	NA	NA
PP2	0.00	NA	NA	−1.00	1.00	NA	NA	NA	NA
PP3	−0.33	−0.67	0.01	−1.00	1.00	1.97	3.91	NA	NA
PP4	−0.43	−0.72	−0.14	−1.00	0.56	1.97	3.41	NA	CQA2
PP5	−0.91	−1.00	−0.82	−1.00	−0.38	1.00	5.89	NA	CQA1
PP6	−0.43	−1.00	0.14	−1.00	1.00	1.00	2.51	NA	NA
PP7	0.00	−0.80	0.80	−1.00	1.00	1.25	1.25	NA	NA
PP8	0.00	−0.80	0.80	−0.86	0.98	1.08	1.23	CQA2	CQA2
PP9	0.00	−0.50	0.50	−0.59	1.00	1.18	2.00	CQA1	NA

**Table 9:** Overview critical effect sizes, rounded on two digits.

CQA	Acceptance Limit	90% PI	${∆}_{crit}$	$\sigma_{within\;batch}$	$\frac{{∆}_{crit}}{\sigma_{within\;batch}}$
CQA1	−0.98	0.02	1.00	0.48	2.09
CQA2	1.72	0.95	0.77	0.38	2.03
CQA3	1.60	0.33	1.27	0.57	2.22

**Table 10:** Summary Table for PAR–NOR ratios.

Process Parameter	Target	NOR Lower	NOR Upper	pPAR Lower	pPAR Upper	Lower PAR–NOR Ratio	Upper PAR–NOR Ratio	CQA Limiting Lower pPAR	CQA Limiting Upper pPAR
PP1	−0.23	−0.73	0.27	−1.00	1.00	1.54	2.46	NA	NA
PP2	0.00	NA	NA	−1.00	1.00	NA	NA	NA	NA
PP3	−0.33	−0.67	0.01	−1.00	1.00	1.97	3.91	NA	NA
PP4	−0.43	−0.72	−0.14	−1.00	0.68	1.97	3.83	NA	CQA2
PP5	−0.91	−1.00	−0.82	−1.00	0.26	1.00	13.00	NA	CQA1
PP6	−0.43	−1.00	0.14	−1.00	1.00	1.00	2.51	NA	NA
PP7	0.00	−0.80	0.80	−1.00	1.00	1.25	1.25	NA	NA
PP8	0.00	−0.80	0.80	−1.00	1.00	1.25	1.25	NA	NA
PP9	0.00	−0.50	0.50	−0.75	1.00	1.50	2.00	CQA1	NA

## Data Availability

The censored raw data presented in the study are included in the supplementary material (Supplementary_SF_RawData).

## Supplementary Materials

The following supporting information can be downloaded at: https://www.mdpi.com/article/10.3390/bioengineering11111089/s1, Supplementary_SA_WP1, Supplementary_SB, Supplementary_SC_WP1_3, Supplementary_SD_WP1_4, Supplementary_SE_WP1_7, Supplementary_SF_RawData, Supplementary_SG_JMP_Files.

## List of Abbreviations

**Abbrevation**	**Full Word**
AICc	Akaike information criterion corrected
AL	acceptance limits
CCD	central composite design
CMC	chemical manufacturing and control
CPP	critical process parameter
CQA	critical quality attribute
DoE	Design of Experiment
EMA	European Medicine Agency
FDA	Food and Drug Administration
FMEA	failure mode and effects analysis
hpCPP	highly potentially critical process parameter
IPM	integrated process model
LDoE	Life-Cycle-DoE
LMM	linear mixed effect model
MAR	multivariate acceptable ranges
NOR	normal operating range
OFAT	one factor at a time
OLS	ordinary least squares
PAR	proven acceptable range
PAT	process analytical technology
pCPP	potential critical process parameter
pCQA	potential critical quality attribute
PCS	process characterization study
PI	prediction interval
PP	process parameter
pPAR	preliminary proven acceptable range
PvM	prediction-versus-measured
QbD	Quality by Design
REML	restricted maximum likelihood
RMSE	root mean square error
SD	standard deviation
SME	subject matter expert
WP	work package
