# Supplementary material for: Lifecycle DoE—The Companion for a Holistic Development Process
Source: Bioengineering (Basel). 2024 Oct 30;11(11):1089. doi: 10.3390/bioengineering11111089 (PMC11591819; doi:10.3390/bioengineering11111089)
Supplement: Supplementary file 1 [file bioengineering-11-01089-s001.zip › Supplementary_SA_WP1.pdf]

Evaluate Design

Design

Run	PP 1	PP 2	PP 3	PP 4	PP 5
1	-0.53846	0	-0.33333	1	-0.90909
2	-0.53846	0	1	-0.42857	-0.90909
3	-0.53846	0	-0.33333	-0.42857	-0.90909
4	0.384615	-1	-0.33333	-0.42857	1
5	-0.07692	1	-0.33333	-0.42857	1
6	0.384615	-1	1	-0.42857	0.048485
7	-1	-1	1	-0.42857	-0.90909
8	-0.53846	-1	1	-0.42857	1
9	0.384615	1	1	-0.42857	1
10	0.384615	1	-0.33333	-0.42857	-0.90909
11	0.384615	1	1	1	-0.90909
12	0.384615	1	-0.33333	1	0.048485
13	-1	1	1	-0.42857	0.048485
14	-1	1	-0.33333	-0.42857	-0.90909
15	-1	0	1	1	-0.90909
16	-0.53846	1	1	1	1
17	-1	-1	-0.33333	1	0.048485
18	-1	-1	1	1	1
19	0.384615	-1	-0.33333	1	-0.90909
20	-0.07692	-1	-0.33333	1	1
21	-1	1	-0.33333	1	1

Design Evaluation

Power Analysis

Significance Level 0.05

Anticipated RMSE 1

Term	Anticipated Coefficient	Power
Intercept	0.85	0.801
PP 1	1.207	0.801
PP 2	1.047	0.8
PP 3	0.938	0.8
PP 4	1.061	0.8
PP 5	0.971	0.801
PP 1*PP 2	1.073	0.801
PP 1*PP 3	1.033	0.8
PP 1*PP 4	1.139	0.801
PP 1*PP 5	1.383	0.801
PP 2*PP 3	1.098	0.8
PP 2*PP 4	1.056	0.8
PP 2*PP 5	1.143	0.8
PP 3*PP 4	0.967	0.8
PP 3*PP 5	0.942	0.801
PP 4*PP 5	1.007	0.801

Design and Anticipated Responses

Anticipated Response	PP 1	PP 2	PP 3	PP 4	PP 5
-1.00939	-0.53846	0	-0.33333	1	-0.90909
-1.05091	-0.53846	0	1	-0.42857	-0.90909
1.576056	-0.53846	0	-0.33333	-0.42857	-0.90909
-1.8573	0.384615	-1	-0.33333	-0.42857	1
-0.62711	-0.07692	1	-0.33333	-0.42857	1
-1.29236	0.384615	-1	1	-0.42857	0.048485
0.259114	-1	-1	1	-0.42857	-0.90909
-2.02999	-0.53846	-1	1	-0.42857	1
6.489257	0.384615	1	1	-0.42857	1
-2.73933	0.384615	1	-0.33333	-0.42857	-0.90909
6.048721	0.384615	1	1	1	-0.90909
3.405709	0.384615	1	-0.33333	1	0.048485
-1.31868	-1	1	1	-0.42857	0.048485
-0.17762	-1	1	-0.33333	-0.42857	-0.90909
-1.09854	-1	0	1	1	-0.90909
9.14801	-0.53846	1	1	1	1
-1.24332	-1	-1	-0.33333	1	0.048485
-1.297	-1	-1	1	1	1
-2.04596	0.384615	-1	-0.33333	1	-0.90909
-0.56828	-0.07692	-1	-0.33333	1	1
-0.5936	-1	1	-0.33333	1	1

Color Map on Correlations

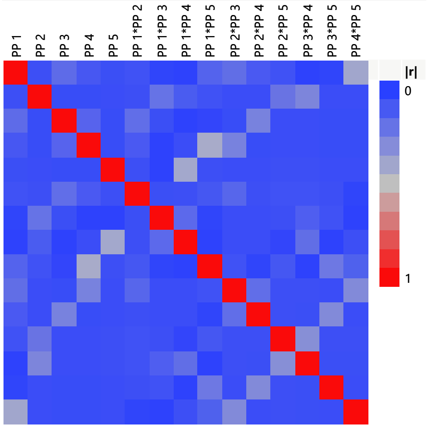

Output Options

Fit Group

Response CQA1 - WP1

Whole Model

Effect Summary

Source	Logworth	PValue
PP 5(-0.91,1)	9.043	0.00000
PP 2	6.359	0.00000
PP 2*PP 5	3.355	0.00044
PP 1(-1,0.38)	3.008	0.00098
PP 4(-0.43,1)	2.480	0.00331
PP 3*PP 4	2.461	0.00346

Actual by Predicted Plot

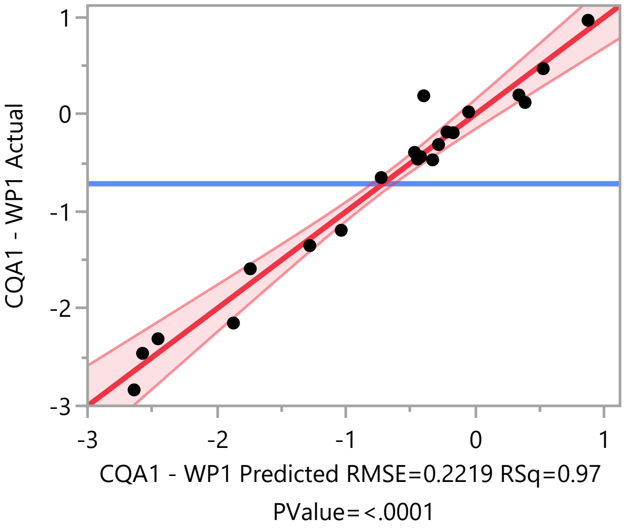

Summary of Fit

RSquare	0.968225
RSquare Adj	0.954607
Root Mean Square Error	0.221852
Mean of Response	-0.71528
Observations (or Sum Wgts)	21

Analysis of Variance

Source	DF	Sum of Squares	Mean Square	F Ratio
Model	6	20.996371	3.49940	71.0998
Error	14	0.689053	0.04922	**Prob > F**
C. Total	20	21.685424		<.0001*

Fit Group

Response CQA1 - WP1

Whole Model

Parameter Estimates

Term	Estimate	Std Error	t Ratio	Prob>|t|
Intercept	-0.731051	0.049899	-14.65	<.0001*
PP 1(-1,0.38)	0.2425973	0.058451	4.15	0.0010*
PP 2	0.525653	0.059656	8.81	<.0001*
PP 4(-0.43,1)	-0.173007	0.048962	-3.53	0.0033*
PP 5(-0.91,1)	-0.780671	0.054368	-14.36	<.0001*
PP 2*PP 5	0.3160463	0.069236	4.56	0.0004*
PP 3*PP 4	0.1945213	0.055396	3.51	0.0035*

Residual by Predicted Plot

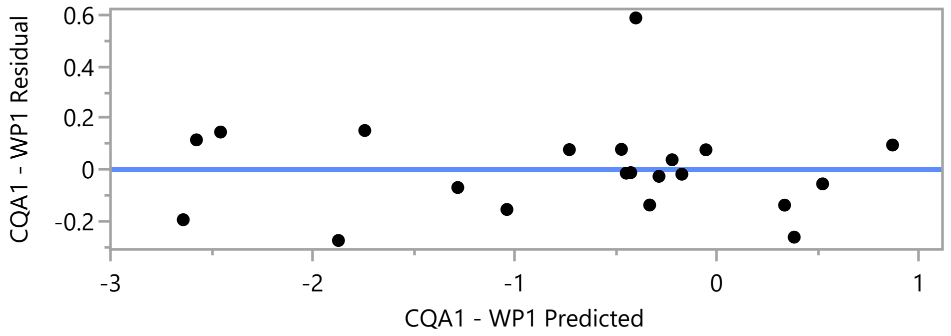

Prediction Profiler

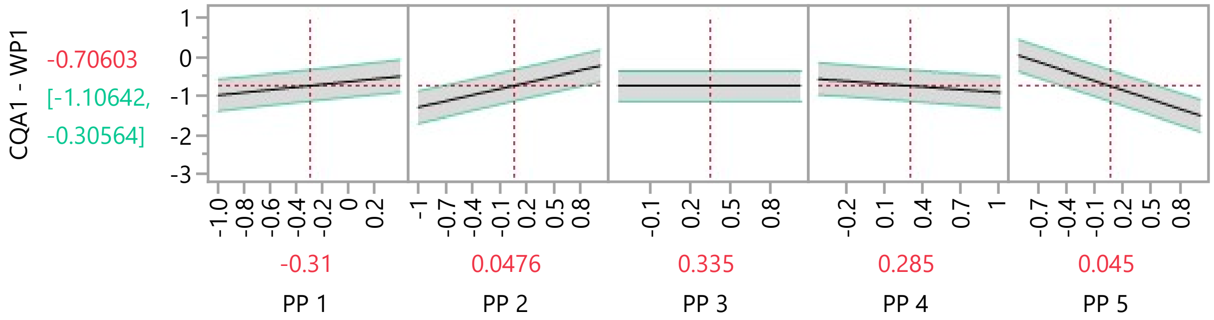

Press

Residual	SSE	RMSE	RSquare
Press	1.2844406232	0.24731324	0.9408
Ordinary	0.6890532935	0.22185151	0.9682

Response CQA2 - WP1

Fit Group

Response CQA2 - WP1

Whole Model

Effect Summary

Source	Logworth	PValue
PP 4(-0.43,1)	12.177	0.00000
PP 2	5.607	0.00000
PP 5(-0.91,1)	3.818	0.00015
PP 1(-1,0.38)	3.725	0.00019
PP 1*PP 1	2.941	0.00115
PP 2*PP 2	2.076	0.00839
PP 4*PP 5	2.045	0.00902

Actual by Predicted Plot

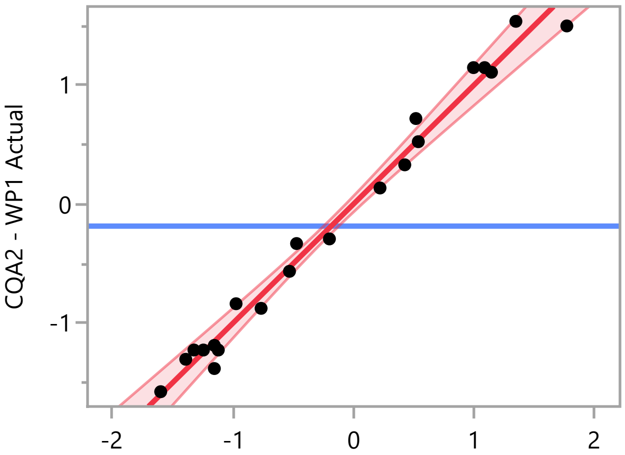

CQA2 - WP1 Predicted RMSE=0.1608
RSq=0.98513 PValue=<.0001

Summary of Fit

RSquare	0.985129
RSquare Adj	0.977122
Root Mean Square Error	0.16083
Mean of Response	-0.18413
Observations (or Sum Wgts)	21

Analysis of Variance

Source	DF	Sum of Squares	Mean Square	F Ratio
Model	7	22.276338	3.18233	123.0298
Error	13	0.336263	0.02587	**Prob > F**
C. Total	20	22.612601		<.0001*

Fit Group

Response CQA2 - WP1

Whole Model

Parameter Estimates

Term	Estimate	Std Error	t Ratio	Prob>|t|	Uncoded Estimate
Intercept	-0.184167	0.097446	-1.89	0.0813	-0.54293
PP 1(-1,0.38)	0.2530083	0.049172	5.15	0.0002*	-0.219185
PP 2	0.3124377	0.039417	7.93	<.0001*	0.3124377
PP 4(-0.43,1)	0.9845141	0.035815	27.49	<.0001*	1.3859836
PP 5(-0.91,1)	-0.28398	0.053902	-5.27	0.0002*	-0.240103
PP 1*PP 1	-0.449886	0.108464	-4.15	0.0011*	-0.944941
PP 2*PP 2	0.4374444	0.140959	3.10	0.0084*	0.4374444
PP 4*PP 5	-0.137185	0.044743	-3.07	0.0090*	-0.200908

Residual by Predicted Plot

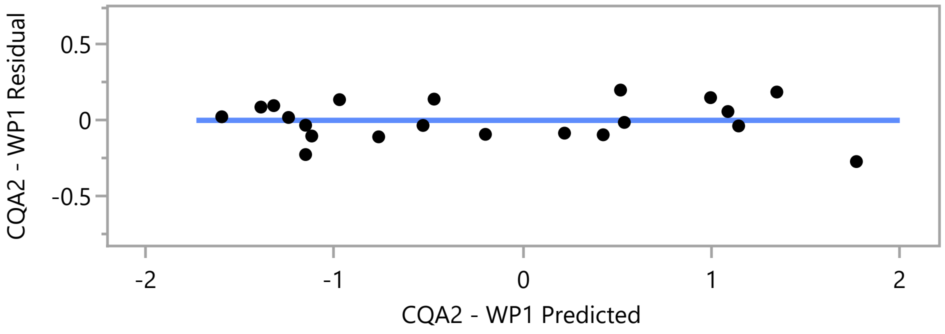

Prediction Profiler

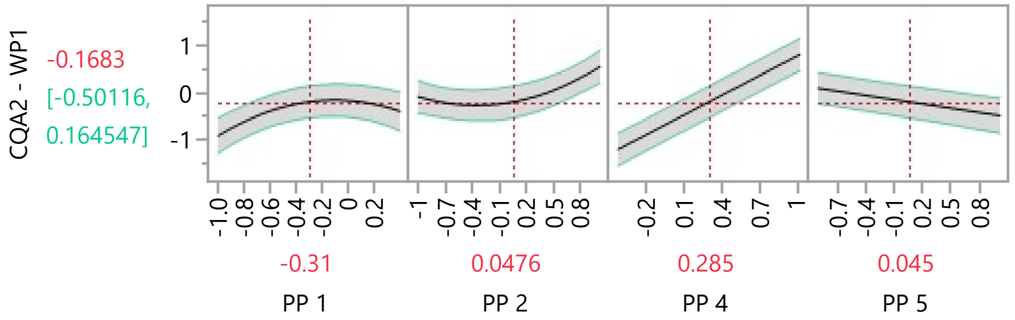

Press

Residual	SSE	RMSE	RSquare
Press	0.9332193927	0.21080564	0.9587
Ordinary	0.3362627501	0.16083024	0.9851

Fit Group

Response CQA3 - WP1

Actual by Predicted Plot

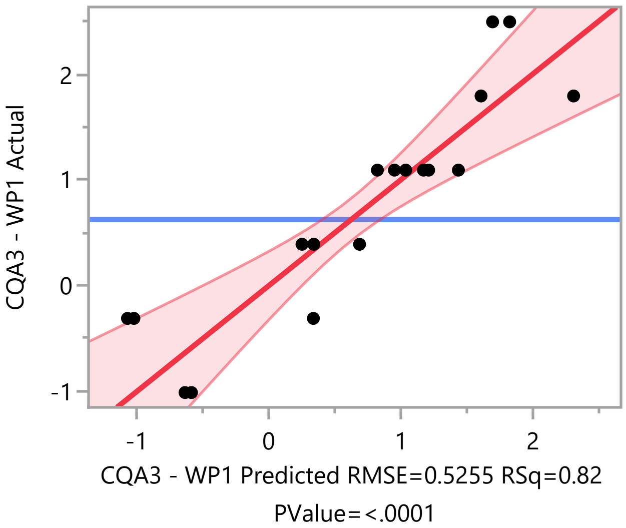

Effect Summary

Source	Logworth		PValue
PP 4(-0.43,1)	3.884		0.00013
PP 5(-0.91,1)	3.779		0.00017
PP 2*PP 5	2.024		0.00946
PP 4*PP 5	1.387		0.04106
PP 3(-0.33,1)	1.262		0.05475

Residual by Predicted Plot

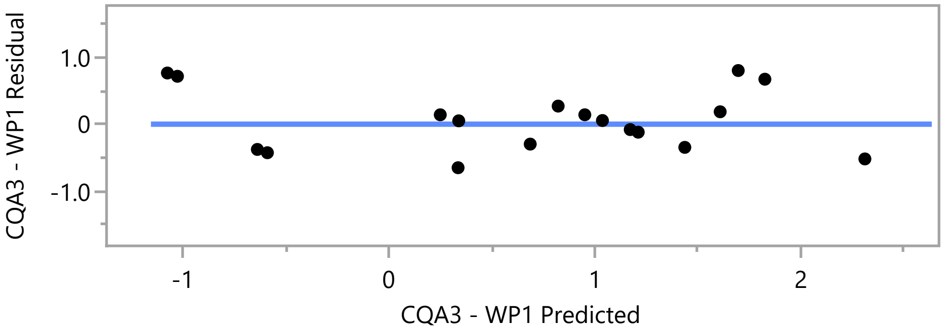

Fit Group

Response CQA3 - WP1

Studentized Residuals

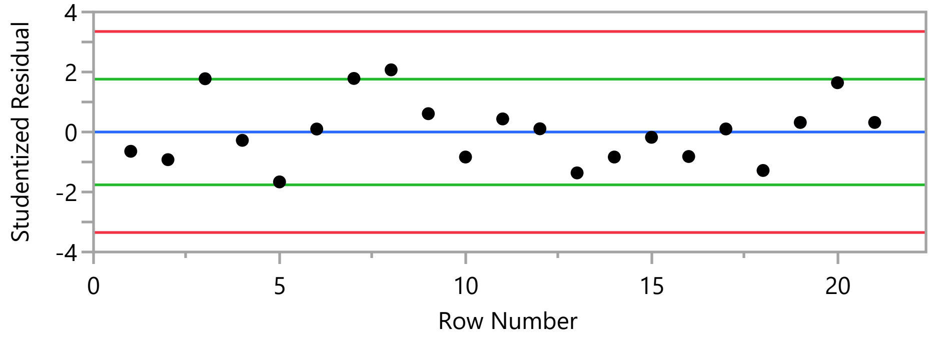

Externally studentized residuals with 90% simultaneous limits (Bonferroni) in red, individual limits in green.

Summary of Fit

RSquare	0.820236
RSquare Adj	0.760314
Root Mean Square Error	0.525451
Mean of Response	0.623385
Observations (or Sum Wgts)	21

Analysis of Variance

Source	DF	Sum of Squares	Mean Square	F Ratio
Model	5	18.896876	3.77938	13.6885
Error	15	4.141477	0.27610	**Prob > F**
C. Total	20	23.038352		<.0001*

Prediction Profiler

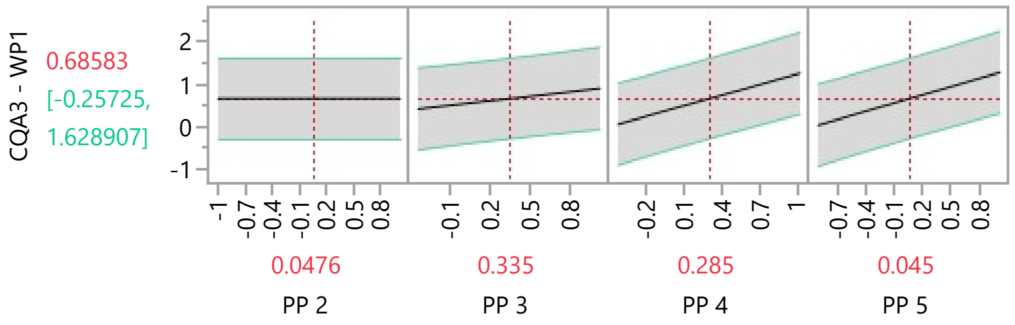

Press

Residual	SSE	RMSE	RSquare
Press	8.7039684417	0.64379709	0.6222
Ordinary	4.141476818	0.52545072	0.8202

Fit Group

Response CQA3 - WP1

Residual Normal Quantile Plot

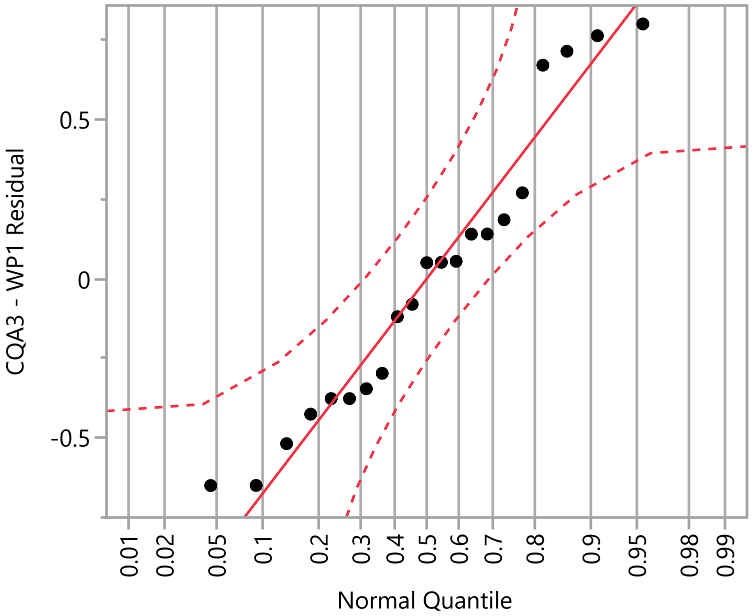

Graph Builder

Measured & Prediction vs. Experiment

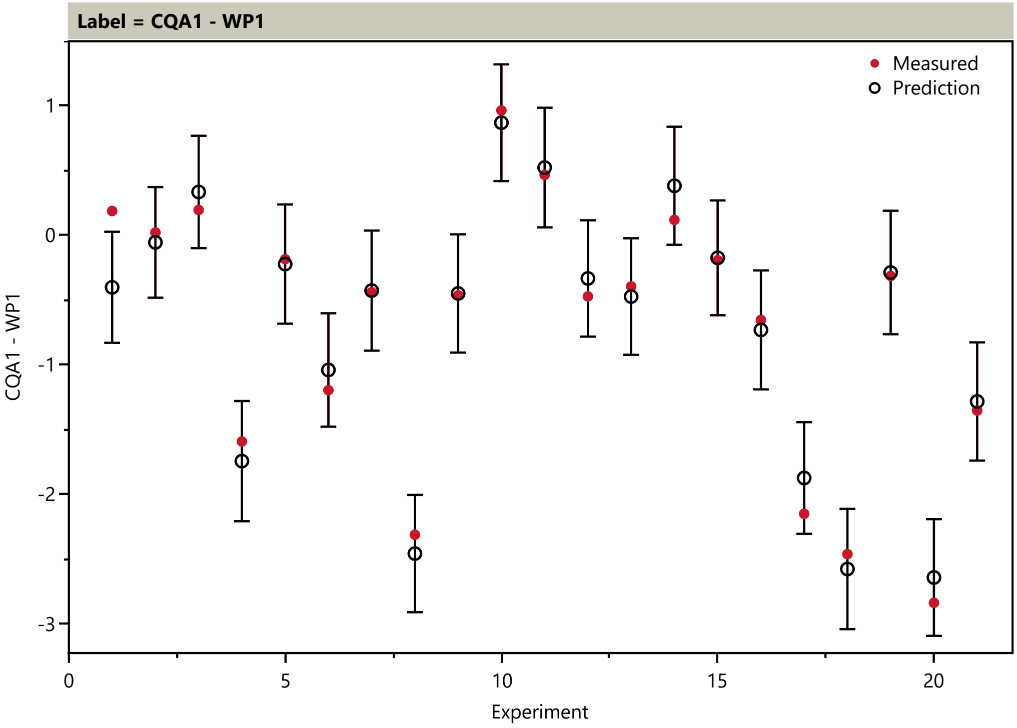

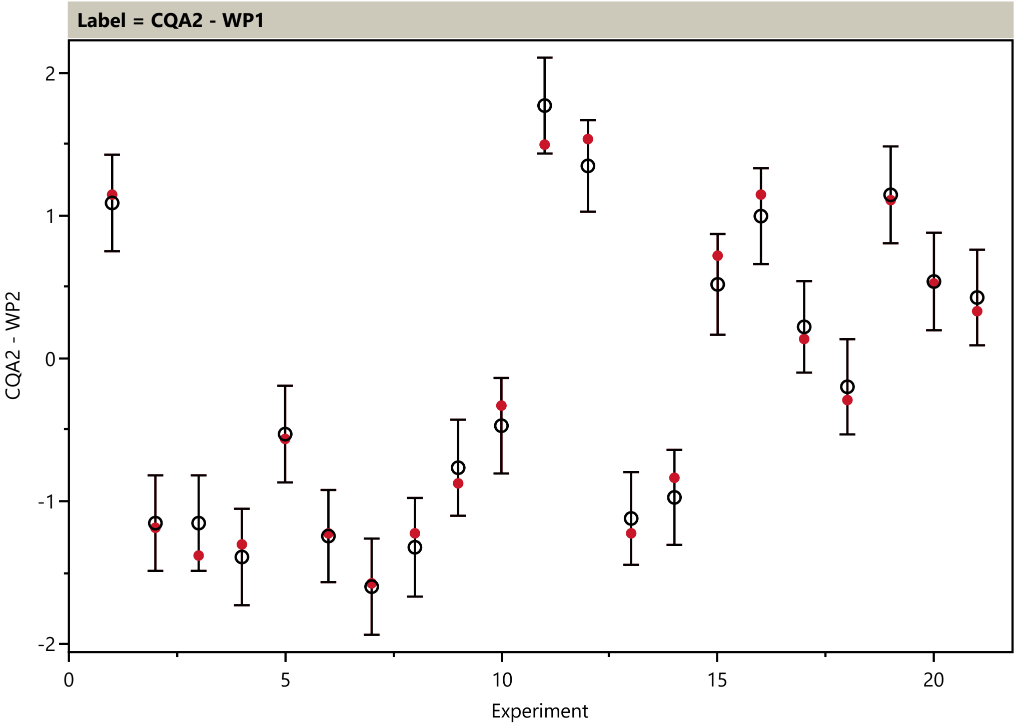

Graph Builder

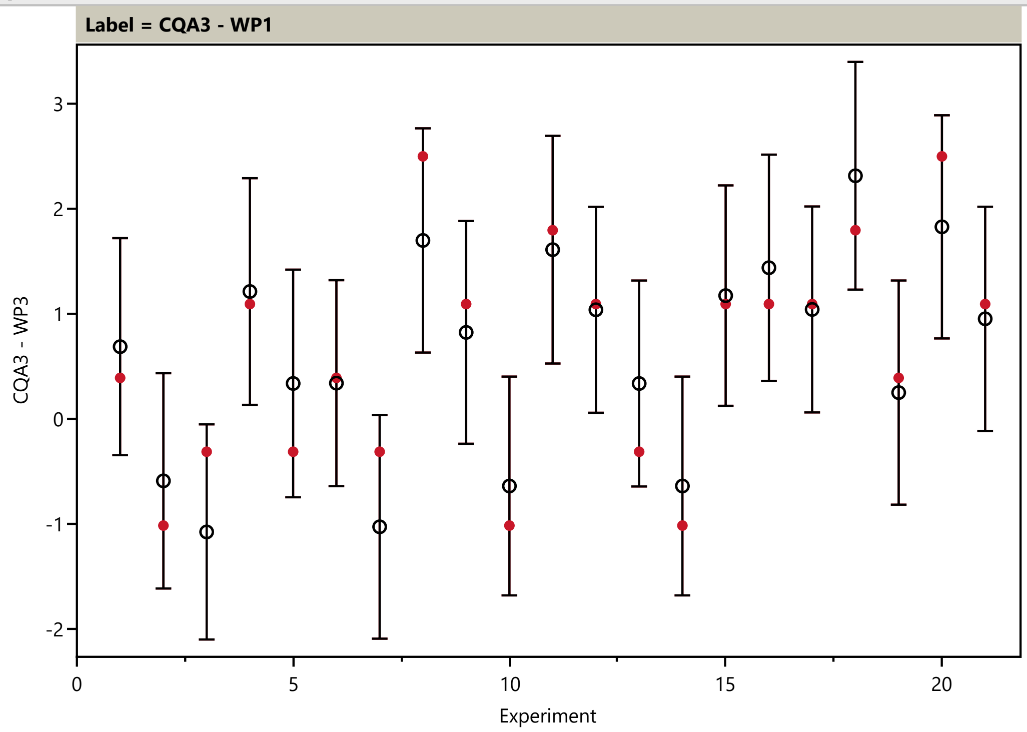

Each error bar is constructed from 90% lower PI to 90% upper PI.
