# Supplementary material for: Lifecycle DoE—The Companion for a Holistic Development Process
Source: Bioengineering (Basel). 2024 Oct 30;11(11):1089. doi: 10.3390/bioengineering11111089 (PMC11591819; doi:10.3390/bioengineering11111089)
Supplement: Supplementary file 1 [file bioengineering-11-01089-s001.zip › Supplementary_SB.pdf]

Supplementary SB. Summary Table – Process Optimization Analysis

Model	Setting	WP	Analytical Method	PP 1	PP 2	PP 3	PP 4	PP 5	PP 6	PP 7	PP 8	PP 9	CQA1 WP1-3	CQA2 WP1-3	CQA3 WP1-3	Desirability
Model_WP1	Initial	WP1	NA	-0.31	0.00	0.34	0.29	0.05	0.14	0.00	0.00	0.00	-0.73	-0.18	0.69	0.55
Model_WP1	Optimized	WP1	NA	0.38	0.39	-0.33	-0.43	-0.06	0.14	0.00	0.00	0.00	0.16	-1.16	-0.24	0.70
Model_WP1_3	Initial	NA	NA	0.38	0.39	-0.33	-0.43	-0.06	0.14	0.00	0.00	0.00	0.52	-1.01	0.18	0.72
Model_WP1_3	Optimized	NA	NA	0.86	0.00	-0.33	-0.82	-1.00	-0.71	0.00	0.00	0.00	1.73	-1.47	0.09	0.83
Model_WP1_4	Initial	WP4	NA	0.86	0.00	-0.33	-0.82	-1.00	-0.71	0.00	0.00	0.00	1.15	-0.42	-0.11	0.74
Model_WP1_4	Optimized	WP4	NA	0.50	0.00	-0.64	-0.71	-0.91	-0.43	0.00	0.00	0.00	0.85	-0.11	-0.25	0.68
Model_WP1_4	Final	WP4	NA	-0.23	0.00	-0.33	-0.43	-0.91	-0.43	0.00	0.00	0.00	0.87	0.13	-0.52	0.65
Model_WP1_7	Final	NA	B	-0.23	0.00	-0.33	-0.43	-0.91	-0.43	0.00	0.00	0.00	0.94	0.21	-0.68	NA
