# Supplementary material for: Lifecycle DoE—The Companion for a Holistic Development Process
Source: Bioengineering (Basel). 2024 Oct 30;11(11):1089. doi: 10.3390/bioengineering11111089 (PMC11591819; doi:10.3390/bioengineering11111089)
Supplement: Supplementary file 1 [file bioengineering-11-01089-s001.zip › Supplementary_SC_WP1_3.pdf]

Evaluate Design

Design

Run	Workpackage	PP 1	PP 2	PP 3	PP 4	PP 5	PP 6	PP 7	PP 8
1	Block 1	-0.53846	0	-0.33333	1	-0.90909	0.142857	0	0
2	Block 1	-0.53846	0	1	-0.42857	-0.90909	0.142857	0	0
3	Block 1	-0.53846	0	-0.33333	-0.42857	-0.90909	0.142857	0	0
4	Block 1	0.384615	-1	-0.33333	-0.42857	1	0.142857	0	0
5	Block 1	-0.07692	1	-0.33333	-0.42857	1	0.142857	0	0
6	Block 1	0.384615	-1	1	-0.42857	0.048485	0.142857	0	0
7	Block 1	-1	-1	1	-0.42857	-0.90909	0.142857	0	0
8	Block 1	-0.53846	-1	1	-0.42857	1	0.142857	0	0
9	Block 1	0.384615	1	1	-0.42857	1	0.142857	0	0
10	Block 1	0.384615	1	-0.33333	-0.42857	-0.90909	0.142857	0	0
11	Block 1	0.384615	1	1	1	-0.90909	0.142857	0	0
12	Block 1	0.384615	1	-0.33333	1	0.048485	0.142857	0	0
13	Block 1	-1	1	1	-0.42857	0.048485	0.142857	0	0
14	Block 1	-1	1	-0.33333	-0.42857	-0.90909	0.142857	0	0
15	Block 1	-1	0	1	1	-0.90909	0.142857	0	0
16	Block 1	-0.53846	1	1	1	1	0.142857	0	0
17	Block 1	-1	-1	-0.33333	1	0.048485	0.142857	0	0
18	Block 1	-1	-1	1	1	1	0.142857	0	0
19	Block 1	0.384615	-1	-0.33333	1	-0.90909	0.142857	0	0
20	Block 1	-0.07692	-1	-0.33333	1	1	0.142857	0	0
21	Block 1	-1	1	-0.33333	1	1	0.142857	0	0
22	Block 2	0.384615	0	-0.33333	1	0.048485	-0.71429	0	0
23	Block 2	-0.07692	-1	-0.33333	-0.42857	0.048485	1	0	0
24	Block 2	0.384615	-1	1	1	0.048485	0.142857	0	0
25	Block 2	-0.53846	-1	1	1	-0.90909	-0.71429	0	0
26	Block 2	-1	0	1	1	1	-0.71429	0	0
27	Block 2	-1	0	-0.33333	1	0.048485	1	0	0
28	Block 2	-0.53846	1	-0.33333	-0.42857	0.048485	-0.71429	0	0
29	Block 2	-1	1	1	1	-0.90909	0.142857	0	0
30	Block 2	-1	0	1	-0.42857	-0.90909	-0.71429	0	0
31	Block 2	-0.53846	0	-0.33333	-0.42857	0.048485	0.142857	0	0
32	Block 2	-0.07692	1	1	1	-0.90909	1	0	0
33	Block 2	0.384615	0	1	-0.42857	1	1	0	0
34	Block 2	-0.23077	0	-0.33333	-0.42857	-0.90909	0.142857	0	0
35	Block 2	-0.23077	0	-0.33333	-0.42857	-0.90909	0.142857	0	0
36	Block 3	-0.23077	0	-0.33333	-0.42857	-0.90909	0.142857	0	0
37	Block 3	-1	0	-0.33333	1	1	1	1	1
38	Block 3	1	0	-0.33333	1	-1	1	1	1
39	Block 3	1	0	-0.33333	1	-1	1	-1	-1
40	Block 3	-1	0	-0.33333	1	1	1	-1	-1
41	Block 3	1	0	-0.33333	1	1	1	1	-1
42	Block 3	1	0	-0.33333	1	1	-0.71429	1	1
43	Block 3	1	0	-0.33333	-1	-1	1	0	1
44	Block 3	1	0	-0.33333	1	1	1	-1	1
45	Block 3	-1	0	-0.33333	-1	-1	-0.71429	-1	-1
46	Block 3	1	0	-0.33333	-1	1	1	-1	-1
47	Block 3	1	0	-0.33333	1	1	-0.71429	-1	-1
48	Block 3	-0.23077	0	-0.33333	-0.42857	-0.90909	0.142857	0	0
49	Block 3	-1	0	-0.33333	-1	1	-0.71429	-1	1
50	Block 3	1	0	-0.33333	-1	-1	1	1	-1
51	Block 3	1	0	-0.33333	-1	1	1	1	1
52	Block 3	-1	0	-0.33333	1	-0.95152	1	1	-1
53	Block 3	-1	0	-0.33333	-1	1	-0.71429	1	-1
54	Block 3	-1	0	-0.33333	1	-0.95152	1	-1	1
55	Block 3	-1	0	-0.33333	-1	-1	-0.71429	1	1
56	Block 3	1	0	-0.33333	-1	-1	1	-1	0
57	Block 3	1	0	-0.33333	1	-1	-0.71429	-1	1
58	Block 3	1	0	-0.33333	1	-1	-0.71429	1	-1
59	Block 3	-0.23077	0	-0.33333	-0.42857	-0.90909	0.142857	0	0

Design Evaluation

Power Analysis

Significance Level	0.05
Anticipated RMSE	1

Evaluate Design

Design Evaluation

Power Analysis

Term	Anticipated Coefficient	Power
Intercept	2.303	0.8
Workpackage 1	1.118	0.722
Workpackage 2	-1.118	0.73
PP 1	1.399	0.801
PP 2	0.822	0.8
PP 3	0.697	0.801
PP 4	1.496	0.8
PP 5	0.636	0.8
PP 6	1.191	0.8
PP 7	0.719	0.8
PP 8	0.719	0.8
PP 1*PP 1	2.154	0.8
PP 1*PP 2	1.172	0.8
PP 1*PP 3	0.998	0.8
PP 1*PP 4	1.284	0.801
PP 1*PP 5	0.632	0.8
PP 1*PP 6	1.37	0.8
PP 1*PP 7	0.72	0.801
PP 1*PP 8	0.72	0.801
PP 2*PP 2	1.324	0.8
PP 2*PP 3	0.693	0.801
PP 2*PP 4	0.972	0.8
PP 2*PP 5	0.882	0.8
PP 2*PP 6	1.981	0.8
PP 3*PP 4	0.941	0.8
PP 3*PP 5	0.604	0.8
PP 3*PP 6	1.09	0.801
PP 4*PP 4	3.253	0.8
PP 4*PP 5	0.607	0.8
PP 4*PP 6	1.4	0.8
PP 4*PP 7	0.77	0.8
PP 4*PP 8	0.77	0.8
PP 5*PP 5	1.682	0.8
PP 5*PP 6	0.647	0.8
PP 5*PP 7	0.722	0.8
PP 5*PP 8	0.722	0.8
PP 6*PP 6	1.82	0.8
PP 6*PP 7	0.718	0.801
PP 6*PP 8	0.718	0.801
PP 7*PP 7	3.133	0.8
PP 7*PP 8	0.736	0.801
PP 8*PP 8	3.133	0.8

Effect	Power
Workpackage	0.801

Design and Anticipated Responses

Anticipated Response	Workpackage	PP 1	PP 2	PP 3	PP 4	PP 5	PP 6	PP 7	PP 8
7.364591	Block 1	-0.53846	0	-0.33333	1	-0.90909	0.142857	0	0
4.109005	Block 1	-0.53846	0	1	-0.42857	-0.90909	0.142857	0	0
5.703978	Block 1	-0.53846	0	-0.33333	-0.42857	-0.90909	0.142857	0	0
5.320939	Block 1	0.384615	-1	-0.33333	-0.42857	1	0.142857	0	0
6.328743	Block 1	-0.07692	1	-0.33333	-0.42857	1	0.142857	0	0
4.494625	Block 1	0.384615	-1	1	-0.42857	0.048485	0.142857	0	0
7.257256	Block 1	-1	-1	1	-0.42857	-0.90909	0.142857	0	0
5.598398	Block 1	-0.53846	-1	1	-0.42857	1	0.142857	0	0
11.348	Block 1	0.384615	1	1	-0.42857	1	0.142857	0	0
5.399087	Block 1	0.384615	1	-0.33333	-0.42857	-0.90909	0.142857	0	0
14.47835	Block 1	0.384615	1	1	1	-0.90909	0.142857	0	0
10.44011	Block 1	0.384615	1	-0.33333	1	0.048485	0.142857	0	0
5.286547	Block 1	-1	1	1	-0.42857	0.048485	0.142857	0	0
6.625587	Block 1	-1	1	-0.33333	-0.42857	-0.90909	0.142857	0	0
8.562231	Block 1	-1	0	1	1	-0.90909	0.142857	0	0
15.68712	Block 1	-0.53846	1	1	1	1	0.142857	0	0

Evaluate Design

Design Evaluation

Power Analysis

Design and Anticipated Responses

Anticipated Response	Workpackage	PP 1	PP 2	PP 3	PP 4	PP 5	PP 6	PP 7	PP 8
8.362232	Block 1	-1	-1	-0.33333	1	0.048485	0.142857	0	0
10.30256	Block 1	-1	-1	1	1	1	0.142857	0	0
8.658433	Block 1	0.384615	-1	-0.33333	1	-0.90909	0.142857	0	0
8.11116	Block 1	-0.07692	-1	-0.33333	1	1	0.142857	0	0
10.8084	Block 1	-1	1	-0.33333	1	1	0.142857	0	0
5.073323	Block 2	0.384615	0	-0.33333	1	0.048485	-0.71429	0	0
1.78412	Block 2	-0.07692	-1	-0.33333	-0.42857	0.048485	1	0	0
7.749518	Block 2	0.384615	-1	1	1	0.048485	0.142857	0	0
7.967003	Block 2	-0.53846	-1	1	1	-0.90909	-0.71429	0	0
7.807464	Block 2	-1	0	1	1	1	-0.71429	0	0
6.742845	Block 2	-1	0	-0.33333	1	0.048485	1	0	0
3.038876	Block 2	-0.53846	1	-0.33333	-0.42857	0.048485	-0.71429	0	0
8.158448	Block 2	-1	1	1	1	-0.90909	0.142857	0	0
4.933058	Block 2	-1	0	1	-0.42857	-0.90909	-0.71429	0	0
1.533063	Block 2	-0.53846	0	-0.33333	-0.42857	0.048485	0.142857	0	0
16.76424	Block 2	-0.07692	1	1	1	-0.90909	1	0	0
10.04383	Block 2	0.384615	0	1	-0.42857	1	1	0	0
2.732843	Block 2	-0.23077	0	-0.33333	-0.42857	-0.90909	0.142857	0	0
2.732843	Block 2	-0.23077	0	-0.33333	-0.42857	-0.90909	0.142857	0	0
3.850843	Block 3	-0.23077	0	-0.33333	-0.42857	-0.90909	0.142857	0	0
21.5783	Block 3	-1	0	-0.33333	1	1	1	1	1
25.10435	Block 3	1	0	-0.33333	1	-1	1	1	1
16.28435	Block 3	1	0	-0.33333	1	-1	1	-1	-1
12.7423	Block 3	-1	0	-0.33333	1	1	1	-1	-1
23.0523	Block 3	1	0	-0.33333	1	1	1	1	-1
21.91871	Block 3	1	0	-0.33333	1	1	-0.71429	1	1
12.23578	Block 3	1	0	-0.33333	-1	-1	1	0	1
23.0523	Block 3	1	0	-0.33333	1	1	1	-1	1
26.84686	Block 3	-1	0	-0.33333	-1	-1	-0.71429	-1	-1
12.62373	Block 3	1	0	-0.33333	-1	1	1	-1	-1
13.08111	Block 3	1	0	-0.33333	1	1	-0.71429	-1	-1
3.850843	Block 3	-0.23077	0	-0.33333	-0.42857	-0.90909	0.142857	0	0
17.22512	Block 3	-1	0	-0.33333	-1	1	-0.71429	-1	1
13.96778	Block 3	1	0	-0.33333	-1	-1	1	1	-1
21.05973	Block 3	1	0	-0.33333	-1	1	1	1	1
14.25877	Block 3	-1	0	-0.33333	1	-0.95152	1	1	-1
17.22512	Block 3	-1	0	-0.33333	-1	1	-0.71429	1	-1
14.25877	Block 3	-1	0	-0.33333	1	-0.95152	1	-1	1
17.98846	Block 3	-1	0	-0.33333	-1	-1	-0.71429	1	1
10.90578	Block 3	1	0	-0.33333	-1	-1	1	-1	0
14.79245	Block 3	1	0	-0.33333	1	-1	-0.71429	-1	1
14.79245	Block 3	1	0	-0.33333	1	-1	-0.71429	1	-1
3.850843	Block 3	-0.23077	0	-0.33333	-0.42857	-0.90909	0.142857	0	0

Evaluate Design

Design Evaluation

Color Map on Correlations

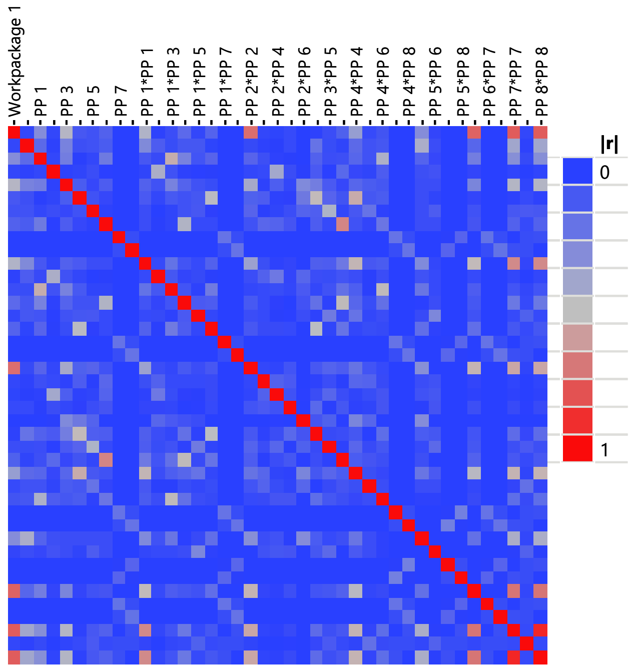

Fit Group

Response CQA1 - WP1-3

Actual by Predicted Plot

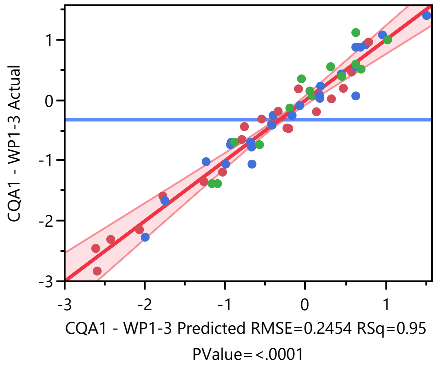

Effect Summary

Source	Logworth		PValue
PP 5	20.426		0.00000
PP 2	13.956		0.00000
PP 2*PP 2	6.799		0.00000
PP 5*PP 6	5.457		0.00000
PP 7	4.486		0.00003
PP 6*PP 6	4.371		0.00004
PP 4*PP 4	3.889		0.00013
PP 2*PP 5	3.022		0.00095
PP 1	2.769		0.00170
PP 8*PP 8	2.536		0.00291
PP 6(-0.71,1)	2.368		0.00428 ^
PP 3*PP 4	2.331		0.00467
PP 2*PP 6	1.737		0.01833
PP 4	1.578		0.02641 ^
PP 6*PP 8	1.511		0.03084
PP 1*PP 1*PP 1	0.450		0.35501

Lack Of Fit

Source	DF	Sum of Squares	Mean Square	F Ratio
Lack Of Fit	38	1.9072667	0.050191	0.3224
Pure Error	4	0.6227410	0.155685	**Prob > F**
Total Error	42	2.5300078		0.9735
				**Max RSq**
				0.9886

Residual by Predicted Plot

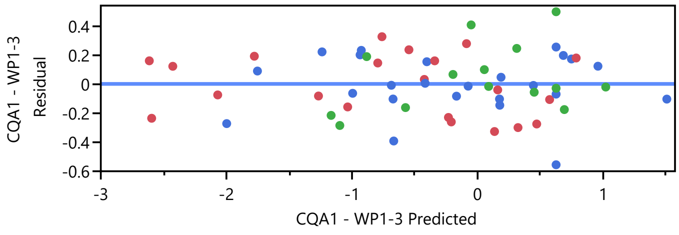

Studentized Residuals

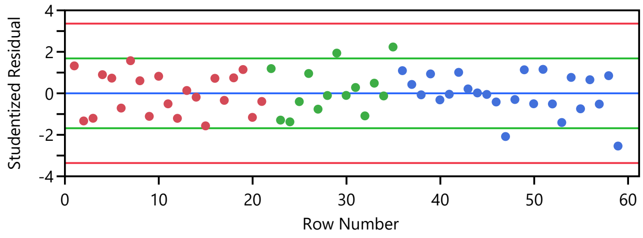

Externally studentized residuals with 90% simultaneous limits (Bonferroni) in red, individual limits in green.

Fit Group

Response CQA1 - WP1-3

Parameter Estimates

Term	Estimate	Std Error	t Ratio	Prob>|t|
Intercept	0.2495075	0.108999	2.29	0.0272*
PP 1	0.5826496	0.173761	3.35	0.0017*
PP 2	0.607915	0.052393	11.60	<.0001*
PP 4	0.1719421	0.074718	2.30	0.0264*
PP 5	-0.696917	0.039214	-17.77	<.0001*
PP 6(-0.71,1)	-0.141223	0.046753	-3.02	0.0043*
PP 7	-0.257278	0.055302	-4.65	<.0001*
PP 2*PP 2	-0.573682	0.091405	-6.28	<.0001*
PP 3*PP 4	0.1736599	0.058114	2.99	0.0047*
PP 4*PP 4	-0.681617	0.161658	-4.22	0.0001*
PP 2*PP 5	0.2419099	0.068038	3.56	0.0009*
PP 2*PP 6	0.3271902	0.1333	2.45	0.0183*
PP 5*PP 6	0.2680826	0.050192	5.34	<.0001*
PP 6*PP 6	0.4344234	0.095085	4.57	<.0001*
PP 6*PP 8	-0.122531	0.05484	-2.23	0.0308*
PP 8*PP 8	-0.393434	0.124429	-3.16	0.0029*
PP 1*PP 1*PP 1	-0.170968	0.182807	-0.94	0.3550

Residual by Row Plot

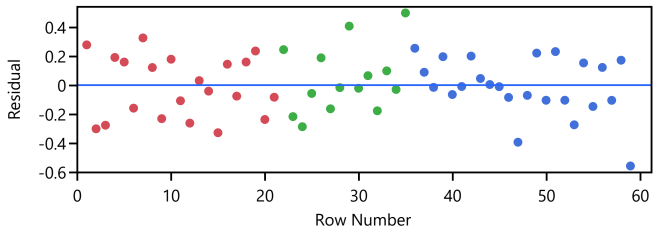

Prediction Profiler

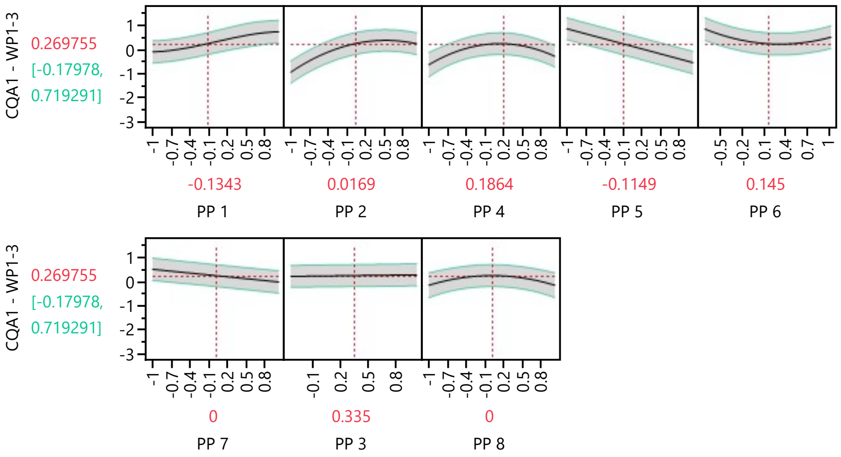

Residual Normal Quantile Plot

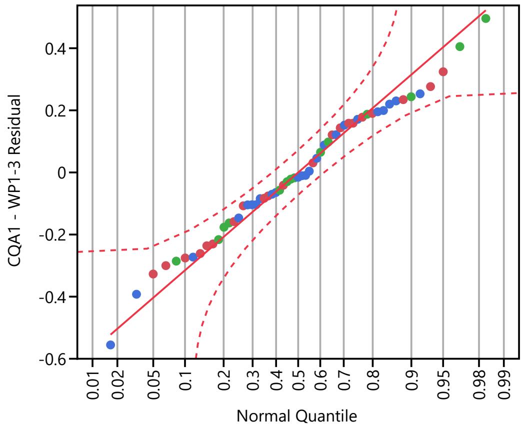

Press

Residual	SSE	RMSE	RSquare
Press	4.7651867397	0.28419338	0.9125
Ordinary	2.5300077783	0.24543488	0.9536

Fit Group

Response CQA2 - WP1-3

Actual by Predicted Plot

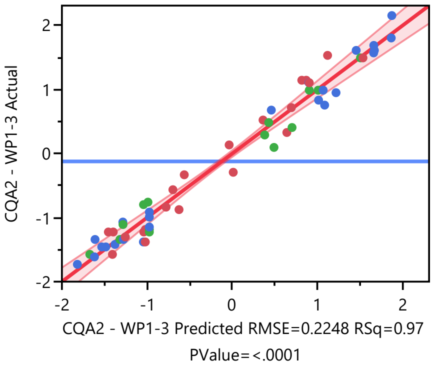

Effect Summary

Source	Logworth	PValue
PP 4	33.931	0.00000
PP 2	7.405	0.00000
PP 1	5.511	0.00000
PP 8*PP 8	2.763	0.00173
PP 1*PP 4	2.331	0.00467
PP 5	2.242	0.00572
PP 4*PP 5	2.099	0.00797
PP 5*PP 5	1.844	0.01432
PP 8	1.515	0.03055 ^
PP 5*PP 8	1.437	0.03659
PP 3*PP 6	1.241	0.05743
PP 2*PP 6	1.237	0.05789

Lack Of Fit

Source	DF	Sum of Squares	Mean Square	F Ratio
Lack Of Fit	42	2.2533267	0.053651	3.0099
Pure Error	4	0.0712978	0.017824	**Prob > F**
Total Error	46	2.3246245		0.1453
				**Max RSq**
				0.9991

Residual by Predicted Plot

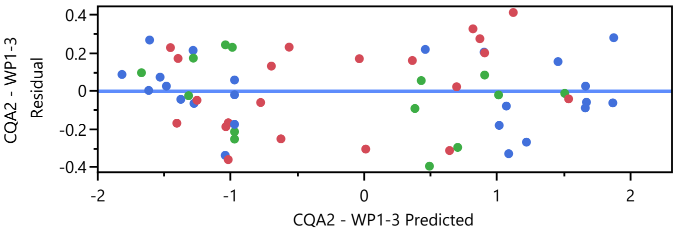

Studentized Residuals

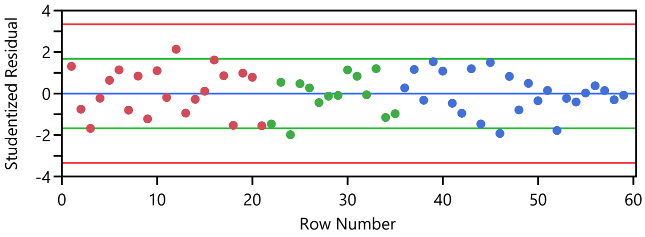

Externally studentized residuals with 90% simultaneous limits (Bonferroni) in red, individual limits in green.

Parameter Estimates

Term	Estimate	Std Error	t Ratio	Prob>|t|
Intercept	-0.636982	0.077804	-8.19	<.0001*
PP 1	0.222133	0.041833	5.31	<.0001*
PP 2	0.3156508	0.047993	6.58	<.0001*
PP 4	1.3063711	0.037607	34.74	<.0001*
PP 5	-0.10355	0.035723	-2.90	0.0057*

Fit Group

Response CQA2 - WP1-3

Parameter Estimates

Term	Estimate	Std Error	t Ratio	Prob>|t|
PP 8	-0.112183	0.050268	-2.23	0.0305*
PP 1*PP 4	0.1577984	0.053061	2.97	0.0047*
PP 4*PP 5	-0.111997	0.04037	-2.77	0.0080*
PP 5*PP 5	0.2531689	0.099445	2.55	0.0143*
PP 2*PP 6	0.2476273	0.127307	1.95	0.0579
PP 3*PP 6	-0.101642	0.052157	-1.95	0.0574
PP 5*PP 8	-0.108744	0.050507	-2.15	0.0366*
PP 8*PP 8	0.2513174	0.075508	3.33	0.0017*

Residual by Row Plot

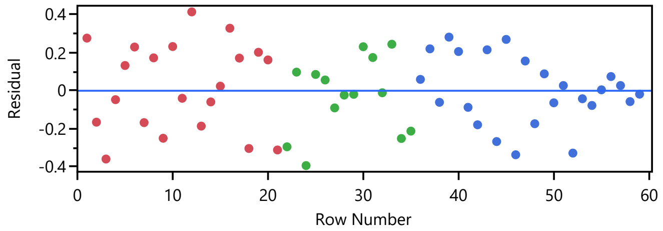

Prediction Profiler

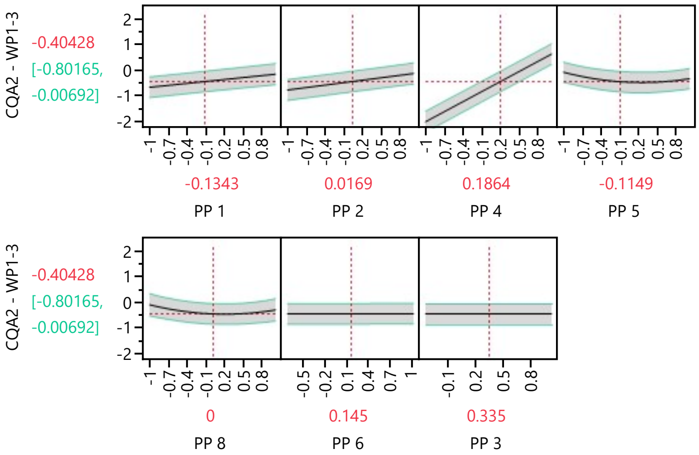

Residual Normal Quantile Plot

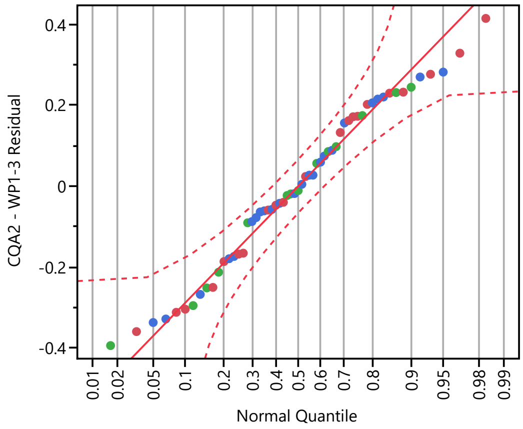

Press

Residual	SSE	RMSE	RSquare
Press	3.7933667814	0.25356331	0.9536
Ordinary	2.3246244923	0.22480061	0.9715

Fit Group

Response CQA3 - WP1-3

Actual by Predicted Plot

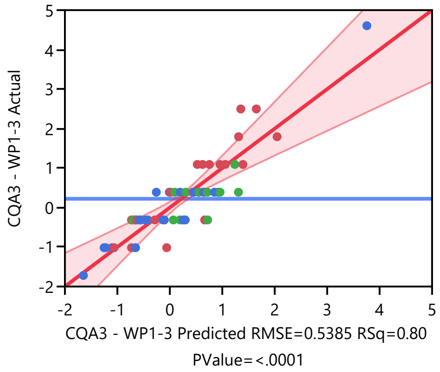

Effect Summary

Source	Logworth	PValue
PP 5	8.343	0.00000
PP 8*PP 8	5.088	0.00001
PP 4*PP 5	5.046	0.00001
PP 4*PP 4	3.828	0.00015
PP 7	3.193	0.00064
PP 4*PP 6	2.450	0.00355
PP 1*PP 5	2.033	0.00927
PP 1*PP 2	1.972	0.01066
PP 4*PP 7	1.879	0.01322
PP 3*PP 8	1.701	0.01991
PP 7*PP 8	1.650	0.02241
PP 2*PP 5	1.276	0.05291
PP 1*PP 7	1.185	0.06532
PP 5*PP 7	1.130	0.07405
PP 6*PP 8	1.118	0.07625

Lack Of Fit

Source	DF	Sum of Squares	Mean Square	F Ratio
Lack Of Fit	39	11.877782	0.304559	2.0564
Pure Error	4	0.592415	0.148104	**Prob > F**
Total Error	43	12.470197		0.2543
				**Max RSq**
				0.9905

Residual by Predicted Plot

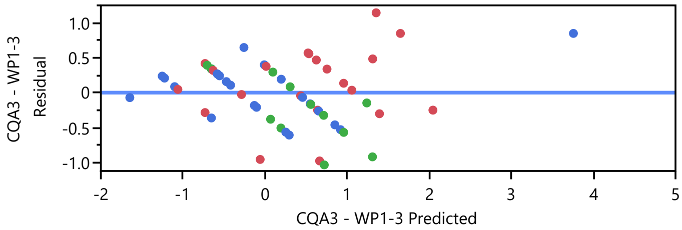

Studentized Residuals

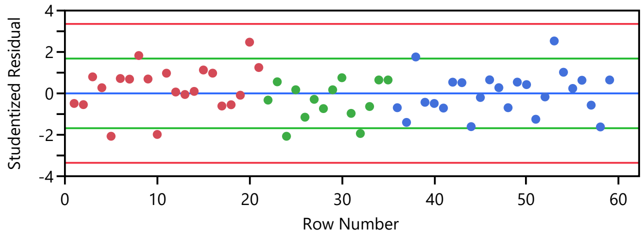

Externally studentized residuals with 90% simultaneous limits (Bonferroni) in red, individual limits in green.

Fit Group

Response CQA3 - WP1-3

Parameter Estimates

Term	Estimate	Std Error	t Ratio	Prob>|t|
Intercept	-0.021113	0.152532	-0.14	0.8906
PP 5	0.6084641	0.083201	7.31	<.0001*
PP 7	0.4623572	0.125571	3.68	0.0006*
PP 1*PP 2	0.4723083	0.176892	2.67	0.0107*
PP 4*PP 4	0.9103882	0.218772	4.16	0.0001*
PP 1*PP 5	-0.276882	0.101626	-2.72	0.0093*
PP 2*PP 5	-0.283069	0.142206	-1.99	0.0529
PP 4*PP 5	-0.484191	0.096148	-5.04	<.0001*
PP 4*PP 6	0.330629	0.107164	3.09	0.0035*
PP 1*PP 7	-0.237521	0.125579	-1.89	0.0653
PP 4*PP 7	-0.326462	0.126296	-2.58	0.0132*
PP 5*PP 7	0.2264508	0.123684	1.83	0.0741
PP 3*PP 8	0.2990292	0.123666	2.42	0.0199*
PP 6*PP 8	0.2253097	0.124028	1.82	0.0763
PP 7*PP 8	-0.306071	0.129211	-2.37	0.0224*
PP 8*PP 8	-0.920419	0.181707	-5.07	<.0001*

Residual by Row Plot

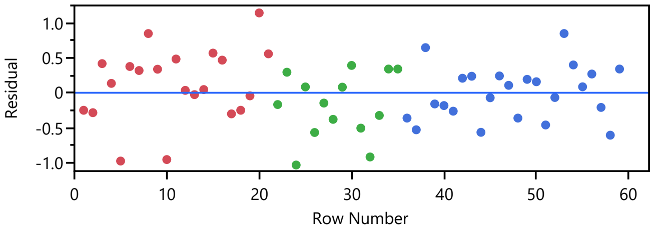

Prediction Profiler

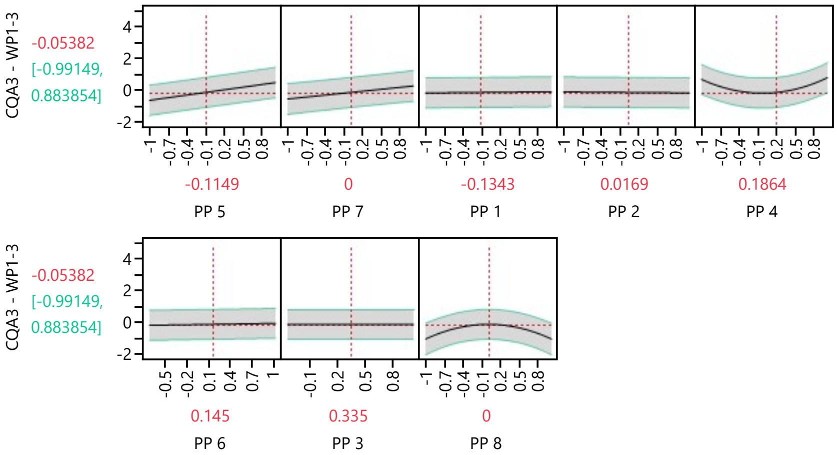

Residual Normal Quantile Plot

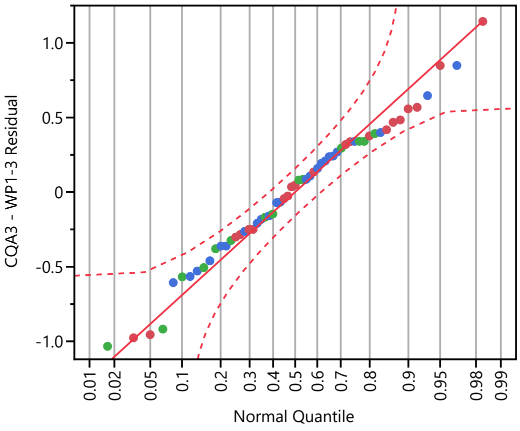

Press

Residual	SSE	RMSE	RSquare
Press	26.473139539	0.6698487	0.5767
Ordinary	12.470197029	0.53852074	0.8006

Graph Builder

Measured & Prediction vs. Experiment

Label = CQA1 - WP1-3

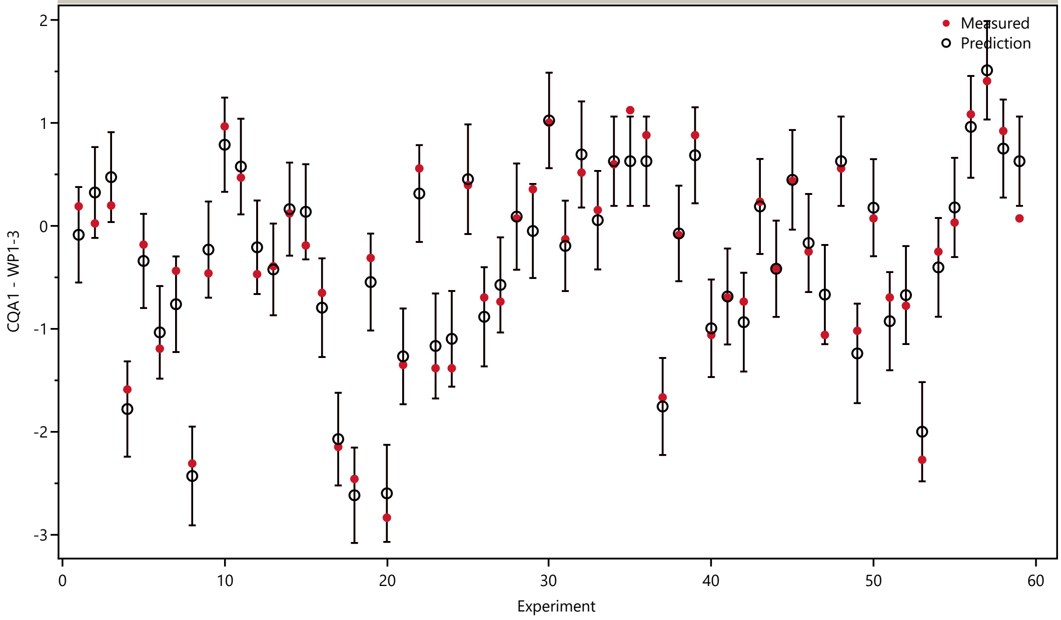

Label = CQA2 - WP1-3

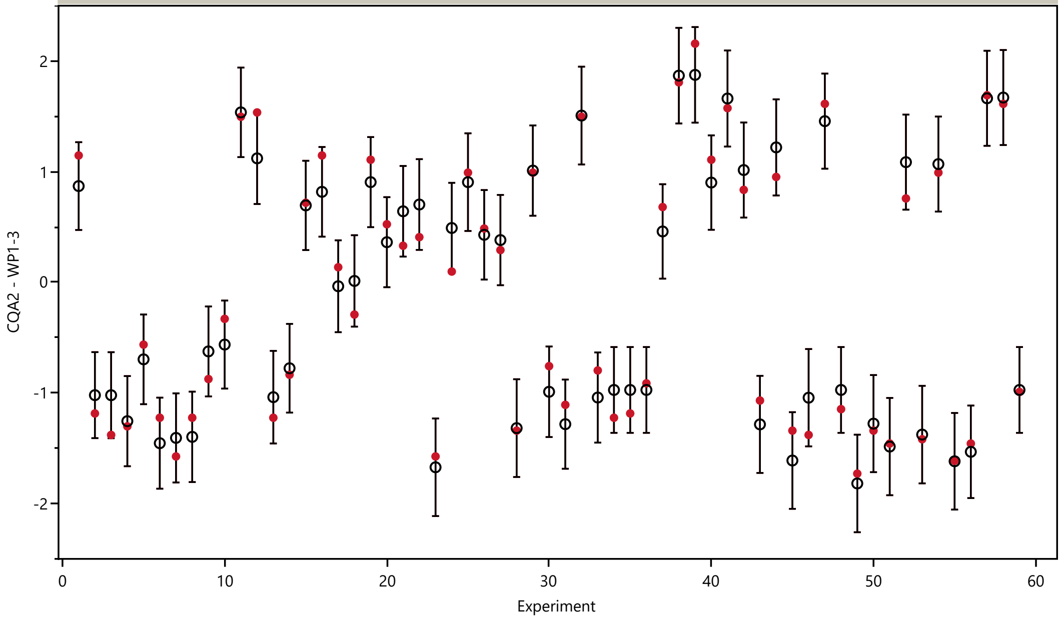

Graph Builder

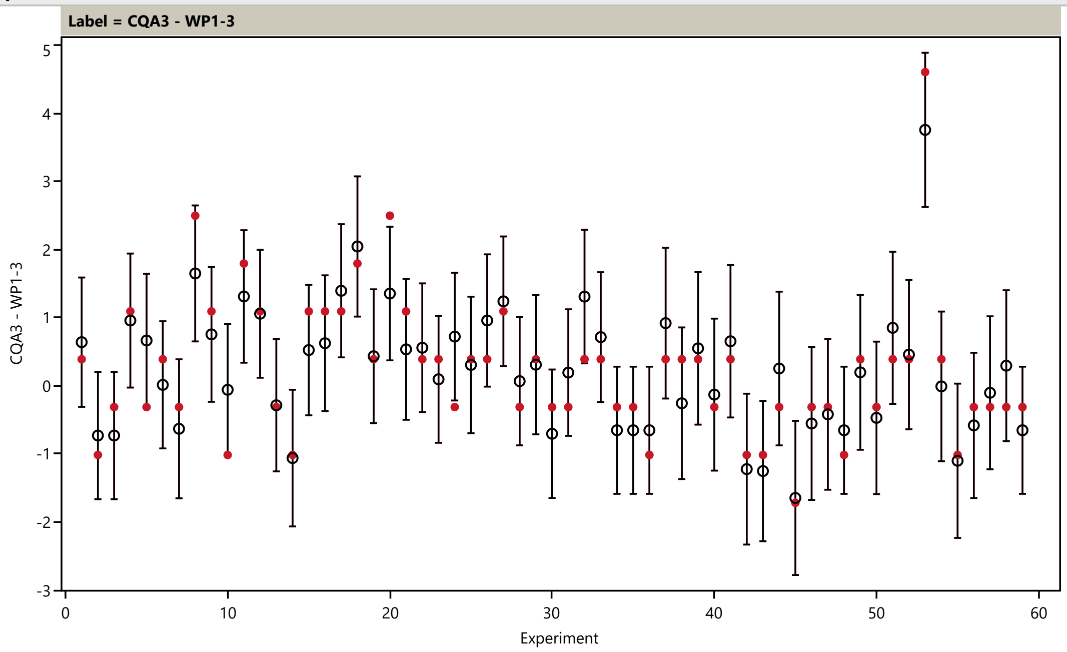

Each error bar is constructed from 90% lower PI to 90% upper PI.
