# Supplementary material for: Lifecycle DoE—The Companion for a Holistic Development Process
Source: Bioengineering (Basel). 2024 Oct 30;11(11):1089. doi: 10.3390/bioengineering11111089 (PMC11591819; doi:10.3390/bioengineering11111089)
Supplement: Supplementary file 1 [file bioengineering-11-01089-s001.zip › Supplementary_SD_WP1_4.pdf]

Evaluate Design

Design

Run		PP 1	PP 2	PP 3	PP 4	PP 5	PP 6	PP 7	PP 8	PP 9
1	WP1	-0.53846	0	-0.33333	1	-0.90909	0.142857	0	0	0
2	WP1	-0.53846	0	1	-0.42857	-0.90909	0.142857	0	0	0
3	WP1	-0.53846	0	-0.33333	-0.42857	-0.90909	0.142857	0	0	0
4	WP1	0.384615	-1	-0.33333	-0.42857	1	0.142857	0	0	0
5	WP1	-0.07692	1	-0.33333	-0.42857	1	0.142857	0	0	0
6	WP1	0.384615	-1	1	-0.42857	0.048485	0.142857	0	0	0
7	WP1	-1	-1	1	-0.42857	-0.90909	0.142857	0	0	0
8	WP1	-0.53846	-1	1	-0.42857	1	0.142857	0	0	0
9	WP1	0.384615	1	1	-0.42857	1	0.142857	0	0	0
10	WP1	0.384615	1	-0.33333	-0.42857	-0.90909	0.142857	0	0	0
11	WP1	0.384615	1	1	1	-0.90909	0.142857	0	0	0
12	WP1	0.384615	1	-0.33333	1	0.048485	0.142857	0	0	0
13	WP1	-1	1	1	-0.42857	0.048485	0.142857	0	0	0
14	WP1	-1	1	-0.33333	-0.42857	-0.90909	0.142857	0	0	0
15	WP1	-1	0	1	1	-0.90909	0.142857	0	0	0
16	WP1	-0.53846	1	1	1	1	0.142857	0	0	0
17	WP1	-1	-1	-0.33333	1	0.048485	0.142857	0	0	0
18	WP1	-1	-1	1	1	1	0.142857	0	0	0
19	WP1	0.384615	-1	-0.33333	1	-0.90909	0.142857	0	0	0
20	WP1	-0.07692	-1	-0.33333	1	1	0.142857	0	0	0
21	WP1	-1	1	-0.33333	1	1	0.142857	0	0	0
22	WP2	0.384615	0	-0.33333	1	0.048485	-0.71429	0	0	0
23	WP2	-0.07692	-1	-0.33333	-0.42857	0.048485	1	0	0	0
24	WP2	0.384615	-1	1	1	0.048485	0.142857	0	0	0
25	WP2	-0.53846	-1	1	1	-0.90909	-0.71429	0	0	0
26	WP2	-1	0	1	1	1	-0.71429	0	0	0
27	WP2	-1	0	-0.33333	1	0.048485	1	0	0	0
28	WP2	-0.53846	1	-0.33333	-0.42857	0.048485	-0.71429	0	0	0
29	WP2	-1	1	1	1	-0.90909	0.142857	0	0	0
30	WP2	-1	0	1	-0.42857	-0.90909	-0.71429	0	0	0
31	WP2	-0.53846	0	-0.33333	-0.42857	0.048485	0.142857	0	0	0
32	WP2	-0.07692	1	1	1	-0.90909	1	0	0	0
33	WP2	0.384615	0	1	-0.42857	1	1	0	0	0
34	WP2	-0.23077	0	-0.33333	-0.42857	-0.90909	0.142857	0	0	0
35	WP2	-0.23077	0	-0.33333	-0.42857	-0.90909	0.142857	0	0	0
36	WP3	-0.23077	0	-0.33333	-0.42857	-0.90909	0.142857	0	0	0
37	WP3	-1	0	-0.33333	1	1	1	1	1	0
38	WP3	1	0	-0.33333	1	-1	1	1	1	0
39	WP3	1	0	-0.33333	1	-1	1	-1	-1	0
40	WP3	-1	0	-0.33333	1	1	1	-1	-1	0
41	WP3	1	0	-0.33333	1	1	1	1	-1	0
42	WP3	1	0	-0.33333	1	1	-0.71429	1	1	0
43	WP3	1	0	-0.33333	-1	-1	1	0	1	0
44	WP3	1	0	-0.33333	1	1	1	-1	1	0
45	WP3	-1	0	-0.33333	-1	-1	-0.71429	-1	-1	0
46	WP3	1	0	-0.33333	-1	1	1	-1	-1	0
47	WP3	1	0	-0.33333	1	1	-0.71429	-1	-1	0
48	WP3	-0.23077	0	-0.33333	-0.42857	-0.90909	0.142857	0	0	0
49	WP3	-1	0	-0.33333	-1	1	-0.71429	-1	1	0
50	WP3	1	0	-0.33333	-1	-1	1	1	-1	0
51	WP3	1	0	-0.33333	-1	1	1	1	1	0
52	WP3	-1	0	-0.33333	1	-0.95152	1	1	-1	0
53	WP3	-1	0	-0.33333	-1	1	-0.71429	1	-1	0
54	WP3	-1	0	-0.33333	1	-0.95152	1	-1	1	0
55	WP3	-1	0	-0.33333	-1	-1	-0.71429	1	1	0
56	WP3	1	0	-0.33333	-1	-1	1	-1	0	0
57	WP3	1	0	-0.33333	1	-1	-0.71429	-1	1	0
58	WP3	1	0	-0.33333	1	-1	-0.71429	1	-1	0
59	WP3	-0.23077	0	-0.33333	-0.42857	-0.90909	0.142857	0	0	0
60	WP4	-0.23077	0	-0.33333	-0.42857	-0.90909	-0.42857	0	0	0
61	WP4	-0.23077	0	-0.33333	-0.42857	-0.90909	-0.42857	0	0	0
62	WP4	-0.23077	0	-0.33333	-0.42857	-0.90909	-0.42857	0	0	0
63	WP4	-0.23077	0	-0.33333	-0.42857	-0.90909	-0.42857	0	0	0
64	WP4	-0.53846	-1	1	-0.42857	-1	-0.42857	1	-1	1
65	WP4	-0.07692	-1	-1	-0.42857	0.048485	-0.42857	-1	1	1
66	WP4	-1	0	1	-0.42857	0.048485	-1	-1	1	1
67	WP4	-0.53846	1	1	-0.71429	-0.95152	0.142857	-1	-1	0
68	WP4	0.384615	0	-0.33333	-0.14286	-0.90909	-1	0	0	-1
69	WP4	-0.07692	-1	-1	-1	0.048485	0.142857	1	-1	1
70	WP4	-0.07692	0	-0.33333	-0.14286	1	1	1	-1	0
71	WP4	-0.53846	-1	1	-0.42857	-0.90909	-1	-1	-1	-1
72	WP4	-0.53846	-1	0.333333	-0.71429	-0.90909	-0.42857	1	-1	0
73	WP4	-0.53846	0	-1	-0.71429	-1	0.142857	0	0	1
74	WP4	-1	0	-1	1	1	1	1	-1	-1
75	WP4	-0.53846	1	0.333333	-0.42857	-0.90909	0.142857	-1	-1	1
76	WP4	-1	1	1	-0.71429	1	-1	0	0	1

Evaluate Design

Design

Run		PP 1	PP 2	PP 3	PP 4	PP 5	PP 6	PP 7	PP 8	PP 9
77	WP4	0.384615	0	-0.33333	-0.71429	0.048485	1	0	1	-1
78	WP4	-0.53846	0	-1	-0.14286	0.048485	-1	-1	0	0
79	WP4	-0.53846	-1	1	-0.14286	-0.90909	-0.42857	-1	-1	0
80	WP4	-0.07692	-1	1	-0.71429	0.048485	0.142857	-1	1	1
81	WP4	-0.53846	0	1	1	1	1	-1	-1	1

Design Evaluation

Power Analysis

Significance Level 0.05

Anticipated RMSE 1

Term	Anticipated Coefficient	Power
Intercept	1.933	0.8
Workpackage 1	0.863	0.481
Workpackage 2	-0.863	0.517
Workpackage 3	0.863	0.431
PP 1	1.163	0.8
PP 2	0.928	0.801
PP 3	2.025	0.8
PP 4	1.115	0.801
PP 5	0.533	0.8
PP 6	1.128	0.801
PP 7	0.771	0.801
PP 8	0.848	0.801
PP 9	2.254	0.8
PP 1*PP 1	1.887	0.8
PP 1*PP 2	1.151	0.8
PP 1*PP 3	1.475	0.8
PP 1*PP 4	1.028	0.801
PP 1*PP 5	0.612	0.8
PP 1*PP 6	1.231	0.8
PP 1*PP 7	0.702	0.8
PP 1*PP 8	0.7	0.801
PP 1*PP 9	3.976	0.8
PP 2*PP 2	1.24	0.801
PP 2*PP 3	0.998	0.8
PP 2*PP 4	0.939	0.8
PP 2*PP 5	0.866	0.801
PP 2*PP 6	2.109	0.8
PP 2*PP 7	1.943	0.8
PP 2*PP 8	2.219	0.8
PP 2*PP 9	2.454	0.8
PP 3*PP 3	2.856	0.8
PP 3*PP 4	1.282	0.8
PP 3*PP 5	0.88	0.8
PP 3*PP 6	1.716	0.8
PP 3*PP 7	1.721	0.8
PP 3*PP 8	1.818	0.8
PP 3*PP 9	2.36	0.8
PP 4*PP 4	2.127	0.8
PP 4*PP 5	0.579	0.801
PP 4*PP 6	1.35	0.8
PP 4*PP 7	0.74	0.801
PP 4*PP 8	0.738	0.8
PP 4*PP 9	3.91	0.8
PP 5*PP 5	1.391	0.8
PP 5*PP 6	0.728	0.801
PP 5*PP 7	0.688	0.801
PP 5*PP 8	0.687	0.801
PP 5*PP 9	2.323	0.8
PP 6*PP 6	2.253	0.8
PP 6*PP 7	0.817	0.801
PP 6*PP 8	0.786	0.8
PP 6*PP 9	2.328	0.8
PP 7*PP 7	2.483	0.8
PP 7*PP 8	0.709	0.8
PP 7*PP 9	2.586	0.8
PP 8*PP 8	2.774	0.8
PP 8*PP 9	2.234	0.8
PP 9*PP 9	2.447	0.8

Effect	Power
Workpackage	0.801

Evaluate Design

Design Evaluation

Color Map on Correlations

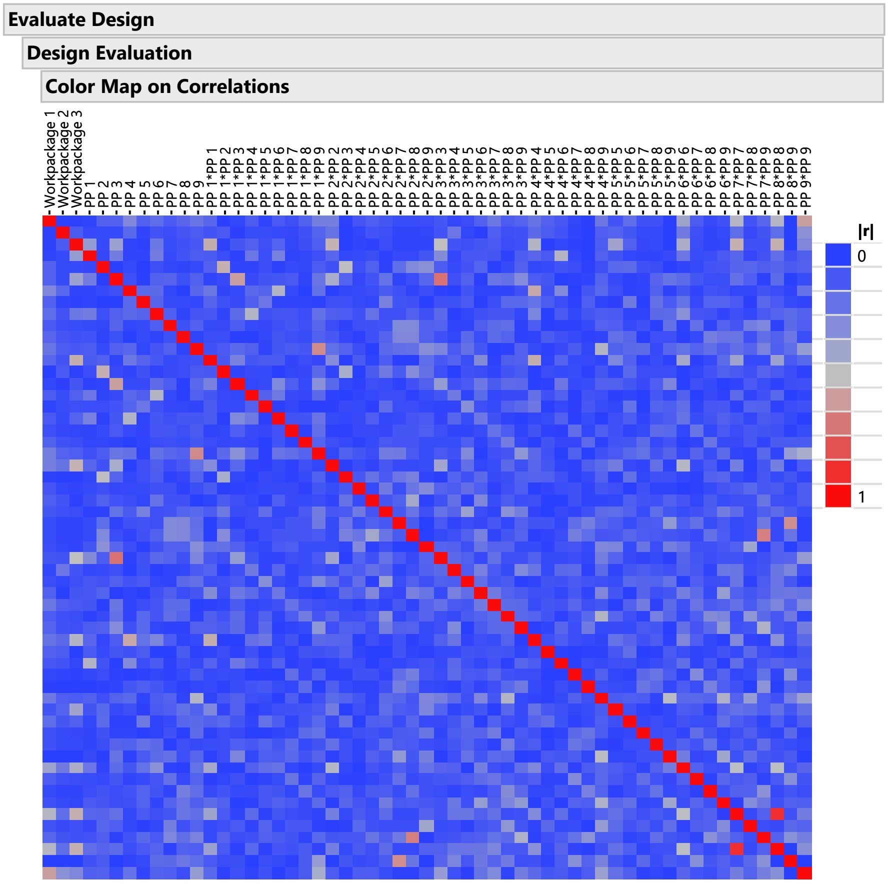

Output Options

Fit Group

Response CQA1 - WP1-4

Actual by Predicted Plot

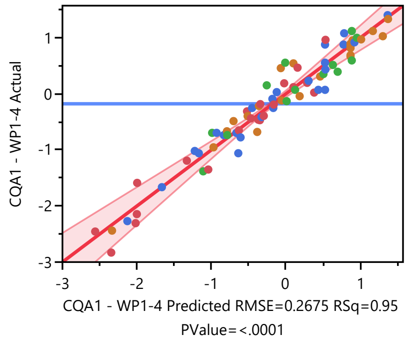

Effect Summary

Source	Logworth	PValue
PP 5	24.521	0.00000
PP 2	14.164	0.00000
PP 9	9.433	0.00000
PP 1*PP 1*PP 1	6.341	0.00000
PP 7	5.990	0.00000
Workpackage	4.655	0.00002
PP 5*PP 6	4.526	0.00003
PP 2*PP 5	3.737	0.00018
PP 4*PP 4	3.387	0.00041
PP 2*PP 2	2.195	0.00639
PP 2*PP 6	2.028	0.00937
PP 6*PP 9	1.819	0.01516
PP 2*PP 9	1.431	0.03707
PP 5*PP 8	1.373	0.04234
PP 6	1.355	0.04418 ^
PP 9*PP 9	1.199	0.06328
PP 1*PP 3	1.061	0.08698
PP 5*PP 5	0.970	0.10712
PP 4*PP 6	0.889	0.12921
PP 6*PP 8	0.832	0.14708

Lack Of Fit

Source	DF	Sum of Squares	Mean Square	F Ratio
Lack Of Fit	52	3.6547161	0.070283	0.8526
Pure Error	6	0.4945855	0.082431	**Prob > F**
Total Error	58	4.1493016		0.6649
				**Max RSq**
				0.9935

Residual by Predicted Plot

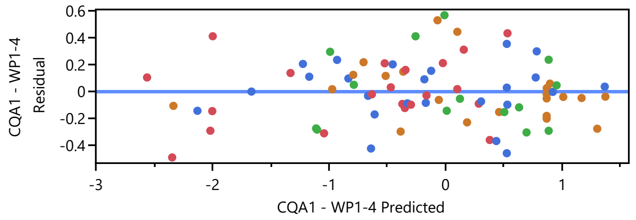

Studentized Residuals

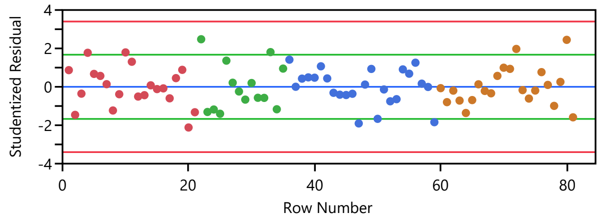

Externally studentized residuals with 90% simultaneous limits (Bonferroni) in red, individual limits in green.

Fit Group

Response CQA1 - WP1-4

Summary of Fit

RSquare	0.945419
RSquare Adj	0.924716
Root Mean Square Error	0.267469
Mean of Response	-0.17684
Observations (or Sum Wgts)	81

Analysis of Variance

Source	DF	Sum of Squares	Mean Square	F Ratio
Model	22	71.871973	3.26691	45.6657
Error	58	4.149302	0.07154	Prob > F
C. Total	80	76.021275		<.0001*

Parameter Estimates

Term	Estimate	Std Error	t Ratio	Prob>|t|
Intercept	-0.077003	0.106452	-0.72	0.4724
Workpackage[WP1]	-0.262991	0.068194	-3.86	0.0003*
Workpackage[WP2]	0.2739741	0.068373	4.01	0.0002*
Workpackage[WP3]	-0.085877	0.076394	-1.12	0.2656
PP 2	0.5497136	0.052807	10.41	<.0001*
PP 5	-0.742292	0.041561	-17.86	<.0001*
PP 6	-0.110092	0.053517	-2.06	0.0442*
PP 7	-0.275312	0.050391	-5.46	<.0001*
PP 9	0.7308954	0.096997	7.54	<.0001*
PP 2*PP 2	-0.241673	0.085404	-2.83	0.0064*
PP 1*PP 3	-0.131849	0.075729	-1.74	0.0870
PP 4*PP 4	-0.351295	0.09368	-3.75	0.0004*
PP 2*PP 5	0.2487631	0.062233	4.00	0.0002*
PP 5*PP 5	0.1694395	0.103528	1.64	0.1071
PP 2*PP 6	0.3503233	0.130339	2.69	0.0094*
PP 4*PP 6	-0.105973	0.068852	-1.54	0.1292
PP 5*PP 6	0.2790431	0.061576	4.53	<.0001*
PP 5*PP 8	0.1150685	0.05543	2.08	0.0423*
PP 6*PP 8	-0.088151	0.059984	-1.47	0.1471
PP 2*PP 9	-0.274259	0.12851	-2.13	0.0371*
PP 6*PP 9	-0.279154	0.111535	-2.50	0.0152*
PP 9*PP 9	-0.238015	0.125699	-1.89	0.0633
PP 1*PP 1*PP 1	0.3510653	0.061806	5.68	<.0001*

Residual by Row Plot

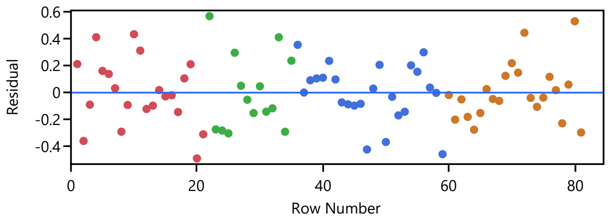

Prediction Profiler

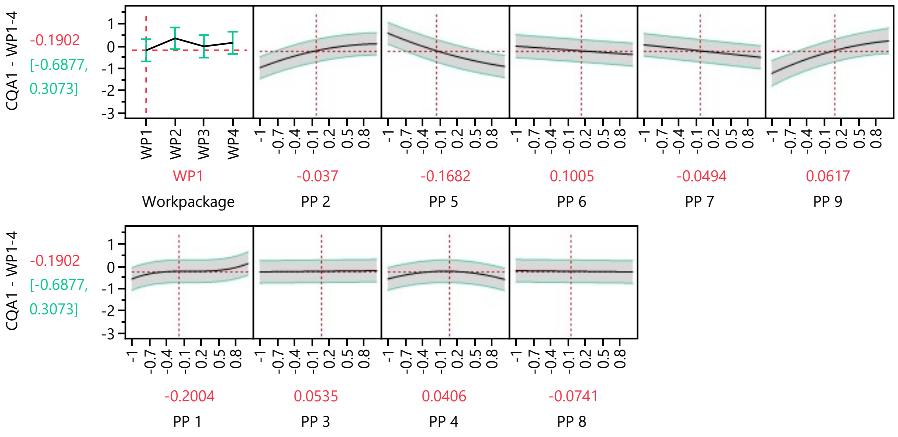

Fit Group

Response CQA1 - WP1-4

Residual Normal Quantile Plot

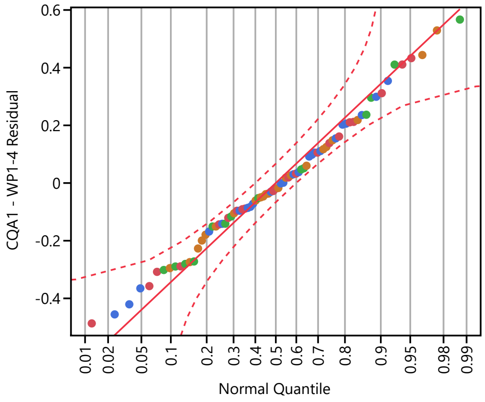

Press

Residual	SSE	RMSE	RSquare
Press	7.8468946185	0.31124788	0.8968
Ordinary	4.1493016093	0.26746903	0.9454

Response CQA2 - WP1-4

Actual by Predicted Plot

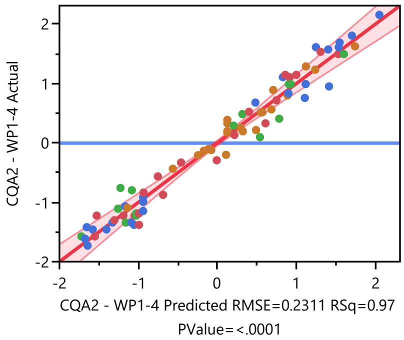

Fit Group

Response CQA2 - WP1-4

Effect Summary

Source	Logworth	PValue
PP 4*PP 4*PP 4	30.404	0.00000
Workpackage	11.804	0.00000
PP 4*PP 9	9.626	0.00000
PP 2	6.478	0.00000
PP 1*PP 9	6.390	0.00000
PP 8*PP 8	5.794	0.00000
PP 9*PP 9	5.720	0.00000
PP 2*PP 7	4.504	0.00003
PP 4*PP 4	4.456	0.00004 ^
PP 9	4.318	0.00005 ^
PP 5	4.211	0.00006
PP 6*PP 9	3.411	0.00039
PP 1	3.273	0.00053 ^
PP 3*PP 9	2.712	0.00194
PP 5*PP 9	2.478	0.00333
PP 2*PP 6	2.241	0.00574
PP 1*PP 6	2.231	0.00587
PP 1*PP 1	1.392	0.04053
PP 7	1.308	0.04920 ^
PP 4*PP 5	1.255	0.05562
PP 8	1.130	0.07411 ^

Lack Of Fit

Source	DF	Sum of Squares	Mean Square	F Ratio
Lack Of Fit	51	2.9837565	0.058505	5.8316
Pure Error	6	0.0601941	0.010032	**Prob > F**
Total Error	57	3.0439506		0.0167*
				**Max RSq**
				0.9994

Residual by Predicted Plot

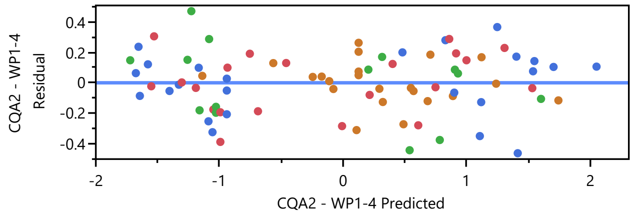

Studentized Residuals

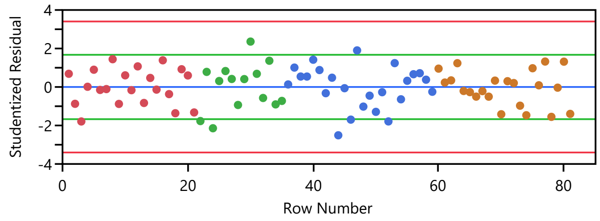

Externally studentized residuals with 90% simultaneous limits (Bonferroni) in red, individual limits in green.

Summary of Fit

RSquare	0.967157
RSquare Adj	0.953905
Root Mean Square Error	0.23109
Mean of Response	-0.00315
Observations (or Sum Wgts)	81

Analysis of Variance

Source	DF	Sum of Squares	Mean Square	F Ratio
Model	23	89.638504	3.89733	72.9800
Error	57	3.043951	0.05340	**Prob > F**
C. Total	80	92.682455		<.0001*

Fit Group

Response CQA2 - WP1-4

Residual by Row Plot

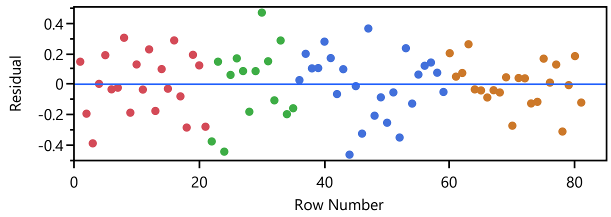

Prediction Profiler

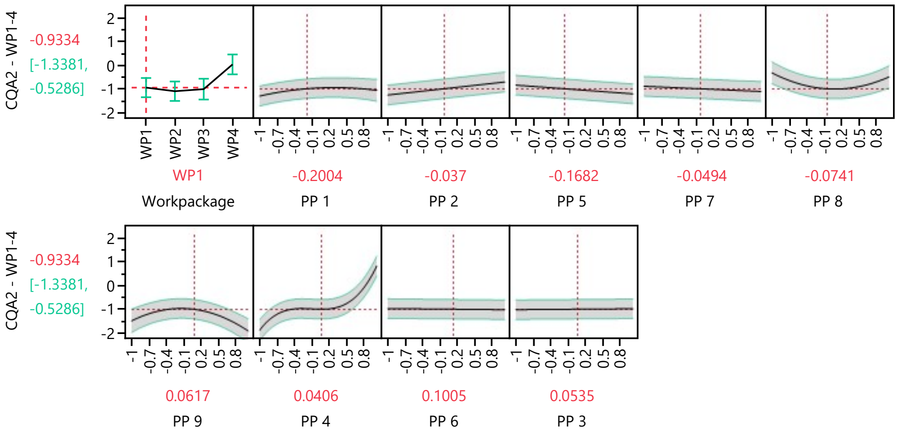

Residual Normal Quantile Plot

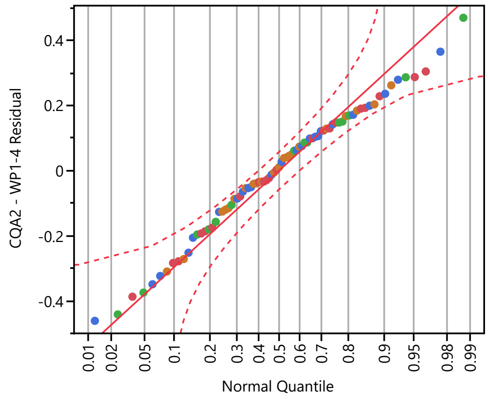

Press

Residual	SSE	RMSE	RSquare
Press	7.5979169739	0.30627021	0.9180
Ordinary	3.0439506385	0.23109012	0.9672

Response CQA3 - WP1-4

Actual by Predicted Plot

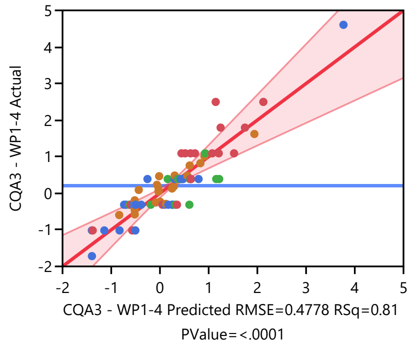

Fit Group

Response CQA3 - WP1-4

Effect Summary

Source	Logworth	PValue
PP 5	11.253	0.00000
PP 4*PP 5	6.430	0.00000
PP 8*PP 8	5.319	0.00000
PP 3*PP 7	5.086	0.00001
PP 8	5.056	0.00001 ^
PP 5*PP 9	5.023	0.00001
PP 9	4.376	0.00004 ^
PP 1*PP 9	3.902	0.00013
PP 4*PP 4	3.316	0.00048
PP 4*PP 6	3.137	0.00073
PP 7*PP 8	2.884	0.00131
PP 3*PP 8	2.579	0.00264
PP 2*PP 4	2.566	0.00272
PP 2	2.250	0.00562 ^
PP 6*PP 8	1.892	0.01283
PP 4*PP 7	1.854	0.01399
PP 1*PP 5	1.833	0.01467
PP 1*PP 2	1.811	0.01546
PP 5*PP 7	1.581	0.02624
PP 5*PP 6	1.398	0.04003
PP 6*PP 6*PP 6	1.390	0.04075
PP 2*PP 8	1.370	0.04266
PP 1*PP 7	1.277	0.05282
PP 2*PP 5	1.111	0.07749

Lack Of Fit

Source	DF	Sum of Squares	Mean Square	F Ratio
Lack Of Fit	49	12.108381	0.247110	2.5548
Pure Error	7	0.677075	0.096725	**Prob > F**
Total Error	56	12.785457		0.0971
				**Max RSq**
				0.9902

Residual by Predicted Plot

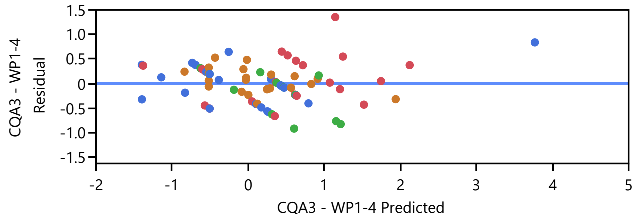

Studentized Residuals

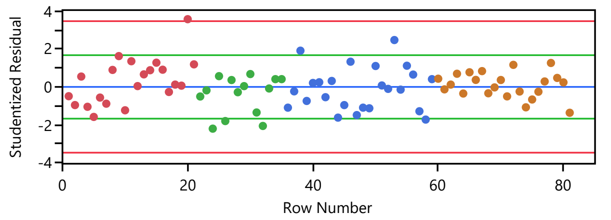

Externally studentized residuals with 90% simultaneous limits (Bonferroni) in red, individual limits in green.

Summary of Fit

RSquare	0.814556
RSquare Adj	0.73508
Root Mean Square Error	0.47782
Mean of Response	0.207098
Observations (or Sum Wgts)	81

Analysis of Variance

Source	DF	Sum of Squares	Mean Square	F Ratio
Model	24	56.159609	2.33998	10.2491
Error	56	12.785457	0.22831	**Prob > F**
C. Total	80	68.945065		<.0001*

Fit Group

Response CQA3 - WP1-4

Residual by Row Plot

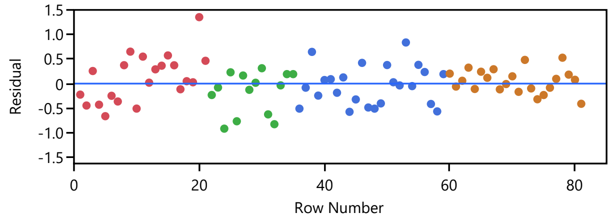

Prediction Profiler

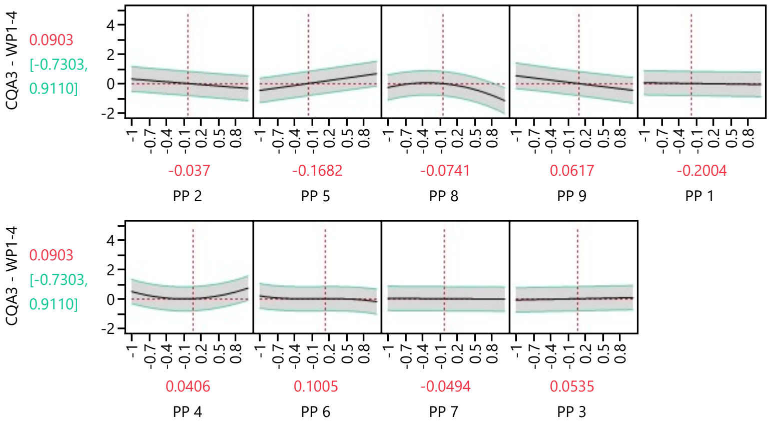

Residual Normal Quantile Plot

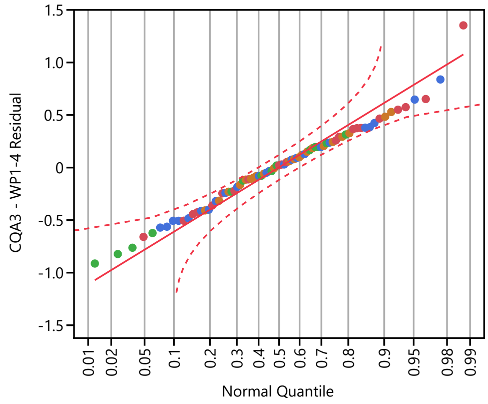

Press

Residual	SSE	RMSE	RSquare
Press	29.050792867	0.59887542	0.5786
Ordinary	12.785456548	0.47781976	0.8146

Graph Builder

Measured & Prediction vs. Experiment

Label = CQA1 - WP1-4

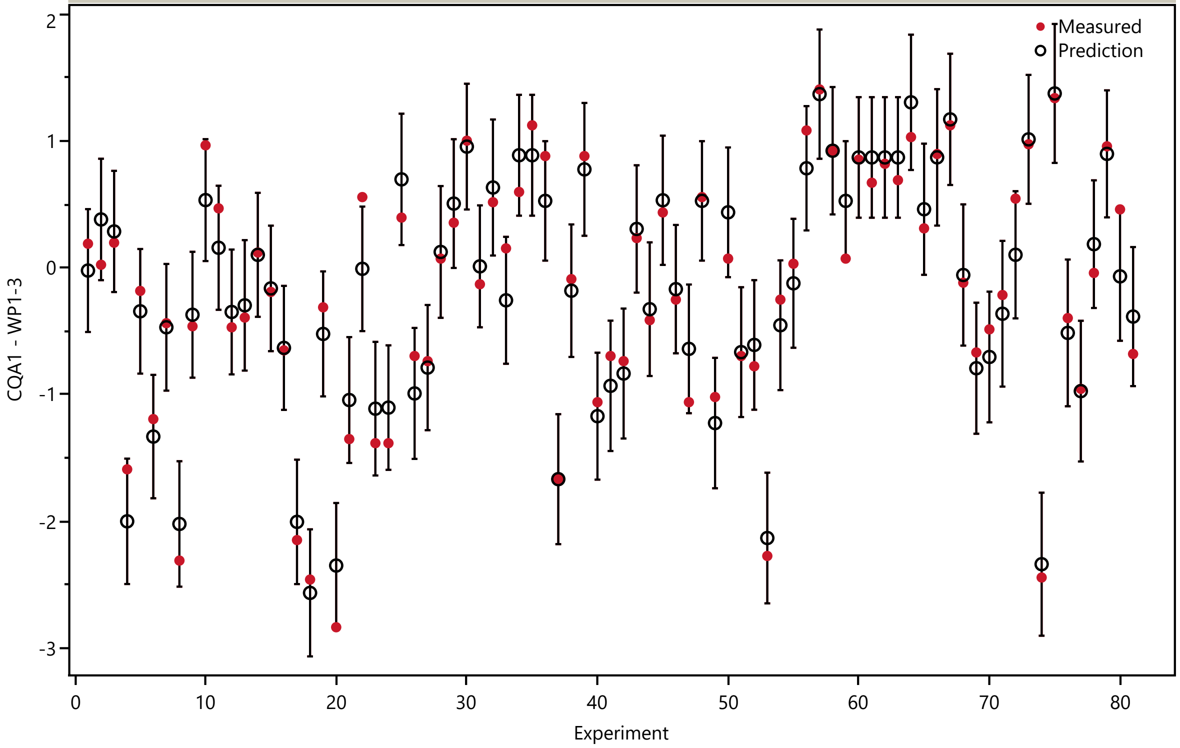

Label = CQA2 - WP1-4

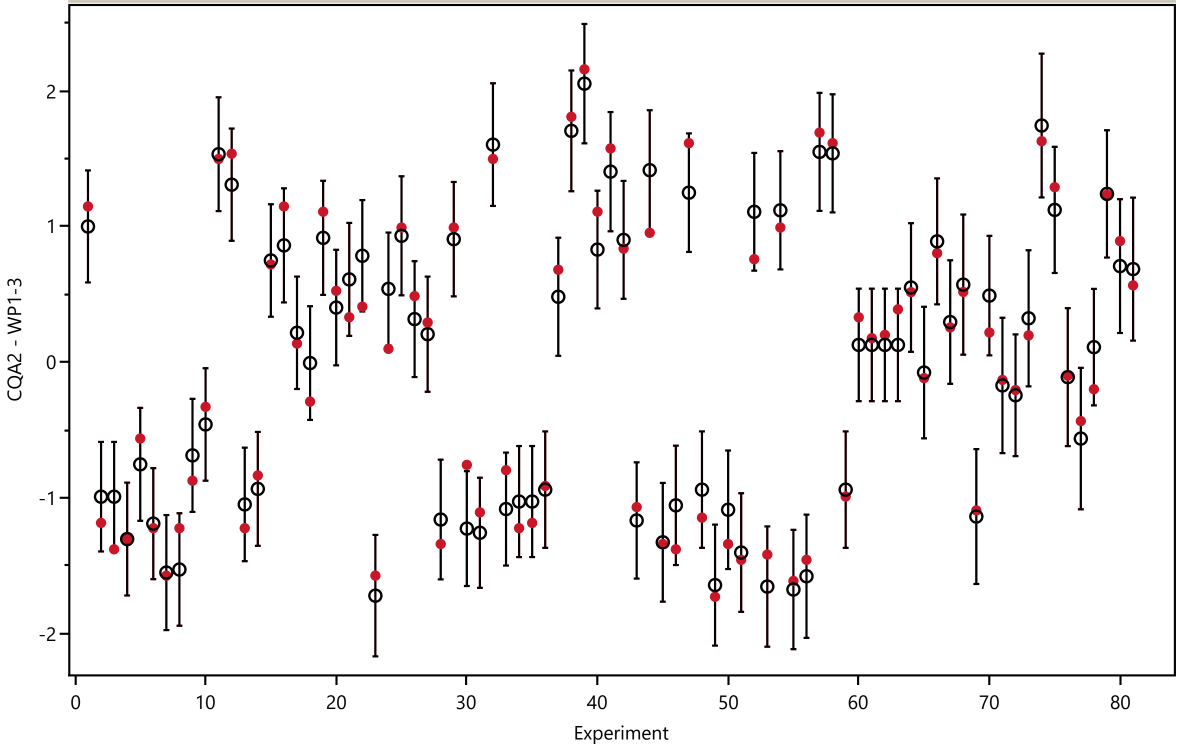

Graph Builder

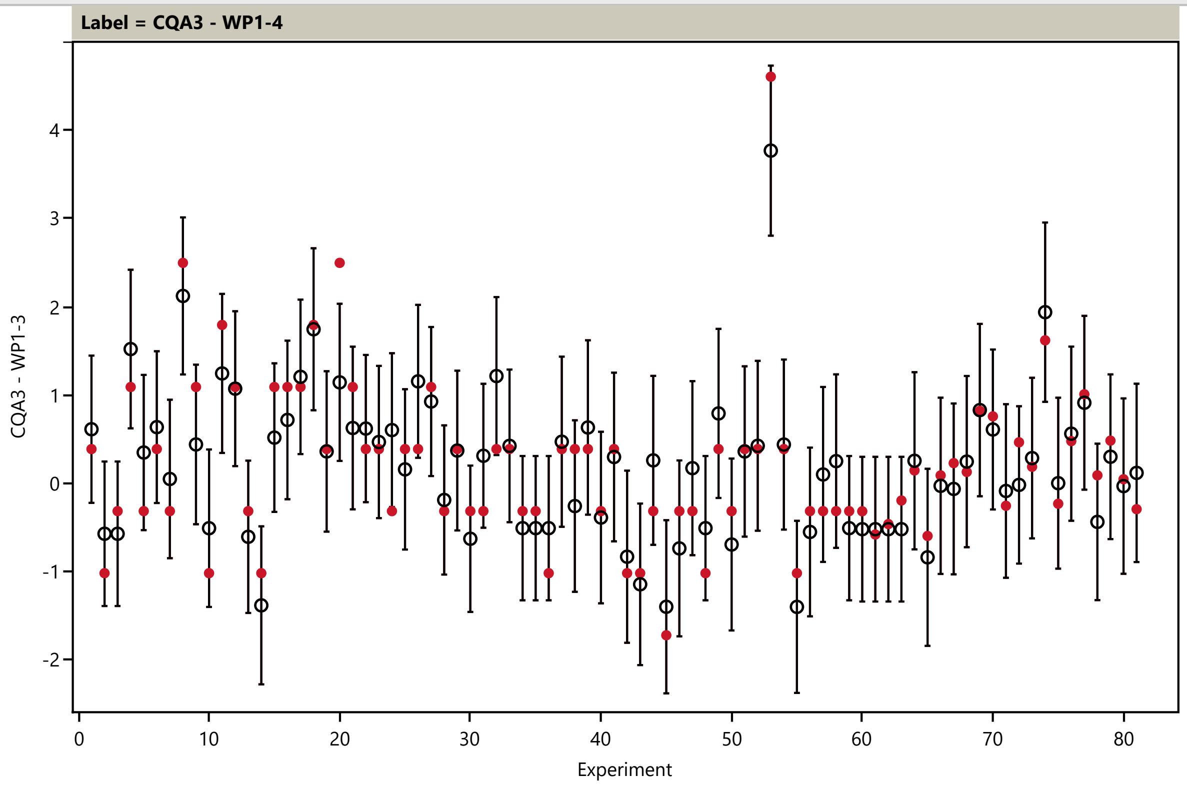

Each error bar is constructed from 90% lower PI to 90% upper PI.
