# Supplementary material for: Lifecycle DoE—The Companion for a Holistic Development Process
Source: Bioengineering (Basel). 2024 Oct 30;11(11):1089. doi: 10.3390/bioengineering11111089 (PMC11591819; doi:10.3390/bioengineering11111089)
Supplement: Supplementary file 1 [file bioengineering-11-01089-s001.zip › Supplementary_SE_WP1_7.pdf]

Fit Group

Response CQA1 - WP1-7

Actual by Predicted Plot

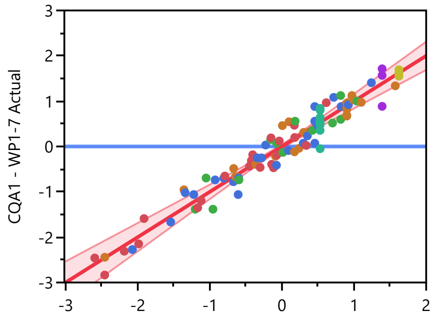

CQA1 - WP1-7 Predicted RMSE=0.2619 RSq=0.95 PValue=<.0001

Effect Summary

Source	Logworth	PValue
PP 5	28.349	0.00000
PP 2	16.758	0.00000
PP 9	12.527	0.00000
PP 1	11.978	0.00000
Workpackage	10.102	0.00000
PP 7	6.129	0.00000
PP 5*PP 6	5.003	0.00001
PP 4*PP 4	4.502	0.00003
PP 2*PP 5	4.436	0.00004
PP 2*PP 2	4.161	0.00007
PP 2*PP 6	3.761	0.00017
PP 9*PP 9	2.833	0.00147
PP 1*PP 3	2.562	0.00274
PP 4*PP 9	2.304	0.00497
PP 5*PP 8	1.232	0.05863
PP 6	1.041	0.09091 ^

Lack Of Fit

Source	DF	Sum of Squares	Mean Square	F Ratio
Lack Of Fit	56	3.6852164	0.065807	0.8572
Pure Error	19	1.4587090	0.076774	**Prob > F**
Total Error	75	5.1439255		0.6824
				**Max RSq**
				0.9848

Residual by Predicted Plot

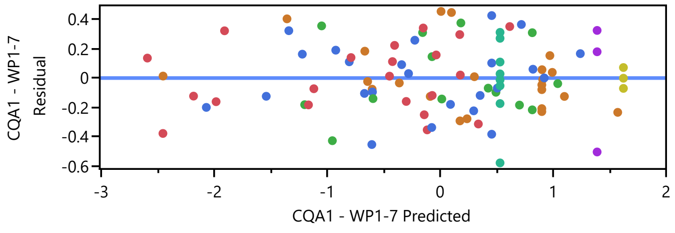

Studentized Residuals

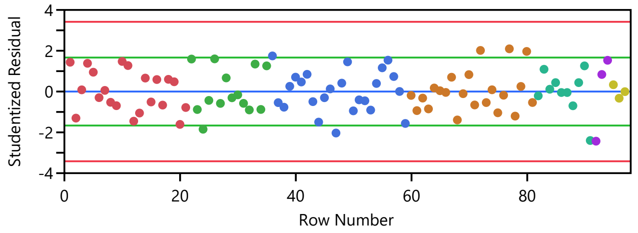

Externally studentized residuals with 90% simultaneous limits (Bonferroni) in red, individual limits in green.

Fit Group

Response CQA1 - WP1-7

Summary of Fit

RSquare	0.946417
RSquare Adj	0.931414
Root Mean Square Error	0.261889
Mean of Response	2.03e-15
Observations (or Sum Wgts)	97

Analysis of Variance

Source	DF	Sum of Squares	Mean Square	F Ratio
Model	21	90.856075	4.32648	63.0814
Error	75	5.143925	0.06859	Prob > F
C. Total	96	96.000000		<.0001*

Parameter Estimates

Term	Estimate	Std Error	t Ratio	Prob>|t|
Intercept	0.3220496	0.067074	4.80	<.0001*
Workpackage[WP1]	-0.428931	0.083373	-5.14	<.0001*
Workpackage[WP2]	0.0638146	0.076796	0.83	0.4086
Workpackage[WP3]	-0.29568	0.082039	-3.60	0.0006*
Workpackage[WP4]	-0.045475	0.086767	-0.52	0.6018
Workpackage[WP5]	-0.415558	0.086049	-4.83	<.0001*
Workpackage[WP6]	0.4443656	0.137242	3.24	0.0018*
PP 1	0.3963881	0.046339	8.55	<.0001*
PP 2	0.5285671	0.047634	11.10	<.0001*
PP 5	-0.725648	0.04011	-18.09	<.0001*
PP 6	-0.087446	0.05106	-1.71	0.0909
PP 7	-0.262609	0.048601	-5.40	<.0001*
PP 9	0.807108	0.091279	8.84	<.0001*
PP 2*PP 2	-0.351689	0.083446	-4.21	<.0001*
PP 1*PP 3	-0.222558	0.071844	-3.10	0.0027*
PP 4*PP 4	-0.402626	0.090865	-4.43	<.0001*
PP 2*PP 5	0.2654514	0.060473	4.39	<.0001*
PP 2*PP 6	0.4755188	0.12031	3.95	0.0002*
PP 5*PP 6	0.2771884	0.058482	4.74	<.0001*
PP 5*PP 8	0.1028013	0.053535	1.92	0.0586
PP 4*PP 9	-0.406068	0.140274	-2.89	0.0050*
PP 9*PP 9	-0.409393	0.123944	-3.30	0.0015*

Residual by Row Plot

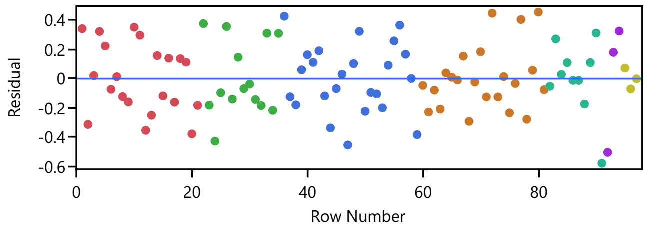

Prediction Profiler

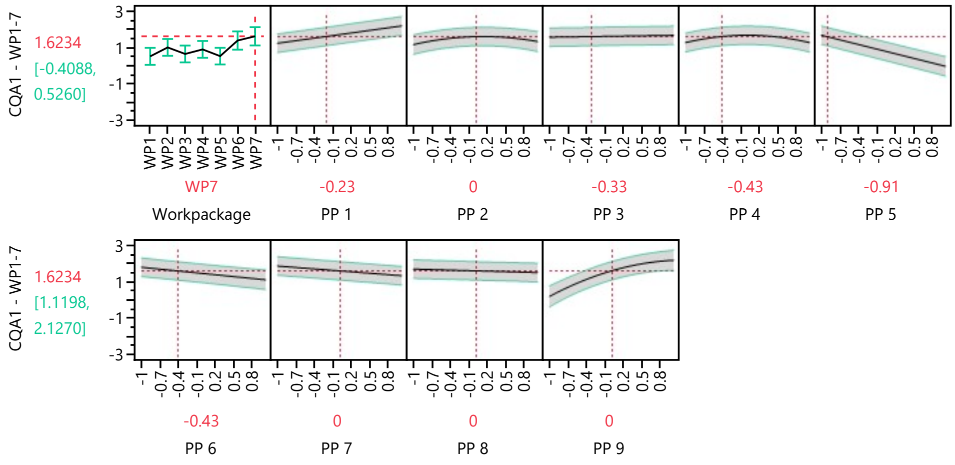

Fit Group

Response CQA1 - WP1-7

Residual Normal Quantile Plot

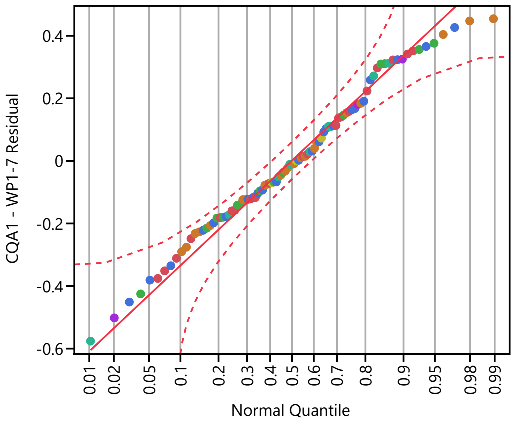

Press

Residual	SSE	RMSE	RSquare
Press	8.6364757714	0.29838873	0.9100
Ordinary	5.1439254804	0.26188867	0.9464

Response CQA2 - WP1-7

Actual by Predicted Plot

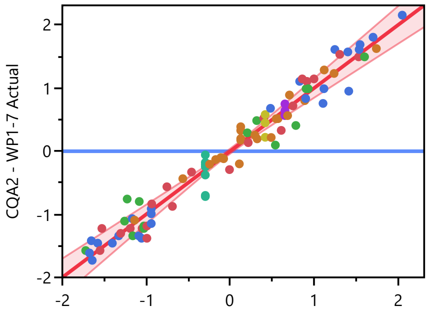

CQA2 - WP1-7 Predicted RMSE=0.2294 RSq=0.96
PValue=<.0001

Fit Group

Response CQA2 - WP1-7

Effect Summary

Source	Logworth	PValue
PP 4*PP 4*PP 4	34.556	0.00000
Workpackage	18.415	0.00000
PP 4*PP 9	10.250	0.00000
PP 2	6.790	0.00000
PP 1*PP 9	6.694	0.00000
PP 8*PP 8	6.050	0.00000
PP 9*PP 9	5.971	0.00000
PP 2*PP 7	4.670	0.00002
PP 4*PP 4	4.618	0.00002 ^
PP 9	4.472	0.00003 ^
PP 5	4.359	0.00004
PP 6*PP 9	3.514	0.00031
PP 1	3.369	0.00043 ^
PP 3*PP 9	2.782	0.00165
PP 5*PP 9	2.539	0.00289
PP 2*PP 6	2.293	0.00510
PP 1*PP 6	2.283	0.00522
PP 1*PP 1	1.417	0.03829
PP 7	1.331	0.04671 ^
PP 4*PP 5	1.276	0.05297
PP 8	1.148	0.07106 ^

Lack Of Fit

Source	DF	Sum of Squares	Mean Square	F Ratio
Lack Of Fit	51	2.9837565	0.058505	1.5896
Pure Error	19	0.6992821	0.036804	**Prob > F**
Total Error	70	3.6830386		0.1342
				**Max RSq**
				0.9927

Residual by Predicted Plot

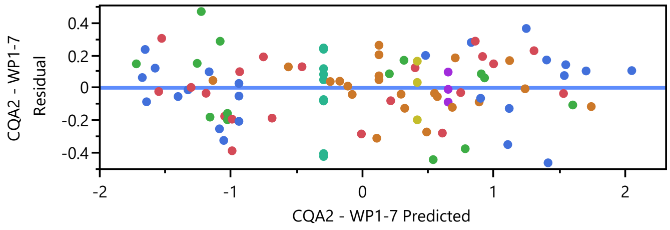

Studentized Residuals

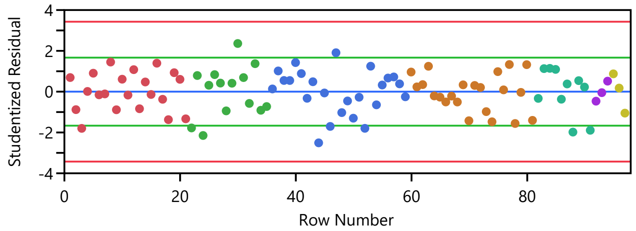

Externally studentized residuals with 90% simultaneous limits (Bonferroni) in red, individual limits in green.

Summary of Fit

RSquare	0.961635
RSquare Adj	0.947385
Root Mean Square Error	0.229379
Mean of Response	9.94e-16
Observations (or Sum Wgts)	97

Analysis of Variance

Source	DF	Sum of Squares	Mean Square	F Ratio
Model	26	92.316961	3.55065	67.4839
Error	70	3.683039	0.05261	**Prob > F**
C. Total	96	96.000000		<.0001*

Fit Group

Response CQA2 - WP1-7

Parameter Estimates

Term	Estimate	Std Error	t Ratio	Prob>|t|
Intercept	-0.315653	0.05746	-5.49	<.0001*
Workpackage[WP1]	-0.584221	0.066568	-8.78	<.0001*
Workpackage[WP2]	-0.735602	0.071128	-10.34	<.0001*
Workpackage[WP3]	-0.647929	0.094323	-6.87	<.0001*
Workpackage[WP4]	0.3928192	0.084283	4.66	<.0001*
Workpackage[WP5]	-0.029141	0.074712	-0.39	0.6977
Workpackage[WP6]	0.9195685	0.119796	7.68	<.0001*
PP 1	0.1817133	0.049132	3.70	0.0004*
PP 2	0.2581222	0.044349	5.82	<.0001*
PP 5	-0.15398	0.035316	-4.36	<.0001*
PP 7	-0.089667	0.044286	-2.02	0.0467*
PP 8	-0.084221	0.045948	-1.83	0.0711
PP 9	-0.553278	0.12483	-4.43	<.0001*
PP 1*PP 1	-0.216734	0.102638	-2.11	0.0383*
PP 4*PP 4	0.4616936	0.10203	4.53	<.0001*
PP 4*PP 5	-0.08056	0.040924	-1.97	0.0530
PP 1*PP 6	0.1950148	0.067625	2.88	0.0052*
PP 2*PP 6	0.3507959	0.121302	2.89	0.0051*
PP 2*PP 7	0.4914279	0.10783	4.56	<.0001*
PP 8*PP 8	0.5850618	0.108498	5.39	<.0001*
PP 1*PP 9	-1.345279	0.233331	-5.77	<.0001*
PP 3*PP 9	0.2890275	0.088292	3.27	0.0017*
PP 4*PP 9	-1.360533	0.175899	-7.73	<.0001*
PP 5*PP 9	-0.384583	0.124556	-3.09	0.0029*
PP 6*PP 9	0.4810908	0.126615	3.80	0.0003*
PP 9*PP 9	-0.708427	0.13252	-5.35	<.0001*
PP 4*PP 4*PP 4	1.3996537	0.058797	23.80	<.0001*

Residual by Row Plot

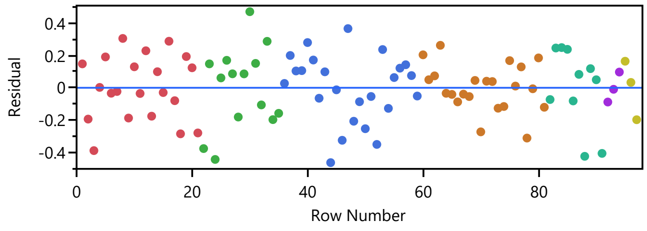

Prediction Profiler

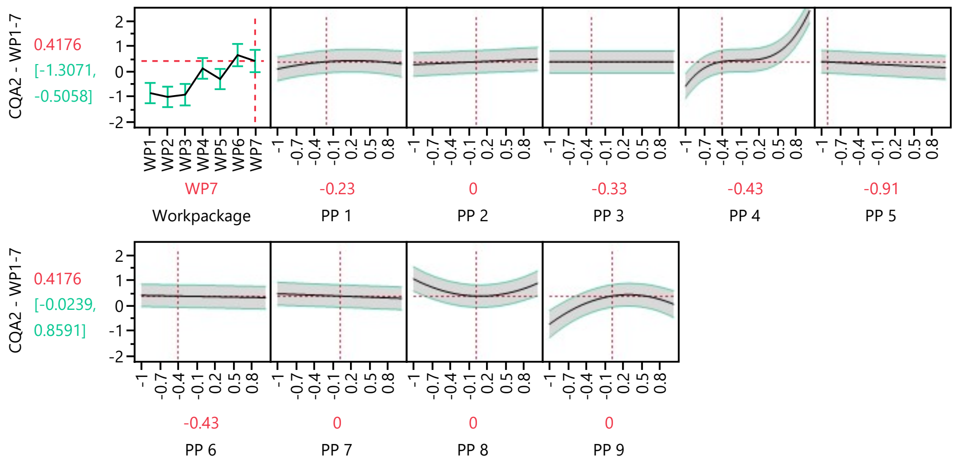

Fit Group

Response CQA2 - WP1-7

Residual Normal Quantile Plot

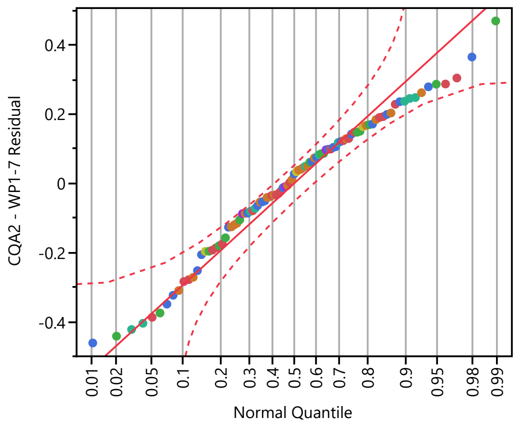

Press

Residual	SSE	RMSE	RSquare
Press	8.4721031444	0.29553556	0.9117
Ordinary	3.6830386394	0.22937924	0.9616

Response CQA3 - WP1-7

Actual by Predicted Plot

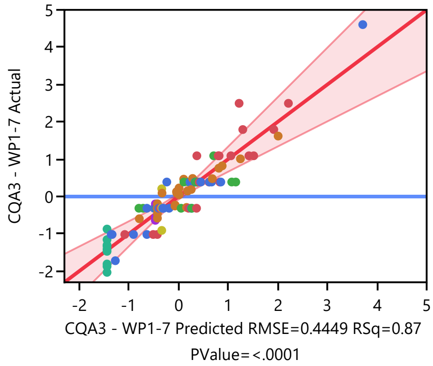

Fit Group

Response CQA3 - WP1-7

Effect Summary

Source	Logworth	PValue
PP 5	11.377	0.00000
PP 4*PP 5	7.420	0.00000
PP 8	5.089	0.00001
PP 9	4.977	0.00001
Workpackage	4.747	0.00002
PP 5*PP 9	4.562	0.00003
PP 4*PP 4	4.106	0.00008
PP 3*PP 7	4.024	0.00009
PP 1*PP 9	3.394	0.00040
PP 7*PP 8	2.974	0.00106
PP 8*PP 8	2.864	0.00137
PP 2*PP 4	2.625	0.00237
PP 2	2.427	0.00374 ^
PP 3*PP 8	2.224	0.00597
PP 6*PP 8	2.130	0.00742
PP 4*PP 7	2.009	0.00979
PP 1*PP 5	1.781	0.01655
PP 1*PP 2	1.720	0.01905
PP 5*PP 7	1.624	0.02375
PP 2*PP 5	1.460	0.03465
PP 2*PP 8	1.459	0.03476
PP 1*PP 6	1.454	0.03512
PP 4*PP 6	1.376	0.04206
PP 1	1.361	0.04351 ^
PP 5*PP 6	1.346	0.04509
PP 1*PP 7	1.343	0.04537

Lack Of Fit

Source	DF	Sum of Squares	Mean Square	F Ratio
Lack Of Fit	46	10.519561	0.228686	1.8522
Pure Error	19	2.345918	0.123469	**Prob > F**
Total Error	65	12.865478		0.0724
				**Max RSq**
				0.9756

Residual by Predicted Plot

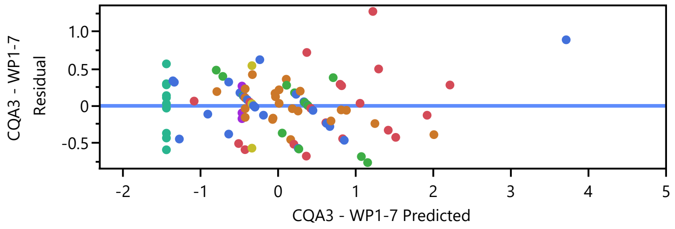

Studentized Residuals

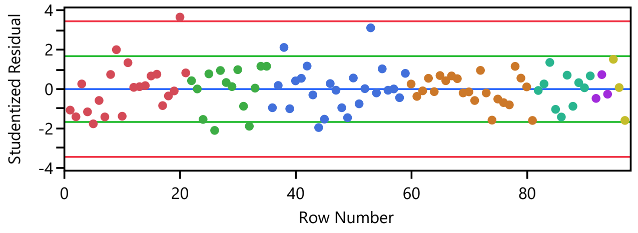

Externally studentized residuals with 90% simultaneous limits (Bonferroni) in red, individual limits in green.

Summary of Fit

RSquare	0.865985
RSquare Adj	0.80207
Root Mean Square Error	0.444894
Mean of Response	-9e-16
Observations (or Sum Wgts)	97

Fit Group

Response CQA3 - WP1-7

Analysis of Variance

Source	DF	Sum of Squares	Mean Square	F Ratio
Model	31	83.134522	2.68176	13.5490
Error	65	12.865478	0.19793	Prob > F
C. Total	96	96.000000		<.0001*

Parameter Estimates

Term	Estimate	Std Error	t Ratio	Prob>|t|
Intercept	-0.023384	0.115804	-0.20	0.8406
Workpackage[WP1]	0.2134095	0.129419	1.65	0.1040
Workpackage[WP2]	-0.166371	0.141078	-1.18	0.2426
Workpackage[WP3]	-0.005734	0.166394	-0.03	0.9726
Workpackage[WP4]	0.2319459	0.139662	1.66	0.1016
Workpackage[WP5]	-0.782765	0.14511	-5.39	<.0001*
Workpackage[WP6]	0.1905544	0.232476	0.82	0.4154
PP 1	-0.193327	0.093894	-2.06	0.0435*
PP 2	-0.314296	0.104493	-3.01	0.0037*
PP 5	0.6011244	0.070931	8.47	<.0001*
PP 8	-0.459176	0.094742	-4.85	<.0001*
PP 9	-0.959325	0.200842	-4.78	<.0001*
PP 1*PP 2	0.3852651	0.160225	2.40	0.0191*
PP 2*PP 4	0.382189	0.120802	3.16	0.0024*
PP 4*PP 4	0.6926965	0.164291	4.22	<.0001*
PP 1*PP 5	-0.209662	0.085222	-2.46	0.0166*
PP 2*PP 5	-0.242967	0.112602	-2.16	0.0346*
PP 4*PP 5	-0.497096	0.079728	-6.23	<.0001*
PP 1*PP 6	0.219666	0.10208	2.15	0.0351*
PP 4*PP 6	0.2295266	0.110679	2.07	0.0421*
PP 5*PP 6	-0.212891	0.104194	-2.04	0.0451*
PP 1*PP 7	-0.201846	0.098923	-2.04	0.0454*
PP 3*PP 7	-0.699491	0.168077	-4.16	<.0001*
PP 4*PP 7	-0.2637	0.099072	-2.66	0.0098*
PP 5*PP 7	0.2172038	0.093805	2.32	0.0238*
PP 2*PP 8	-0.457973	0.212379	-2.16	0.0348*
PP 3*PP 8	-0.476779	0.167706	-2.84	0.0060*
PP 6*PP 8	0.2988222	0.108105	2.76	0.0074*
PP 7*PP 8	-0.333308	0.097253	-3.43	0.0011*
PP 8*PP 8	-0.687638	0.205521	-3.35	0.0014*
PP 1*PP 9	-1.280746	0.343302	-3.73	0.0004*
PP 5*PP 9	-0.94083	0.208455	-4.51	<.0001*

Residual by Row Plot

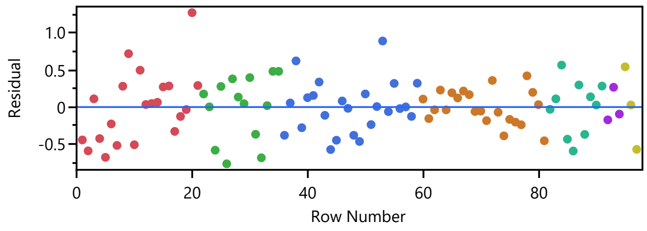

Prediction Profiler

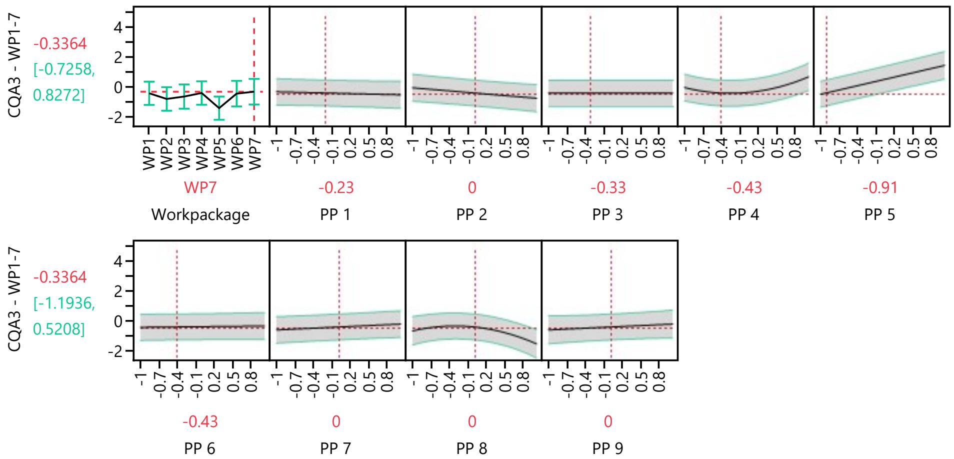

Fit Group

Response CQA3 - WP1-7

Residual Normal Quantile Plot

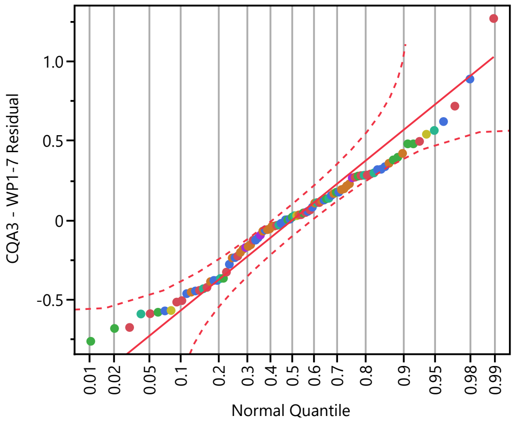

Press

Residual	SSE	RMSE	RSquare
Press	32.414254779	0.57807229	0.6624
Ordinary	12.865478117	0.44489373	0.8660

Fit Group

Mixed Model for CQA1 - WP1-7

Actual by Predicted Plot

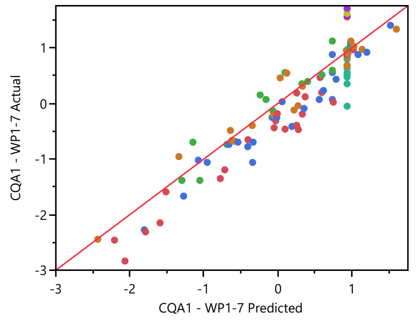

Actual by Conditional Predicted Plot

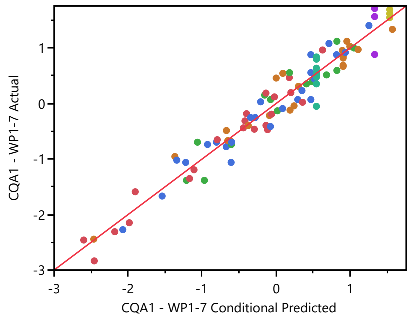

Fit Statistics

Number of rows	97
Sum of Frequencies	97
-2 Log Likelihood	22.408915
AICc	67.178146
BIC	104.75371

Random Effects Covariance Parameter Estimates

Variance Component	Var Ratio	Estimate	Std Error	90% Lower	90% Upper	Pct of Total
Workpackage	2.34736	0.1613999	0.1031479	0.0724398	0.7199408	70.126
Residual		0.0687579	0.0112566	0.0535648	0.0920714	29.874
Total		0.2301578	0.1033565	0.1254633	0.586966	100.000

Fixed Effects Parameter Estimates

Indicator Coding

Term	Estimate	Std Error	DFDen	t Ratio	Prob>|t|	90% Lower	90% Upper
Intercept	0.3084622	0.1660004	7.0	1.86	0.1057	-0.006256	0.62318
PP 1	0.3968435	0.0463807	75.2	8.56	<.0001*	0.3196026	0.4740844
PP 2	0.5285385	0.0476926	74.7	11.08	<.0001*	0.4491055	0.6079715
PP 5	-0.729477	0.0401332	75.4	-18.18	<.0001*	-0.796312	-0.662642
PP 6	-0.092519	0.0510848	75.5	-1.81	0.0741	-0.17759	-0.007447
PP 7	-0.262115	0.0486603	74.7	-5.39	<.0001*	-0.343159	-0.18107
PP 9	0.8115427	0.0913737	74.9	8.88	<.0001*	0.6593635	0.963722
PP 2*PP 2	-0.361496	0.0833643	77.6	-4.34	<.0001*	-0.500275	-0.222717
PP 1*PP 3	-0.219198	0.0719182	74.9	-3.05	0.0032*	-0.338974	-0.099421
PP 4*PP 4	-0.412794	0.0908387	76.7	-4.54	<.0001*	-0.564038	-0.26155
PP 2*PP 5	0.2646119	0.0605464	74.7	4.37	<.0001*	0.1637712	0.3654527
PP 2*PP 6	0.4754029	0.1204528	74.7	3.95	0.0002*	0.2747887	0.6760171
PP 5*PP 6	0.2826453	0.0585143	75.4	4.83	<.0001*	0.185201	0.3800895
PP 5*PP 8	0.1027451	0.0535965	74.8	1.92	0.0591	0.0134814	0.1920089
PP 4*PP 9	-0.407199	0.1404457	74.7	-2.90	0.0049*	-0.641113	-0.173284
PP 9*PP 9	-0.40424	0.1238238	77.8	-3.26	0.0016*	-0.610366	-0.198115

-2 Residual Log Likelihood 78.165798

Effect Coding

Term	Estimate	Std Error	DFDen	t Ratio	Prob>|t|	90% Lower	90% Upper
Intercept	0.3084622	0.1660004	7.0	1.86	0.1057	-0.006256	0.62318
PP 1	0.3968435	0.0463807	75.2	8.56	<.0001*	0.3196026	0.4740844
PP 2	0.5285385	0.0476926	74.7	11.08	<.0001*	0.4491055	0.6079715
PP 5	-0.729477	0.0401332	75.4	-18.18	<.0001*	-0.796312	-0.662642
PP 6	-0.092519	0.0510848	75.5	-1.81	0.0741	-0.17759	-0.007447
PP 7	-0.262115	0.0486603	74.7	-5.39	<.0001*	-0.343159	-0.18107
PP 9	0.8115427	0.0913737	74.9	8.88	<.0001*	0.6593635	0.963722
PP 2*PP 2	-0.361496	0.0833643	77.6	-4.34	<.0001*	-0.500275	-0.222717
PP 1*PP 3	-0.219198	0.0719182	74.9	-3.05	0.0032*	-0.338974	-0.099421
PP 4*PP 4	-0.412794	0.0908387	76.7	-4.54	<.0001*	-0.564038	-0.26155
PP 2*PP 5	0.2646119	0.0605464	74.7	4.37	<.0001*	0.1637712	0.3654527
PP 2*PP 6	0.4754029	0.1204528	74.7	3.95	0.0002*	0.2747887	0.6760171
PP 5*PP 6	0.2826453	0.0585143	75.4	4.83	<.0001*	0.185201	0.3800895
PP 5*PP 8	0.1027451	0.0535965	74.8	1.92	0.0591	0.0134814	0.1920089
PP 4*PP 9	-0.407199	0.1404457	74.7	-2.90	0.0049*	-0.641113	-0.173284
PP 9*PP 9	-0.40424	0.1238238	77.8	-3.26	0.0016*	-0.610366	-0.198115

-2 Residual Log Likelihood 78.165798

Random Coefficients

Workpackage

Workpackage	Intercept
WP1	-0.39221
WP2	0.0850679
WP3	-0.266763
WP4	-0.030236
WP5	-0.391365
WP6	0.395696
WP7	0.5998089

Covariance Matrix

Random Effect	Intercept
Intercept	0.1614

Fit Group

Mixed Model for CQA1 - WP1-7

Fixed Effects Tests

Source	Nparm	DFNum	DFDen	F Ratio	Prob > F
PP 1	1	1	75.2	73.208789	<.0001*
PP 2	1	1	74.7	122.81509	<.0001*
PP 5	1	1	75.4	330.38112	<.0001*
PP 6	1	1	75.5	3.2800137	0.0741
PP 7	1	1	74.7	29.015655	<.0001*
PP 9	1	1	74.9	78.882361	<.0001*
PP 2*PP 2	1	1	77.6	18.803847	<.0001*
PP 1*PP 3	1	1	74.9	9.2895408	0.0032*
PP 4*PP 4	1	1	76.7	20.650223	<.0001*
PP 2*PP 5	1	1	74.7	19.100361	<.0001*
PP 2*PP 6	1	1	74.7	15.577207	0.0002*
PP 5*PP 6	1	1	75.4	23.332397	<.0001*
PP 5*PP 8	1	1	74.8	3.6749426	0.0591
PP 4*PP 9	1	1	74.7	8.4061283	0.0049*
PP 9*PP 9	1	1	77.8	10.657887	0.0016*

Residual Plots

Residual by Predicted Plot

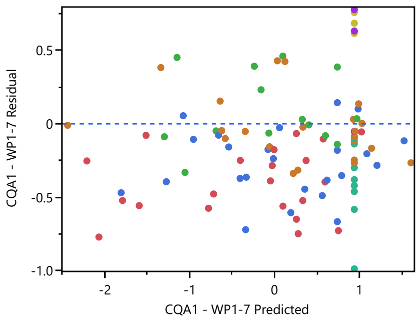

Residual Quantile Plot

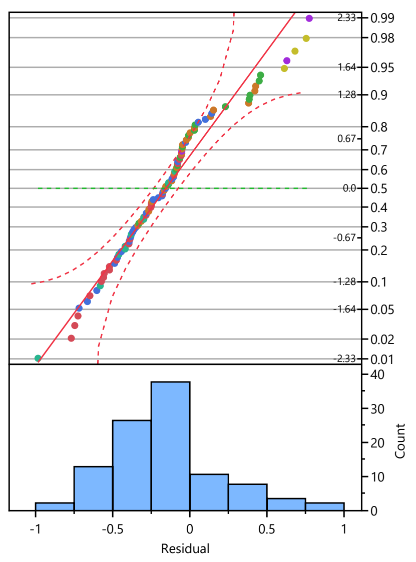

Residual by Row Plot

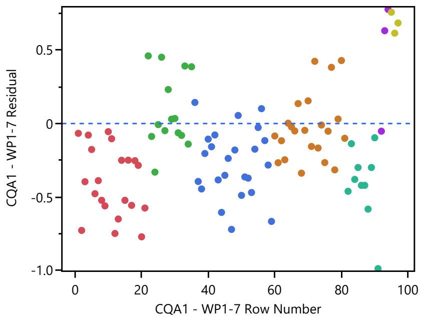

Fit Group

Mixed Model for CQA1 - WP1-7

Conditional Residual Plots

Conditional Residual by Predicted Plot

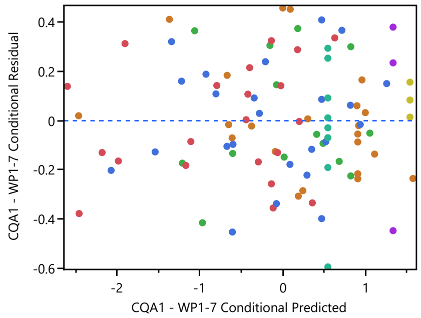

Conditional Residual Quantile Plot

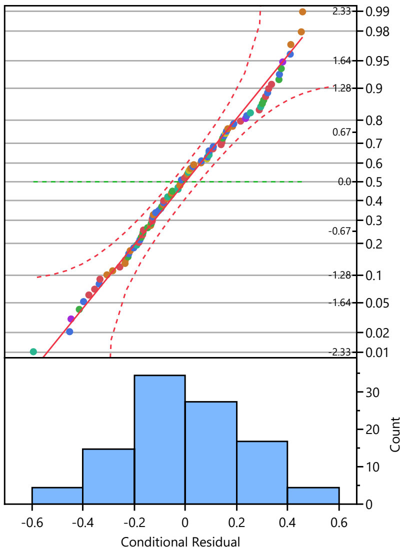

Conditional Residual by Row Plot

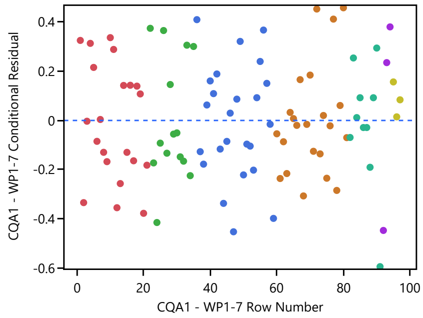

Marginal Model Profiler

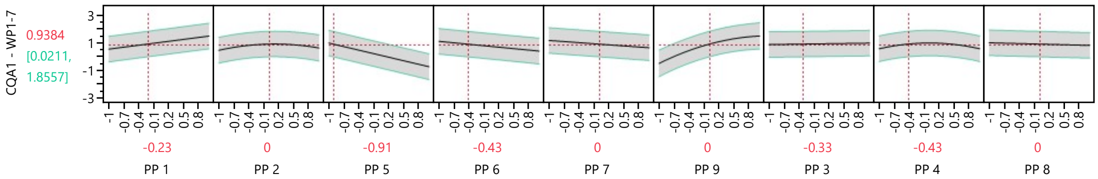

Mixed Model for CQA2 - WP1-7

Actual by Predicted Plot

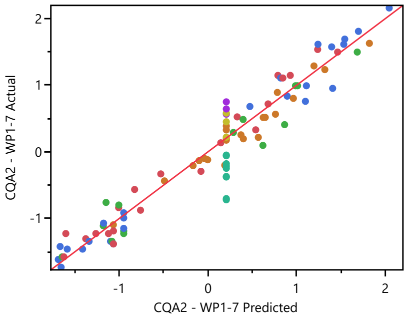

Actual by Conditional Predicted Plot

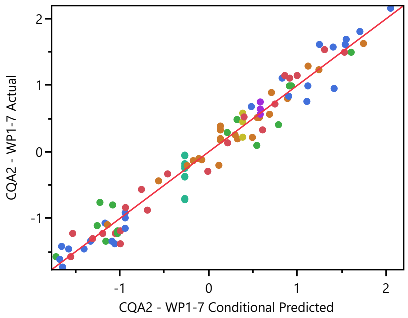

Fit Statistics

Number of rows	97
Sum of Frequencies	97
-2 Log Likelihood	-11.48039
AICc	53.186278
BIC	98.312674

Random Effects Covariance Parameter Estimates

Variance Component	Var Ratio	Estimate	Std Error	90% Lower	90% Upper	Pct of Total
Workpackage	1.73023	0.0911021	0.0641909	0.0384946	0.5081911	63.373
Residual		0.0526533	0.0089067	0.040705	0.0712542	36.627
Total		0.1437554	0.0645402	0.0783733	0.3665101	100.000

Fit Group

Mixed Model for CQA2 - WP1-7

Fixed Effects Parameter Estimates

Indicator Coding

Term	Estimate	Std Error	DFDen	t Ratio	Prob>|t|	90% Lower	90% Upper
Intercept	0.1582075	0.1664653	5.9	0.95	0.3793	-0.166439	0.4828542
Analytical Method[A-B]	-1.129535	0.2452903	5.1	-4.60	0.0054*	-1.620903	-0.638167
PP 1	0.1824493	0.0491352	70.9	3.71	0.0004*	0.1005583	0.2643403
PP 2	0.258258	0.0443643	70.0	5.82	<.0001*	0.1843065	0.3322095
PP 5	-0.153996	0.035323	70.5	-4.36	<.0001*	-0.21287	-0.095121
PP 7	-0.089344	0.0443008	70.0	-2.02	0.0476*	-0.16319	-0.015498
PP 8	-0.083775	0.0459624	70.1	-1.82	0.0726	-0.160389	-0.00716
PP 9	-0.551804	0.1248424	70.4	-4.42	<.0001*	-0.759888	-0.343719
PP 1*PP 1	-0.215259	0.1026626	70.3	-2.10	0.0396*	-0.386378	-0.04414
PP 4*PP 4	0.4623322	0.1020571	70.2	4.53	<.0001*	0.2922198	0.6324446
PP 4*PP 5	-0.080501	0.0409329	70.2	-1.97	0.0532	-0.14873	-0.012273
PP 1*PP 6	0.1941715	0.067644	70.2	2.87	0.0054*	0.0814193	0.3069236
PP 2*PP 6	0.350659	0.1213383	70.1	2.89	0.0051*	0.1484042	0.5529137
PP 2*PP 7	0.4919883	0.107857	70.3	4.56	<.0001*	0.3122091	0.6717675
PP 8*PP 8	0.5822485	0.1082691	74.1	5.38	<.0001*	0.4019056	0.7625913
PP 1*PP 9	-1.343088	0.2333313	70.6	-5.76	<.0001*	-1.731984	-0.954191
PP 3*PP 9	0.2891627	0.0883237	69.9	3.27	0.0017*	0.141932	0.4363935
PP 4*PP 9	-1.358422	0.1759146	70.4	-7.72	<.0001*	-1.651632	-1.065212
PP 5*PP 9	-0.38516	0.124599	69.9	-3.09	0.0029*	-0.592858	-0.177462
PP 6*PP 9	0.4807861	0.1266505	70.1	3.80	0.0003*	0.2696745	0.6918977
PP 9*PP 9	-0.712343	0.1324882	71.9	-5.38	<.0001*	-0.933112	-0.491574
PP 4*PP 4*PP 4	1.3990657	0.0588033	70.6	23.79	<.0001*	1.3010575	1.4970738

-2 Residual Log Likelihood	60.83275

Effect Coding

Term	Estimate	Std Error	DFDen	t Ratio	Prob>|t|	90% Lower	90% Upper
Analytical Method[A]	-0.564768	0.1226452	5.1	-4.60	0.0054*	-0.810452	-0.319084
Intercept	-0.40656	0.1286873	6.2	-3.16	0.0189*	-0.655417	-0.157703
PP 1	0.1824493	0.0491352	70.9	3.71	0.0004*	0.1005583	0.2643403
PP 1*PP 1	-0.215259	0.1026626	70.3	-2.10	0.0396*	-0.386378	-0.04414
PP 1*PP 6	0.1941715	0.067644	70.2	2.87	0.0054*	0.0814193	0.3069236
PP 1*PP 9	-1.343088	0.2333313	70.6	-5.76	<.0001*	-1.731984	-0.954191
PP 2	0.258258	0.0443643	70.0	5.82	<.0001*	0.1843065	0.3322095
PP 2*PP 6	0.350659	0.1213383	70.1	2.89	0.0051*	0.1484042	0.5529137
PP 2*PP 7	0.4919883	0.107857	70.3	4.56	<.0001*	0.3122091	0.6717675
PP 3*PP 9	0.2891627	0.0883237	69.9	3.27	0.0017*	0.141932	0.4363935
PP 4*PP 4	0.4623322	0.1020571	70.2	4.53	<.0001*	0.2922198	0.6324446
PP 4*PP 4*PP 4	1.3990657	0.0588033	70.6	23.79	<.0001*	1.3010575	1.4970738
PP 4*PP 5	-0.080501	0.0409329	70.2	-1.97	0.0532	-0.14873	-0.012273
PP 4*PP 9	-1.358422	0.1759146	70.4	-7.72	<.0001*	-1.651632	-1.065212
PP 5	-0.153996	0.035323	70.5	-4.36	<.0001*	-0.21287	-0.095121
PP 5*PP 9	-0.38516	0.124599	69.9	-3.09	0.0029*	-0.592858	-0.177462
PP 6*PP 9	0.4807861	0.1266505	70.1	3.80	0.0003*	0.2696745	0.6918977
PP 7	-0.089344	0.0443008	70.0	-2.02	0.0476*	-0.16319	-0.015498
PP 8	-0.083775	0.0459624	70.1	-1.82	0.0726	-0.160389	-0.00716
PP 8*PP 8	0.5822485	0.1082691	74.1	5.38	<.0001*	0.4019056	0.7625913
PP 9	-0.551804	0.1248424	70.4	-4.42	<.0001*	-0.759888	-0.343719
PP 9*PP 9	-0.712343	0.1324882	71.9	-5.38	<.0001*	-0.933112	-0.491574

-2 Residual Log Likelihood	62.219044

Random Coefficients

Workpackage

Workpackage	Intercept
WP1	0.0689808
WP2	-0.077101
WP3	0.0081205
WP4	-0.074742
WP5	-0.475544
WP6	0.3736893
WP7	0.1765965

Covariance Matrix

Random Effect	Intercept
Intercept	0.091102

Fit Group

Mixed Model for CQA2 - WP1-7

Fixed Effects Tests

Source	Nparm	DFNum	DFDen	F Ratio	Prob > F
Analytical Method	1	1	5.1	21.205028	0.0054*
PP 1	1	1	70.9	13.787897	0.0004*
PP 2	1	1	70.0	33.88763	<.0001*
PP 5	1	1	70.5	19.006454	<.0001*
PP 7	1	1	70.0	4.067322	0.0476*
PP 8	1	1	70.1	3.3221643	0.0726
PP 9	1	1	70.4	19.536422	<.0001*
PP 1*PP 1	1	1	70.3	4.3963967	0.0396*
PP 4*PP 4	1	1	70.2	20.522085	<.0001*
PP 4*PP 5	1	1	70.2	3.867756	0.0532
PP 1*PP 6	1	1	70.2	8.2397148	0.0054*
PP 2*PP 6	1	1	70.1	8.3516865	0.0051*
PP 2*PP 7	1	1	70.3	20.807145	<.0001*
PP 8*PP 8	1	1	74.1	28.920618	<.0001*
PP 1*PP 9	1	1	70.6	33.133142	<.0001*
PP 3*PP 9	1	1	69.9	10.718404	0.0017*
PP 4*PP 9	1	1	70.4	59.630107	<.0001*
PP 5*PP 9	1	1	69.9	9.5555049	0.0029*
PP 6*PP 9	1	1	70.1	14.41087	0.0003*
PP 9*PP 9	1	1	71.9	28.908439	<.0001*
PP 4*PP 4*PP 4	1	1	70.6	566.07377	<.0001*

Residual Plots

Residual by Predicted Plot

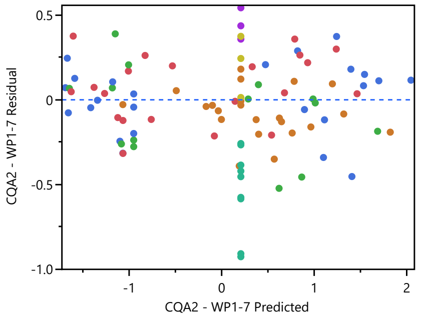

Residual Quantile Plot

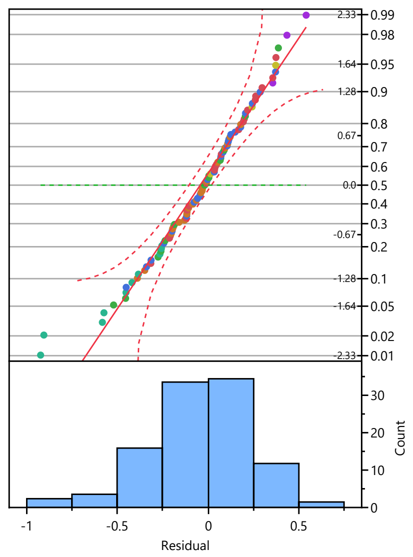

Residual by Row Plot

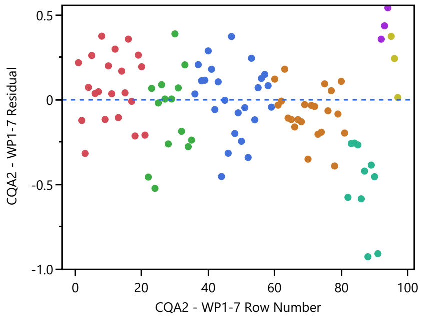

Fit Group

Mixed Model for CQA2 - WP1-7

Conditional Residual Plots

Conditional Residual by Predicted Plot

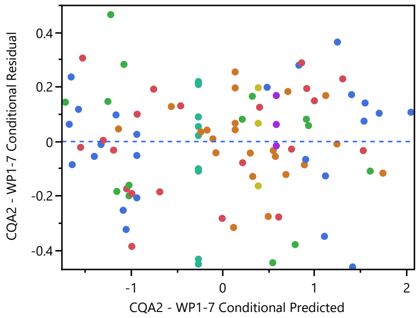

Conditional Residual Quantile Plot

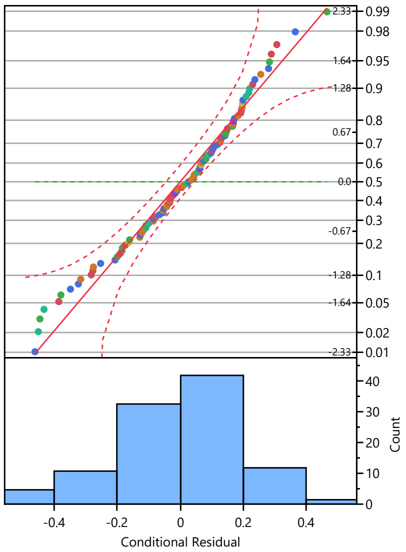

Conditional Residual by Row Plot

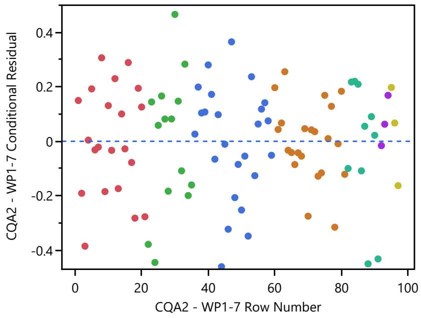

Marginal Model Profiler

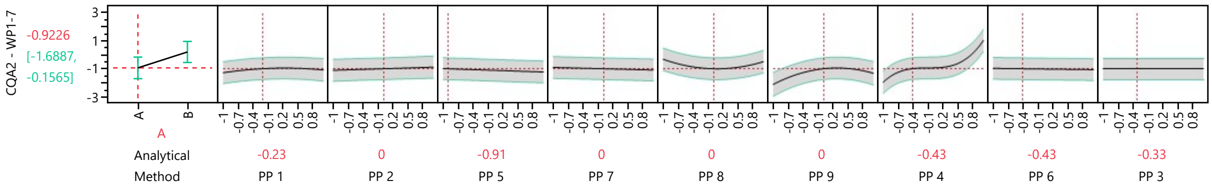

Mixed Model for CQA3 - WP1-7

Actual by Predicted Plot

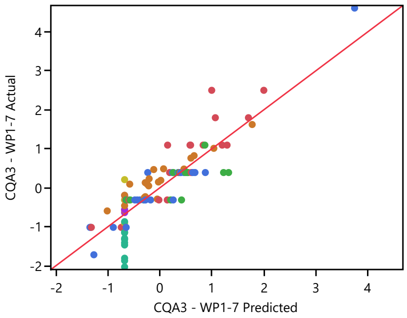

Actual by Conditional Predicted Plot

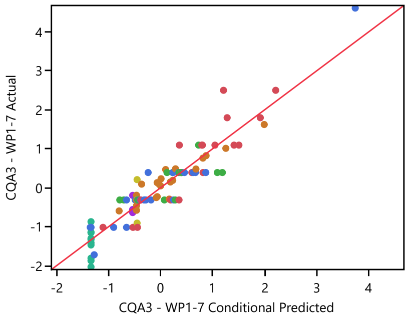

Fit Statistics

Number of rows	97
Sum of Frequencies	97
-2 Log Likelihood	106.4329
AICc	186.31525
BIC	234.5248

Random Effects Covariance Parameter Estimates

Variance Component	Var Ratio	Estimate	Std Error	90% Lower	90% Upper	Pct of Total
Workpackage	0.65251	0.128551	0.0887861	0.0550053	0.6827869	39.486
Residual		0.1970098	0.0344101	0.1511317	0.2694834	60.514
Total		0.3255609	0.0940909	0.2144755	0.5646315	100.000

Fit Group

Mixed Model for CQA3 - WP1-7

Fixed Effects Parameter Estimates

Indicator Coding

Term	Estimate	Std Error	DFDen	t Ratio	Prob>|t|	90% Lower	90% Upper
Intercept	-0.039099	0.1774842	11.4	-0.22	0.8295	-0.356907	0.2787091
PP 1	-0.196065	0.0935338	66.8	-2.10	0.0399*	-0.352078	-0.040052
PP 2	-0.314987	0.1041983	66.1	-3.02	0.0036*	-0.488813	-0.141162
PP 5	0.6071274	0.0703367	69.2	8.63	<.0001*	0.4898633	0.7243915
PP 8	-0.463549	0.0944449	66.5	-4.91	<.0001*	-0.621091	-0.306007
PP 9	-0.964505	0.200308	65.7	-4.82	<.0001*	-1.298692	-0.630319
PP 1*PP 2	0.3894625	0.1598276	65.8	2.44	0.0175*	0.1228171	0.6561078
PP 2*PP 4	0.3846401	0.12044	66.3	3.19	0.0022*	0.1837253	0.585555
PP 4*PP 4	0.6933579	0.1628892	69.2	4.26	<.0001*	0.4217953	0.9649204
PP 1*PP 5	-0.210547	0.0849385	66.3	-2.48	0.0157*	-0.352238	-0.068856
PP 2*PP 5	-0.241677	0.1123191	65.7	-2.15	0.0351*	-0.429067	-0.054286
PP 4*PP 5	-0.497959	0.0793117	67.5	-6.28	<.0001*	-0.630231	-0.365687
PP 1*PP 6	0.2168111	0.1018336	65.7	2.13	0.0370*	0.0469129	0.3867093
PP 4*PP 6	0.2259047	0.110244	66.5	2.05	0.0444*	0.0420081	0.4098014
PP 5*PP 6	-0.219411	0.1035437	67.6	-2.12	0.0378*	-0.392091	-0.046731
PP 1*PP 7	-0.20342	0.0986676	65.8	-2.06	0.0432*	-0.368032	-0.038807
PP 3*PP 7	-0.702055	0.1670687	68.3	-4.20	<.0001*	-0.980635	-0.423474
PP 4*PP 7	-0.260824	0.0987809	65.9	-2.64	0.0103*	-0.425619	-0.096028
PP 5*PP 7	0.2202972	0.0935311	66.0	2.36	0.0215*	0.0642621	0.3763324
PP 2*PP 8	-0.457736	0.2118071	65.8	-2.16	0.0343*	-0.8111	-0.104372
PP 3*PP 8	-0.483374	0.1669462	67.5	-2.90	0.0051*	-0.761797	-0.20495
PP 6*PP 8	0.2989685	0.1078518	65.6	2.77	0.0072*	0.1190259	0.4789112
PP 7*PP 8	-0.335444	0.0969623	66.3	-3.46	0.0010*	-0.497194	-0.173694
PP 8*PP 8	-0.666837	0.2003823	68.6	-3.33	0.0014*	-1.000952	-0.332723
PP 1*PP 9	-1.307258	0.3419739	67.2	-3.82	0.0003*	-1.877618	-0.736898
PP 5*PP 9	-0.941551	0.2077413	66.4	-4.53	<.0001*	-1.288091	-0.595011

-2 Residual Log Likelihood	171.77331

Effect Coding

Term	Estimate	Std Error	DFDen	t Ratio	Prob>|t|	90% Lower	90% Upper
Intercept	-0.039099	0.1774842	11.4	-0.22	0.8295	-0.356907	0.2787091
PP 1	-0.196065	0.0935338	66.8	-2.10	0.0399*	-0.352078	-0.040052
PP 2	-0.314987	0.1041983	66.1	-3.02	0.0036*	-0.488813	-0.141162
PP 5	0.6071274	0.0703367	69.2	8.63	<.0001*	0.4898633	0.7243915
PP 8	-0.463549	0.0944449	66.5	-4.91	<.0001*	-0.621091	-0.306007
PP 9	-0.964505	0.200308	65.7	-4.82	<.0001*	-1.298692	-0.630319
PP 1*PP 2	0.3894625	0.1598276	65.8	2.44	0.0175*	0.1228171	0.6561078
PP 2*PP 4	0.3846401	0.12044	66.3	3.19	0.0022*	0.1837253	0.585555
PP 4*PP 4	0.6933579	0.1628892	69.2	4.26	<.0001*	0.4217953	0.9649204
PP 1*PP 5	-0.210547	0.0849385	66.3	-2.48	0.0157*	-0.352238	-0.068856
PP 2*PP 5	-0.241677	0.1123191	65.7	-2.15	0.0351*	-0.429067	-0.054286
PP 4*PP 5	-0.497959	0.0793117	67.5	-6.28	<.0001*	-0.630231	-0.365687
PP 1*PP 6	0.2168111	0.1018336	65.7	2.13	0.0370*	0.0469129	0.3867093
PP 4*PP 6	0.2259047	0.110244	66.5	2.05	0.0444*	0.0420081	0.4098014
PP 5*PP 6	-0.219411	0.1035437	67.6	-2.12	0.0378*	-0.392091	-0.046731
PP 1*PP 7	-0.20342	0.0986676	65.8	-2.06	0.0432*	-0.368032	-0.038807
PP 3*PP 7	-0.702055	0.1670687	68.3	-4.20	<.0001*	-0.980635	-0.423474
PP 4*PP 7	-0.260824	0.0987809	65.9	-2.64	0.0103*	-0.425619	-0.096028
PP 5*PP 7	0.2202972	0.0935311	66.0	2.36	0.0215*	0.0642621	0.3763324
PP 2*PP 8	-0.457736	0.2118071	65.8	-2.16	0.0343*	-0.8111	-0.104372
PP 3*PP 8	-0.483374	0.1669462	67.5	-2.90	0.0051*	-0.761797	-0.20495
PP 6*PP 8	0.2989685	0.1078518	65.6	2.77	0.0072*	0.1190259	0.4789112
PP 7*PP 8	-0.335444	0.0969623	66.3	-3.46	0.0010*	-0.497194	-0.173694
PP 8*PP 8	-0.666837	0.2003823	68.6	-3.33	0.0014*	-1.000952	-0.332723
PP 1*PP 9	-1.307258	0.3419739	67.2	-3.82	0.0003*	-1.877618	-0.736898
PP 5*PP 9	-0.941551	0.2077413	66.4	-4.53	<.0001*	-1.288091	-0.595011

-2 Residual Log Likelihood	171.77331

Fixed Effects Tests

Source	Nparm	DFNum	DFDen	F Ratio	Prob > F
PP 1	1	1	66.8	4.3940109	0.0399*
PP 2	1	1	66.1	9.1382959	0.0036*
PP 5	1	1	69.2	74.506678	<.0001*
PP 8	1	1	66.5	24.089852	<.0001*
PP 9	1	1	65.7	23.185299	<.0001*
PP 1*PP 2	1	1	65.8	5.9378297	0.0175*
PP 2*PP 4	1	1	66.3	10.199237	0.0022*
PP 4*PP 4	1	1	69.2	18.118846	<.0001*
PP 1*PP 5	1	1	66.3	6.1445314	0.0157*
PP 2*PP 5	1	1	65.7	4.629806	0.0351*
PP 4*PP 5	1	1	67.5	39.419649	<.0001*
PP 1*PP 6	1	1	65.7	4.5329499	0.0370*
PP 4*PP 6	1	1	66.5	4.1989497	0.0444*
PP 5*PP 6	1	1	67.6	4.4902525	0.0378*
PP 1*PP 7	1	1	65.8	4.2504675	0.0432*
PP 3*PP 7	1	1	68.3	17.658437	<.0001*
PP 4*PP 7	1	1	65.9	6.9718624	0.0103*
PP 5*PP 7	1	1	66.0	5.5476052	0.0215*
PP 2*PP 8	1	1	65.8	4.6703381	0.0343*
PP 3*PP 8	1	1	67.5	8.3832608	0.0051*
PP 6*PP 8	1	1	65.6	7.684162	0.0072*
PP 7*PP 8	1	1	66.3	11.968356	0.0010*
PP 8*PP 8	1	1	68.6	11.074419	0.0014*
PP 1*PP 9	1	1	67.2	14.612906	0.0003*
PP 5*PP 9	1	1	66.4	20.541977	<.0001*

Fit Group

Mixed Model for CQA3 - WP1-7

Residual Plots

Residual by Predicted Plot

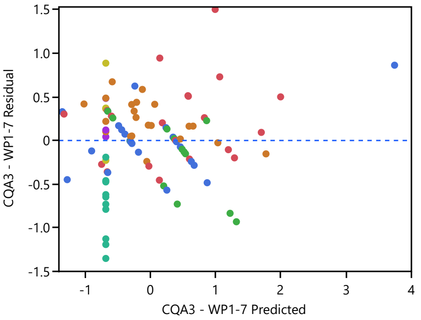

Residual by Row Plot

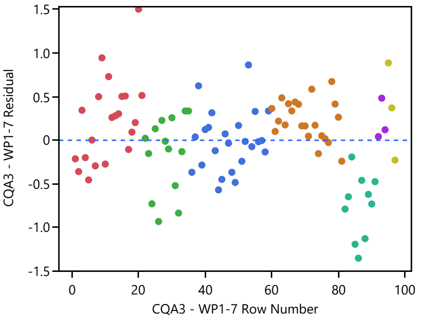

Residual Quantile Plot

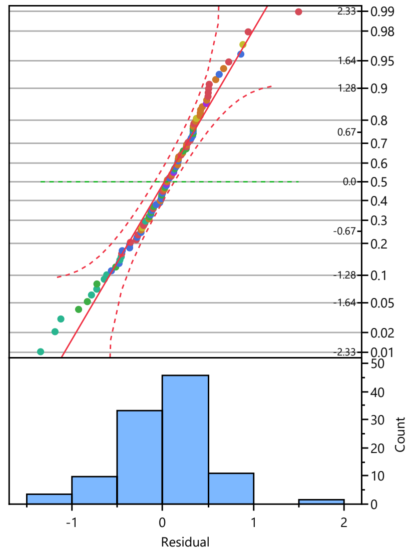

Conditional Residual Plots

Conditional Residual by Predicted Plot

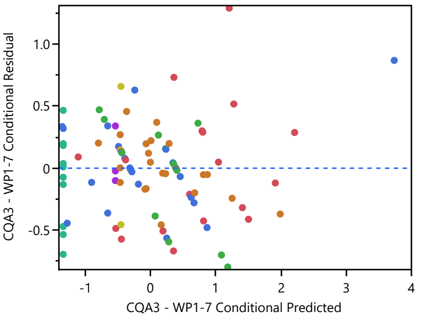

Conditional Residual by Row Plot

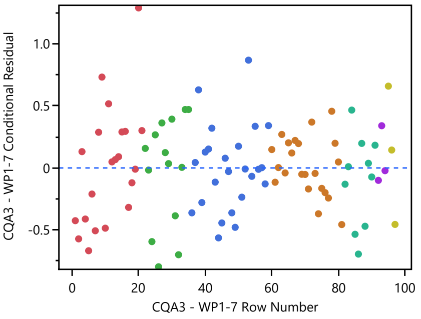

Conditional Residual Quantile Plot

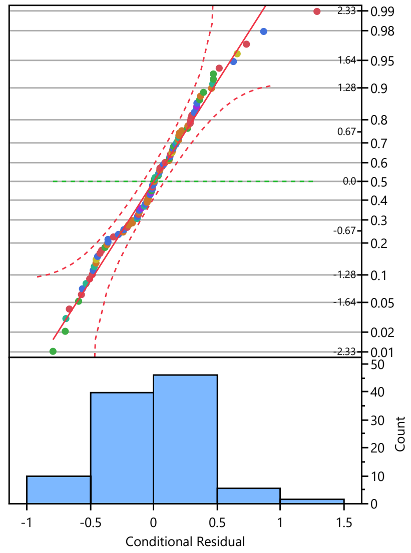

Marginal Model Profiler

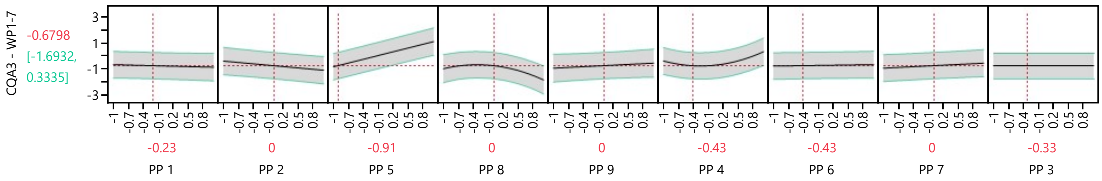

Graph Builder

Measured & Prediction vs. Experiment

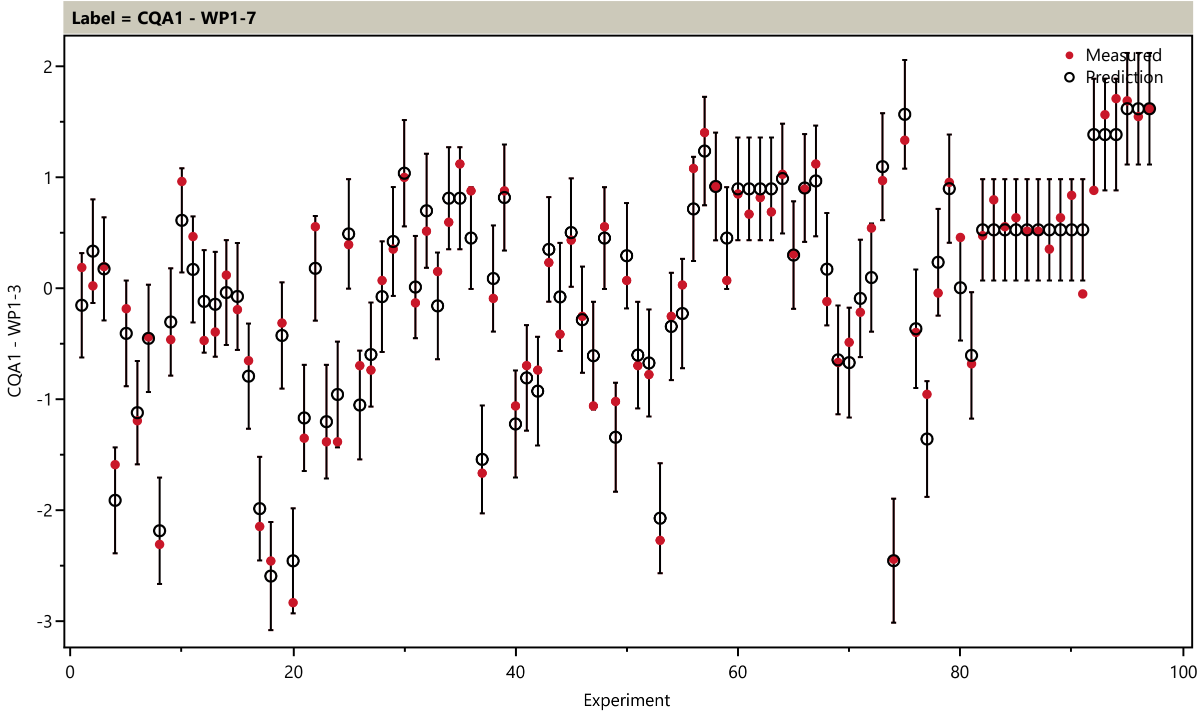

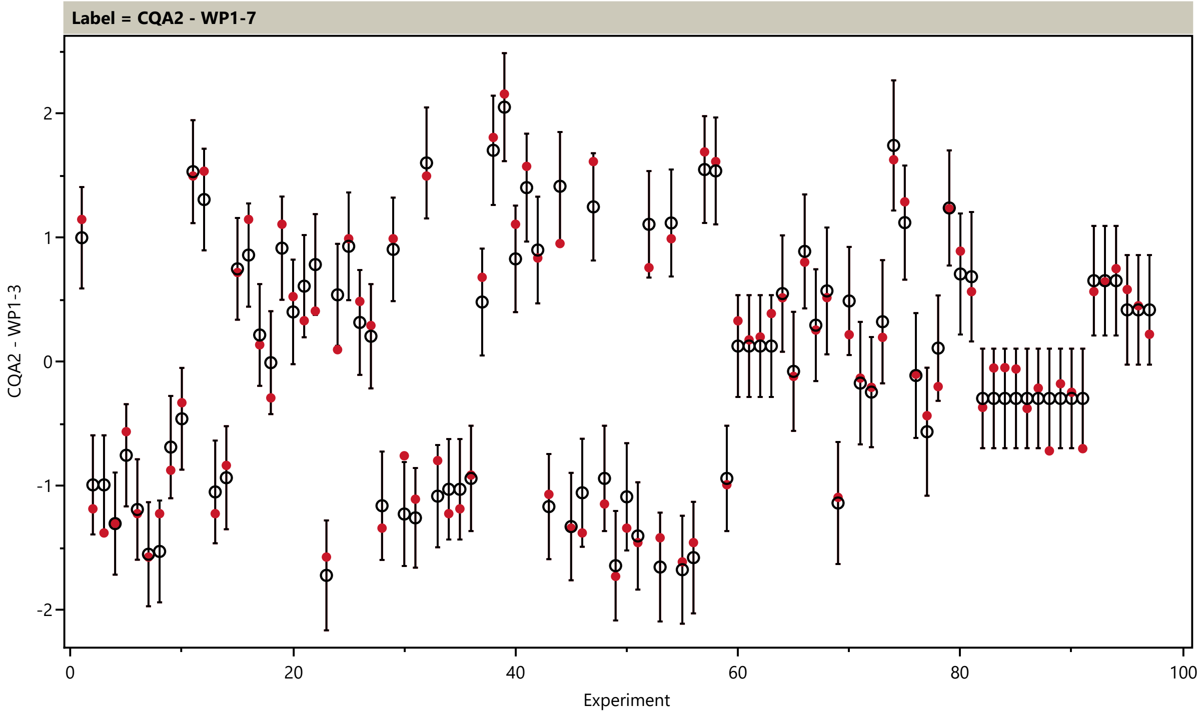

Graph Builder

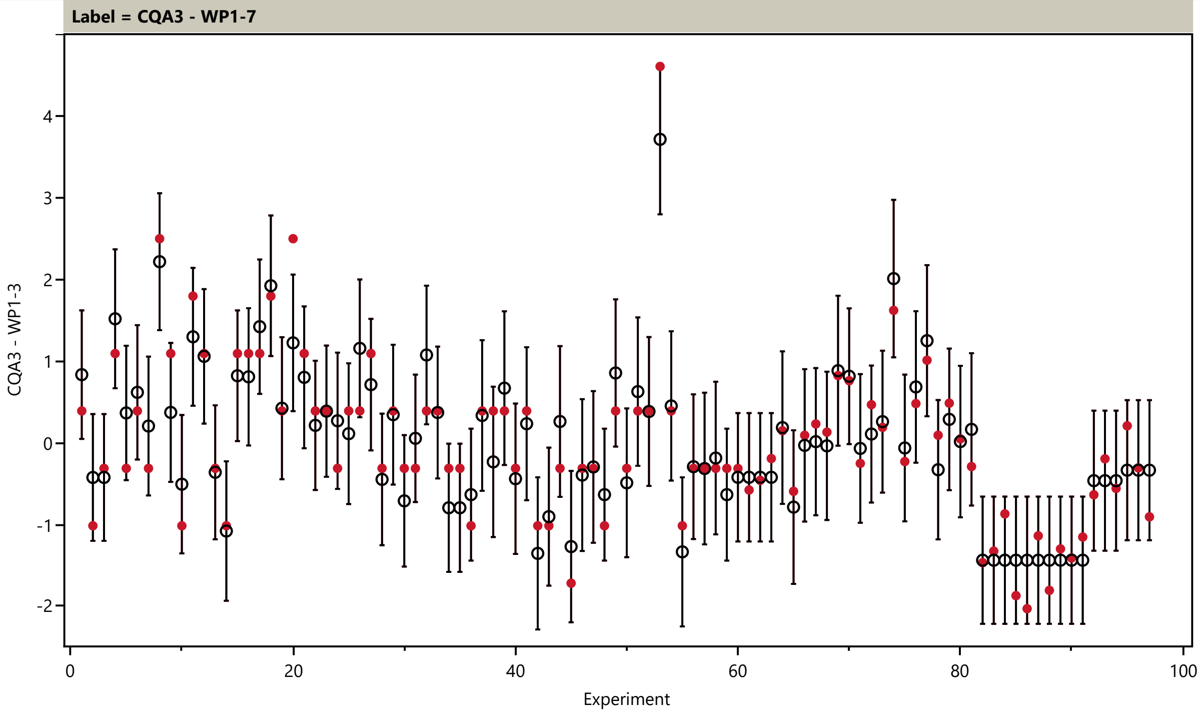

Each error bar is constructed from 90% lower PI to 90% upper PI.

Graph Builder

Measured & Prediction vs. Experiment

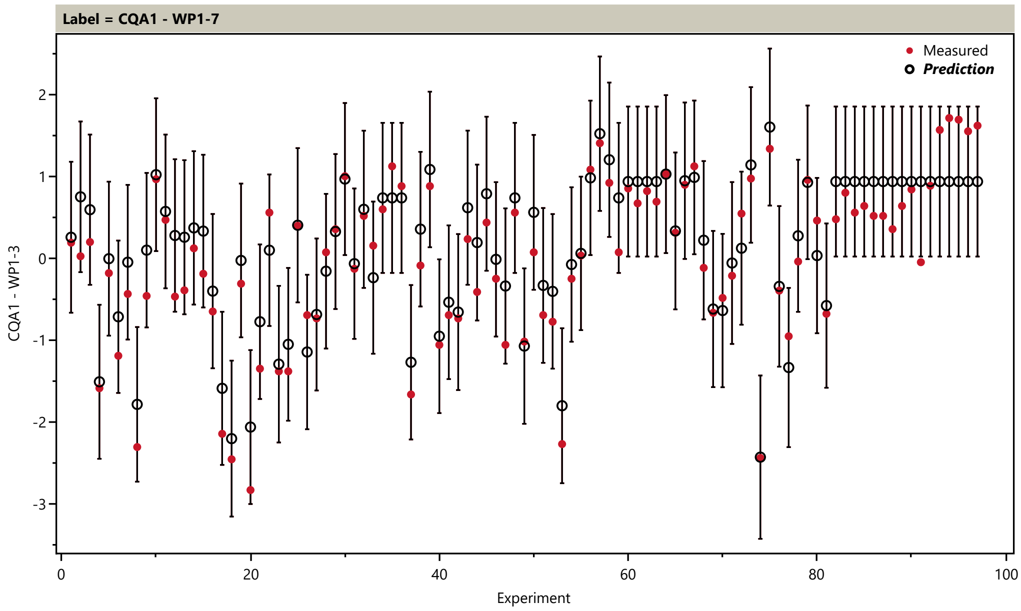

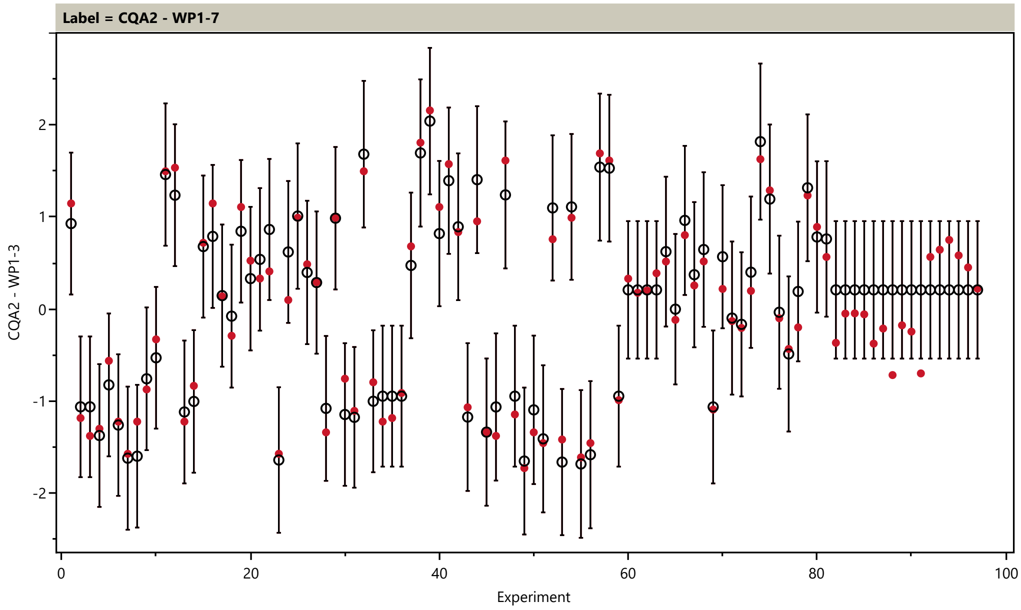

Graph Builder

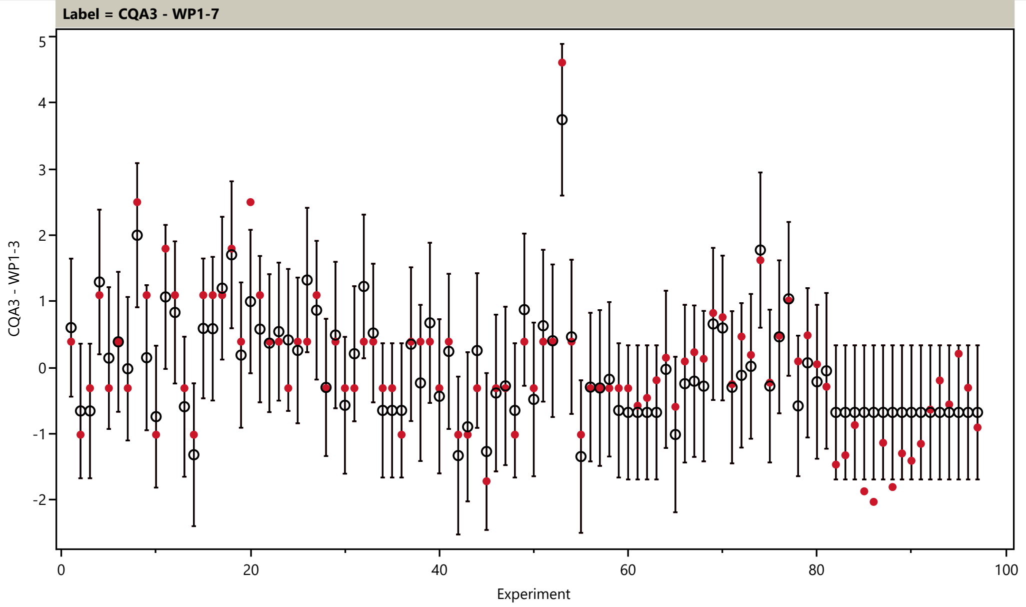

Each error bar is constructed from 90% lower PI to 90% upper PI.

Evaluate Design

Design

Run	Workpackage	PP 1	PP 2	PP 3	PP 4	PP 5	PP 6	PP 7	PP 8	PP 9
1	WP1	-0.53846	0	-0.33333	1	-0.90909	0.142857	0	0	0
2	WP1	-0.53846	0	1	-0.42857	-0.90909	0.142857	0	0	0
3	WP1	-0.53846	0	-0.33333	-0.42857	-0.90909	0.142857	0	0	0
4	WP1	0.384615	-1	-0.33333	-0.42857	1	0.142857	0	0	0
5	WP1	-0.07692	1	-0.33333	-0.42857	1	0.142857	0	0	0
6	WP1	0.384615	-1	1	-0.42857	0.048485	0.142857	0	0	0
7	WP1	-1	-1	1	-0.42857	-0.90909	0.142857	0	0	0
8	WP1	-0.53846	-1	1	-0.42857	1	0.142857	0	0	0
9	WP1	0.384615	1	1	-0.42857	1	0.142857	0	0	0
10	WP1	0.384615	1	-0.33333	-0.42857	-0.90909	0.142857	0	0	0
11	WP1	0.384615	1	1	1	-0.90909	0.142857	0	0	0
12	WP1	0.384615	1	-0.33333	1	0.048485	0.142857	0	0	0
13	WP1	-1	1	1	-0.42857	0.048485	0.142857	0	0	0
14	WP1	-1	1	-0.33333	-0.42857	-0.90909	0.142857	0	0	0
15	WP1	-1	0	1	1	-0.90909	0.142857	0	0	0
16	WP1	-0.53846	1	1	1	1	0.142857	0	0	0
17	WP1	-1	-1	-0.33333	1	0.048485	0.142857	0	0	0
18	WP1	-1	-1	1	1	1	0.142857	0	0	0
19	WP1	0.384615	-1	-0.33333	1	-0.90909	0.142857	0	0	0
20	WP1	-0.07692	-1	-0.33333	1	1	0.142857	0	0	0
21	WP1	-1	1	-0.33333	1	1	0.142857	0	0	0
22	WP2	0.384615	0	-0.33333	1	0.048485	-0.71429	0	0	0
23	WP2	-0.07692	-1	-0.33333	-0.42857	0.048485	1	0	0	0
24	WP2	0.384615	-1	1	1	0.048485	0.142857	0	0	0
25	WP2	-0.53846	-1	1	1	-0.90909	-0.71429	0	0	0
26	WP2	-1	0	1	1	1	-0.71429	0	0	0
27	WP2	-1	0	-0.33333	1	0.048485	1	0	0	0
28	WP2	-0.53846	1	-0.33333	-0.42857	0.048485	-0.71429	0	0	0
29	WP2	-1	1	1	1	-0.90909	0.142857	0	0	0
30	WP2	-1	0	1	-0.42857	-0.90909	-0.71429	0	0	0
31	WP2	-0.53846	0	-0.33333	-0.42857	0.048485	0.142857	0	0	0
32	WP2	-0.07692	1	1	1	-0.90909	1	0	0	0
33	WP2	0.384615	0	1	-0.42857	1	1	0	0	0
34	WP2	-0.23077	0	-0.33333	-0.42857	-0.90909	0.142857	0	0	0
35	WP2	-0.23077	0	-0.33333	-0.42857	-0.90909	0.142857	0	0	0
36	WP3	-0.23077	0	-0.33333	-0.42857	-0.90909	0.142857	0	0	0
37	WP3	-1	0	-0.33333	1	1	1	1	1	0
38	WP3	1	0	-0.33333	1	-1	1	1	1	0
39	WP3	1	0	-0.33333	1	-1	1	-1	-1	0
40	WP3	-1	0	-0.33333	1	1	1	-1	-1	0
41	WP3	1	0	-0.33333	1	1	1	1	-1	0
42	WP3	1	0	-0.33333	1	1	-0.71429	1	1	0
43	WP3	1	0	-0.33333	-1	-1	1	0	1	0
44	WP3	1	0	-0.33333	1	1	1	-1	1	0
45	WP3	-1	0	-0.33333	-1	-1	-0.71429	-1	-1	0
46	WP3	1	0	-0.33333	-1	1	1	-1	-1	0
47	WP3	1	0	-0.33333	1	1	-0.71429	-1	-1	0
48	WP3	-0.23077	0	-0.33333	-0.42857	-0.90909	0.142857	0	0	0
49	WP3	-1	0	-0.33333	-1	1	-0.71429	-1	1	0
50	WP3	1	0	-0.33333	-1	-1	1	1	-1	0
51	WP3	1	0	-0.33333	-1	1	1	1	1	0
52	WP3	-1	0	-0.33333	1	-0.95152	1	1	-1	0
53	WP3	-1	0	-0.33333	-1	1	-0.71429	1	-1	0
54	WP3	-1	0	-0.33333	1	-0.95152	1	-1	1	0
55	WP3	-1	0	-0.33333	-1	-1	-0.71429	1	1	0
56	WP3	1	0	-0.33333	-1	-1	1	-1	0	0
57	WP3	1	0	-0.33333	1	-1	-0.71429	-1	1	0
58	WP3	1	0	-0.33333	1	-1	-0.71429	1	-1	0
59	WP3	-0.23077	0	-0.33333	-0.42857	-0.90909	0.142857	0	0	0
60	WP4	-0.23077	0	-0.33333	-0.42857	-0.90909	-0.42857	0	0	0
61	WP4	-0.23077	0	-0.33333	-0.42857	-0.90909	-0.42857	0	0	0
62	WP4	-0.23077	0	-0.33333	-0.42857	-0.90909	-0.42857	0	0	0
63	WP4	-0.23077	0	-0.33333	-0.42857	-0.90909	-0.42857	0	0	0
64	WP4	-0.53846	-1	1	-0.42857	-1	-0.42857	1	-1	1
65	WP4	-0.07692	-1	-1	-0.42857	0.048485	-0.42857	-1	1	1
66	WP4	-1	0	1	-0.42857	0.048485	-1	-1	1	1
67	WP4	-0.53846	1	1	-0.71429	-0.95152	0.142857	-1	-1	0
68	WP4	0.384615	0	-0.33333	-0.14286	-0.90909	-1	0	0	-1
69	WP4	-0.07692	-1	-1	-1	0.048485	0.142857	1	-1	1
70	WP4	-0.07692	0	-0.33333	-0.14286	1	1	1	-1	0
71	WP4	-0.53846	-1	1	-0.42857	-0.90909	-1	-1	-1	-1
72	WP4	-0.53846	-1	0.333333	-0.71429	-0.90909	-0.42857	1	-1	0

Evaluate Design

Design

Run	Workpackage	PP 1	PP 2	PP 3	PP 4	PP 5	PP 6	PP 7	PP 8	PP 9
73	WP4	-0.53846	0	-1	-0.71429	-1	0.142857	0	0	1
74	WP4	-1	0	-1	1	1	1	1	-1	-1
75	WP4	-0.53846	1	0.333333	-0.42857	-0.90909	0.142857	-1	-1	1
76	WP4	-1	1	1	-0.71429	1	-1	0	0	1
77	WP4	0.384615	0	-0.33333	-0.71429	0.048485	1	0	1	-1
78	WP4	-0.53846	0	-1	-0.14286	0.048485	-1	-1	0	0
79	WP4	-0.53846	-1	1	-0.14286	-0.90909	-0.42857	-1	-1	0
80	WP4	-0.07692	-1	1	-0.71429	0.048485	0.142857	-1	1	1
81	WP4	-0.53846	0	1	1	1	1	-1	-1	1
82	WP5	-0.23077	0	-0.33333	-0.42857	-0.90909	-0.42857	0	0	0
83	WP5	-0.23077	0	-0.33333	-0.42857	-0.90909	-0.42857	0	0	0
84	WP5	-0.23077	0	-0.33333	-0.42857	-0.90909	-0.42857	0	0	0
85	WP5	-0.23077	0	-0.33333	-0.42857	-0.90909	-0.42857	0	0	0
86	WP5	-0.23077	0	-0.33333	-0.42857	-0.90909	-0.42857	0	0	0
87	WP5	-0.23077	0	-0.33333	-0.42857	-0.90909	-0.42857	0	0	0
88	WP5	-0.23077	0	-0.33333	-0.42857	-0.90909	-0.42857	0	0	0
89	WP5	-0.23077	0	-0.33333	-0.42857	-0.90909	-0.42857	0	0	0
90	WP5	-0.23077	0	-0.33333	-0.42857	-0.90909	-0.42857	0	0	0
91	WP5	-0.23077	0	-0.33333	-0.42857	-0.90909	-0.42857	0	0	0
92	WP6	-0.23077	0	-0.33333	-0.42857	-0.90909	-0.42857	0	0	0
93	WP6	-0.23077	0	-0.33333	-0.42857	-0.90909	-0.42857	0	0	0
94	WP6	-0.23077	0	-0.33333	-0.42857	-0.90909	-0.42857	0	0	0
95	WP7	-0.23077	0	-0.33333	-0.42857	-0.90909	-0.42857	0	0	0
96	WP7	-0.23077	0	-0.33333	-0.42857	-0.90909	-0.42857	0	0	0
97	WP7	-0.23077	0	-0.33333	-0.42857	-0.90909	-0.42857	0	0	0

Design Evaluation

Power Analysis

Significance Level 0.05

Anticipated RMSE 1

Term	Anticipated Coefficient	Power
Intercept	1	0.335
Workpackage 1	1	0.453
Workpackage 2	-1	0.552
Workpackage 3	1	0.471
Workpackage 4	-1	0.627
Workpackage 5	1	0.743
Workpackage 6	-1	0.421
PP 1	4.058596	1
PP 2	99	1
PP 3	5.968524	1
PP 4	6.997579	1
PP 5	22.54776	1
PP 6	3.560172	1
PP 7	2.536622	1
PP 8	2.536622	1
PP 9	4.058596	0.999
PP 1*PP 1	4.227704	1
PP 1*PP 2	99	1
PP 1*PP 3	4.911176	1
PP 1*PP 4	4.755796	1
PP 1*PP 5	3.89725	1
PP 1*PP 6	3.215494	1
PP 1*PP 7	3.474825	1
PP 1*PP 8	3.474825	1
PP 1*PP 9	5.559721	0.978
PP 2*PP 2	99	1
PP 2*PP 3	99	1
PP 2*PP 4	99	1
PP 2*PP 5	99	1
PP 2*PP 6	99	1
PP 2*PP 7	99	1
PP 2*PP 8	99	1
PP 2*PP 9	99	1
PP 3*PP 3	5.968524	1
PP 3*PP 4	5.959759	1
PP 3*PP 5	5.48904	1
PP 3*PP 6	3.84264	1
PP 3*PP 7	3.786004	1
PP 3*PP 8	3.786004	1

Evaluate Design

Design Evaluation

Power Analysis

Term	Anticipated Coefficient	Power
PP 3*PP 9	6.057606	1
PP 4*PP 4	6.084852	1
PP 4*PP 5	6.173709	1
PP 4*PP 6	3.792372	1
PP 4*PP 7	3.523087	1
PP 4*PP 8	3.523087	1
PP 4*PP 9	5.636939	0.984
PP 5*PP 5	11.80511	1
PP 5*PP 6	3.333823	1
PP 5*PP 7	2.536622	1
PP 5*PP 8	2.536622	1
PP 5*PP 9	4.058596	0.999
PP 6*PP 6	2.489631	0.882
PP 6*PP 7	2.536622	1
PP 6*PP 8	2.536622	1
PP 6*PP 9	4.058596	0.999
PP 7*PP 7	3.170778	0.953
PP 7*PP 8	3.170778	1
PP 7*PP 9	5.073245	1
PP 8*PP 8	3.170778	0.902
PP 8*PP 9	5.073245	1
PP 9*PP 9	8.117192	1

Effect	Power
Workpackage	0.988

Color Map on Correlations

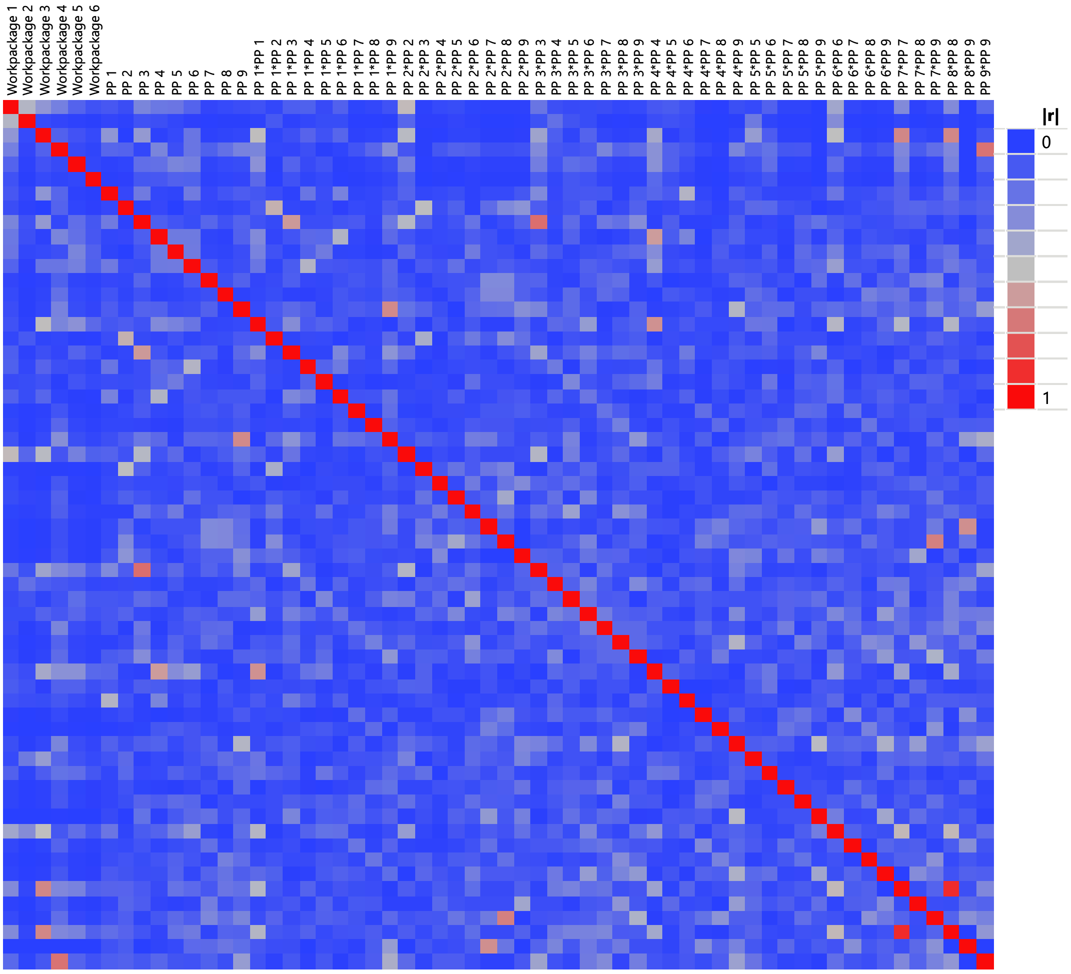

Output Options
